# Mapping the Global Emergence of *Batrachochytrium dendrobatidis*, the Amphibian Chytrid Fungus

**DOI:** 10.1371/journal.pone.0056802

**Published:** 2013-02-27

**Authors:** Deanna H. Olson, David M. Aanensen, Kathryn L. Ronnenberg, Christopher I. Powell, Susan F. Walker, Jon Bielby, Trenton W. J. Garner, George Weaver, The Bd Mapping Group, Matthew C. Fisher

**Affiliations:** 1 Pacific Northwest Research Station, U.S. Forest Service, Corvallis, Oregon, United States of America; 2 Department of Infectious Disease Epidemiology, Imperial College, London, United Kingdom; 3 Institute of Zoology, Zoological Society of London, London, United Kingdom; 4 Department of Statistics, Oregon State University, Corvallis, Oregon, United States of America; University of California Riverside, United States of America

## Abstract

The rapid worldwide emergence of the amphibian pathogen *Batrachochytrium dendrobatidis* (*Bd*) is having a profound negative impact on biodiversity. However, global research efforts are fragmented and an overarching synthesis of global infection data is lacking. Here, we provide results from a community tool for the compilation of worldwide *Bd* presence and report on the analyses of data collated over a four-year period. Using this online database, we analysed: 1) spatial and taxonomic patterns of infection, including amphibian families that appear over- and under-infected; 2) relationships between *Bd* occurrence and declining amphibian species, including associations among *Bd* occurrence, species richness, and enigmatic population declines; and 3) patterns of environmental correlates with *Bd*, including climate metrics for all species combined and three families (Hylidae, Bufonidae, Ranidae) separately, at both a global scale and regional (U.S.A.) scale. These associations provide new insights for downscaled hypothesis testing. The pathogen has been detected in 52 of 82 countries in which sampling was reported, and it has been detected in 516 of 1240 (42%) amphibian species. We show that detected *Bd* infections are related to amphibian biodiversity and locations experiencing rapid enigmatic declines, supporting the hypothesis that greater complexity of amphibian communities increases the likelihood of emergence of infection and transmission of *Bd*. Using a global model including all sampled species, the odds of *Bd* detection decreased with increasing temperature range at a site. Further consideration of temperature range, rather than maximum or minimum temperatures, may provide new insights into *Bd*-host ecology. Whereas caution is necessary when interpreting such a broad global dataset, the use of our pathogen database is helping to inform studies of the epidemiology of *Bd*, as well as enabling regional, national, and international prioritization of conservation efforts. We provide recommendations for adaptive management to enhance the database utility and relevance.

## Introduction

The specter of multiple-host infectious diseases leading to the rapid devastation of entire communities is alarming but, so far, rare. However, a handful of highly virulent multi-host pathogens are known, and these have had profound impacts across populations and species (e.g., the rinderpest Morbillivirus; West Nile Virus; bat *Geomyces destructans*). Rapid aggregation, synthesis, and analysis of disease data are needed to advance both science and management of such diseases. While technological advances have aided our ability to respond to emerging disease, such as by the use of proactive modeling [1], our ability to rapidly detect, assess, and report globally-emerging pathogens has lagged for wild animal diseases that are not zoonotic or are not transmitted to livestock or companion animals. For these diseases, infection and mortality are often cryptic and research studies may take years to be completed before being published.

Herein we report on a community surveillance effort addressing the emergence of the amphibian chytrid fungus *Batrachochytrium dendrobatidis* (*Bd*). Approximately one-third of global amphibian species have imperiled status, and the emergence of *Bd* is known to be a proximate driver of amphibian species declines and extinctions [2]–[8]. *Bd* has an unusually wide host-range: it has been detected infecting hundreds of species. Cascading effects owing to declines of multiple host species as a result of chytridiomycosis could potentially undermine ecosystem stability and function [9], [10], although some studies have not supported key functional roles of amphibians in local ecosystems [11]. Hence, although caution is necessary because amphibians' ecological roles will vary with location, as a worst case scenario, the potential of *Bd* to act synergistically with other anthropogenic drivers may catastrophically disturb biological communities and substantially contribute to the ongoing 6^th^ mass extinction event [8].

Although infectious diseases are a normal component of wildlife ecology, only recently are diseases being widely recognized as critical conservation concerns, notably for amphibians [12]. *Bd* was described as a species in the late 1990s [13], and our basic biological knowledge of this novel pathogen is still accruing. After over a dozen years of research, many aspects of its ecology, epidemiology, and pathogenicity remain uncertain. For example, vectors and pathways of transmission of *Bd* across spatial scales are little understood, as are the factors affecting species-specific susceptibility to the clinical disease, chytridiomycosis [14], [15], although reservoir amphibian hosts have been identified, including some involved in commercial trade of amphibians [16]–[20]. *Bd* genotype affects *Bd* virulence, and population genomics studies have shown that the worldwide emergence of chytridiomycosis has occurred as a consequence of 20^th^-century emergence of an aggressive lineage [16]. Concern has heightened about *Bd* transmission in various arms of the commercial trade in amphibians which annually involves millions of individuals [15], [21]. Transmission pathways may include migratory waterbirds [22], and some regional studies have reported an association of *Bd* occurrence with amphibian species' proximity to human development, suggesting uncharacterised pathways of introduction and transmission [23]. *Bd* has been detected in water samples [24], supporting transmission via fomites. Due to the recent recognition of *Bd* as an emerging infectious disease and an invasive species, *Bd* was listed as a notifiable disease by the World Organization of Animal Health (OIE) in 2009, resulting in international recommendations to forestall further spread via anthropogenic activities [25]. The need for effective surveillance, including rapid data accrual and interpretation in order to identify emerging patterns and processes, is integral to the progression of both *Bd* science and management objectives. In particular, our incomplete understanding of the global distribution of *Bd* is key information that is required to assess the importance of various mechanisms that may have contributed to the rapid emergence of chytridiomycosis [26] or continue to contribute to the ongoing spread of *Bd*.

We report on the outputs of a multi-phase project for compiling worldwide *Bd* data and maintaining an updated system for global-scale assessment of the pathogen, the Global *Bd* Mapping Project [27]. A web-based system (www.Bd-maps.net) for collation of *Bd* incidence and associated metadata was produced [28], with the aims of providing new insights into both *Bd* occurrence patterns and the development of hypotheses for the study of *Bd* ecology and epidemiology, and to inform species- and land-management planning efforts. Using data gleaned from the published literature or submitted as primary data to *Bd*-maps.net by members of the *Bd* Mapping Group over a four year period, we report: 1) spatial and taxonomic patterns of infection, including amphibian families that appear over- and under-infected; 2) relationships between *Bd* occurrence and declining amphibian species, including an analysis of *Bd* occurrence, species richness, and occurrence of enigmatic population declines; and 3) patterns of environmental correlates, including climate metrics for all species and for three families (Hylidae, Bufonidae, Ranidae), at both the global scale and the U.S.A. scale (which has higher spatial resolution of climate metrics allowing downscaled analyses). Our analyses contribute new insights to hypotheses of geographic and climatic relationships with *Bd* occurrence [29]–[37].

## Results

### *Bd*-maps.net

*Bd*-maps.net contains a web-accessible database for data storage, facilities for data uploading, basic summary statistics, and visualisation of spatial data utilising Google Maps and Google Earth. Data are submitted in a number of ways, through: 1) literature review with subsequent geo-coding and direct database submission; 2) direct online data entry by community users; and 3) a smartphone interface for the direct submission of location and metadata using the data gathering tool, EpiCollect [38]. Summaries of *Bd* data can be viewed globally or by country, and all data are now available for community downloading for further off-line analyses such as those presented here (see Materials and Methods).

Cautions expressed in Materials and Methods for use of *Bd*-maps.net data should be reiterated here; in particular, results of analyses should be used to inform predictions for further examination, and are not conclusive due to several forms of potential data bias. Importantly, distribution maps derived from the *Bd*-maps database do not represent the distribution of chytridiomycosis, but locations where *Bd* sampling has occurred, where the fungus has been detected, or where it was not detected. These locations have a suite of potential biases as discussed in Materials and Methods, and we have addressed these biases to some extent by the data aggregations that we have conducted. Type I errors indicate false *Bd*-positive findings, and may occur in the dataset if *Bd* were detected at a site but the assay to determine this was incorrectly run, or was subject to cross-contamination. A Type II error is a false *Bd*-negative finding. This might occur if *Bd* occurs at a site, but was not detected. A low-level *Bd* infection, an insufficient sample size, or the presence of PCR inhibitors, could result in a false negative report [39]. We did not conduct quality assurance procedures to examine data for the use of acceptable swabbing, histology or PCR techniques, or for sample sizes. These types of errors have been increasingly recognized over the last decade, and are likely reduced for surveillance efforts reported in peer-reviewed publications. Nevertheless, in our analyses, by aggregating all data at a site level and by conducting analyses at the 0.5 degree latitude and longitude grid cell size, we may have reduced the chance of both false-positive and false-negative findings by the compilation of results among different studies (and their different sampling and analytical approaches), researchers, species, seasons, or years. An additional consideration is the sample sizes used in our various analyses and summary statistics; the larger samples may represent more robust datasets with less bias. Nevertheless, we offer our results as hypotheses for further testing, and temper our conclusions accordingly.

### Spatial and taxonomic patterns of *Bd* detection

**Figure 1:**
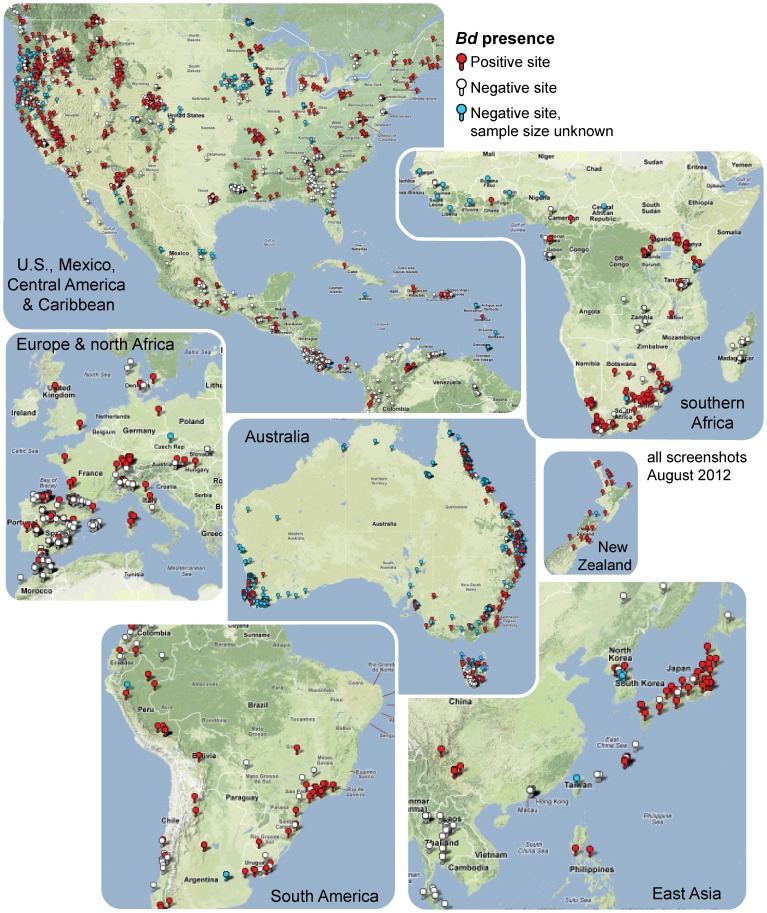
Global distribution of the amphibian chytrid fungus, *Batrachochytrium dendrobatidis* (*Bd*). Maps downloaded from www.Bd-maps.net (15 August 2012); *Bd*-positive (red) and *Bd*-negative (white, blue) sites are shown.

*Bd* has been detected in 56 of 82 (68%) countries (Figure 1), and in 516 of 1,240 (42%) species, determined using a data set of more than 36,000 individuals (*Bd*-maps.net data snapshot August 2012). *Bd* was detected at 1,814 of 4,281 (48%) sites tallied for our analyses (data snapshot July 2010). Broad-scale distribution patterns are evident, with *Bd* widely detected in the Americas, and detected only patchily in Africa, Asia, and Europe. Spatial biases and gaps in sampling across the globe contribute to the clusters of *Bd* locations on the world map. In particular, Asia had been largely unsampled at the time of our data analyses; however, recently, Swei et al. [26] sampled in 15 Asian countries and found only 2.35% *Bd* prevalence.

*Bd* was detected in 41 of 50 (82%) sampled families of amphibians (Table 1; data snapshot January 2011). Anurans and caudates were comparably sampled: 935 of 6,177 (15%) extant and recognized anuran species were included in our study, while 114 of 629 (18%) caudates were also included (Table 1). Caecilians remained relatively unsampled, with only 6 of 190 (3%) species registered in the database (Table 1, Table 2) (total species counts from AmphibiaWeb.org, accessed 25 July 2012). Three speciose anuran families, Hylidae, Ranidae, and Bufonidae, accounted for 30% of the species in which *Bd* was detected (Table 2, Table S1).

**Table 1:** Numbers of amphibian species and families with *Batrachochytrium dendrobatidis* detections as of January 2011.

	Species				Families				
Order	*Bd* Detected	*Bd* Not Detected	Total	Percent Detected	*Bd* Detected	*Bd* Not Detected	Total	Percent Detected	
Anura	449	486	935	48.0	35	6	40	87.5	
Caudata	59	55	114	51.8	6	2	8	75	
Gymnophiona	0	6	6	0	0	2	2	0	
**Total**	**508**	**547**	**1055**	**48.2**	**41**	**9**	**50**	**82**	

**Table 2:** Numbers of species with *Batrachochytrium dendrobatidis* (*Bd*) detections by amphibian family, and results of randomisation tests (see Materials and Methods section) to determine whether each sampled amphibian family was over- or under-infected, compared to what we would expect by chance given an overall prevalence of 508/1055 species.

Family	Species			Prev.	p-value	Prev. pattern
	*Bd* Detected	*Bd* Not Detected	Total Sampled			
***Anura***						
Heleophrynidae	3	0	3	1	0.9652	NA
Pelobatidae	2	0	2	1	0.9278	NA
Calyptocephalellidae	1	0	1	1	0.8494	NA
Ceratophryidae	11	1	12	0.92	0.9988*	**Over**
Hylodidae	8	1	9	0.89	0.9936*	**Over**
Hemiphractidae	7	2	9	0.78	0.9641	NSD
Leiuperidae	6	2	8	0.75	0.9386	NSD
Aromobatidae	6	2	8	0.75	0.9386	NSD
Alytidae	5	2	7	0.71	0.8928	NSD
Pyxicephalidae	11	5	16	0.69	0.9510	NSD
Craugastoridae	17	8	25	0.68	0.9782*	**Over**
Dendrobatidae	14	7	21	0.67	0.9542	NSD
Limnodynastidae	12	6	18	0.67	0.9442	NSD
Cycloramphidae	4	2	6	0.67	0.8137	NSD
Pipidae	10	6	16	0.63	0.8761	NSD
Hylidae	89	59	148	0.6	0.9992*	**Over**
Myobatrachidae	20	14	34	0.59	0.8980	NSD
Ranidae	55	43	98	0.56	0.9513	NSD
Eleutherodactylidae	15	12	27	0.56	0.7858	NSD
Centrolenidae	9	7	16	0.56	0.7361	NSD
Hyperoliidae	27	27	54	0.50	0.6162	NSD
Bombinatoridae	2	2	4	0.50	0.5309	NSD
Strabomantidae	30	33	63	0.48	0.4610	NSD
Leptodactylidae	8	10	18	0.44	0.3776	NSD
Leiopelmatidae	2	3	6	0.40	0.3572	NSD
Bufonidae	43	68	111	0.39	0.0178*	**Under**
Ptychadenidae	5	8	13	0.38	0.2439	NSD
Phrynobatrachidae	5	8	13	0.38	0.2418	NSD
Dicroglossidae	6	15	21	0.29	0.0333	NSD
Rhacophoridae	5	14	19	0.26	0.0286	NSD
Petropedetidae	1	5	6	0.17	0.0643	NSD
Scaphiopodidae	1	5	6	0.17	0.0608	NSD
Microhylidae	4	21	25	0.16	0.0006*	**Under**
Megophryidae	1	6	7	0.14	0.0344	NSD
Arthroleptidae	4	26	30	0.13	6.19E-05*	**Under**
Mantellidae	0	48	48	0	1.05E-11*	**Under**
Ceratobatrachidae	0	3	3	0	0.04688	NA
Brevicipitidae	0	3	3	0	0.0456	NA
Pelodytidae	0	1	1	0	0.1676	NA
Hemisotidae	0	1	1	0	0.1652	NA
**Total Anura**	**449**	**486**	**935**			
***Caudata***						
Cryptobranchidae	3	0	3	1	0.9646	NA
Salamandridae	14	4	18	0.78	0.9947*	**Over**
Ambystomatidae	13	5	18	0.72	0.9809*	**Over**
Proteidae	1	1	2	0.50	0.5264	NA
Sirenidae	1	1	2	0.50	0.5191	NA
Plethodontidae	27	34	61	0.44	0.2647	NSD
Hynobiidae	0	9	9	0	0.0018*	**Under**
Amphiumidae	0	1	1	0	0.1683	NA
**Total Caudata**	**59**	**55**	**114**			
***Gymnophiona***						
Caeciliidae	0	5	5	0	0.0165*	**Under**
Ichthyophiidae	0	1	1	0	0.1624	NA

Prev. = Prevalence: *Bd* detected/Total sampled. * denotes a significant deviation from the expected level of infection in that family. ‘NSD’ = not significant. ‘NA’ denotes that insufficient species had been sampled in that family to detect a significant deviation from a random level of infection. Power-analyses using binomial tests indicated that with six species sampled in a Family, there was sufficient power to detect a deviation in either direction. ‘*Bd* not detected’ data have limitations, as indicated in Supplemental Information. Notes on taxonomic names used are provided in Supplemental Information. Detections in table updated as of January 2011.

We examined which families deviated from the expected level of infection given the overall background prevalence in our data set (508/1,055; 48%), and found broad taxonomic patterns (Table 2). Microhylidae, Arthroleptidae, and Mantellidae were under-infected anurans: these families are principally terrestrial, and many species are direct developers (no tadpoles), which would reduce their exposure to the aquatic infective stage of *Bd*. Furthermore, the majority of tadpoles of microhylids that exhibit indirect development lack keratinized mouthparts, the target of infection in larval anurans [17]. Although the Bufonidae were also under-infected, some of the most dramatic *Bd*-driven declines have been seen in bufonids, such as *Anaxyrus boreas* (formerly *Bufo)* in North America and *Atelopus* spp. in Central America. Caudates of the family Hynobiidae were also under-infected, perhaps due to endemicity in Asia, where *Bd* prevalence is low and presence is patchy. Over-infected families (Table 2) were quite diverse and included those with relatively localized to broad distributions, with both aquatic and terrestrial life histories, including the direct-developing Craugastoridae. These patterns may be used to generate testable hypotheses, yet we note that family-scale infection patterns also may reflect a sampling bias in our non-randomly collected data, especially for families with taxonomically or geographically restricted sampling efforts.

### *Bd* and declining amphibian populations

**Figure 2:**
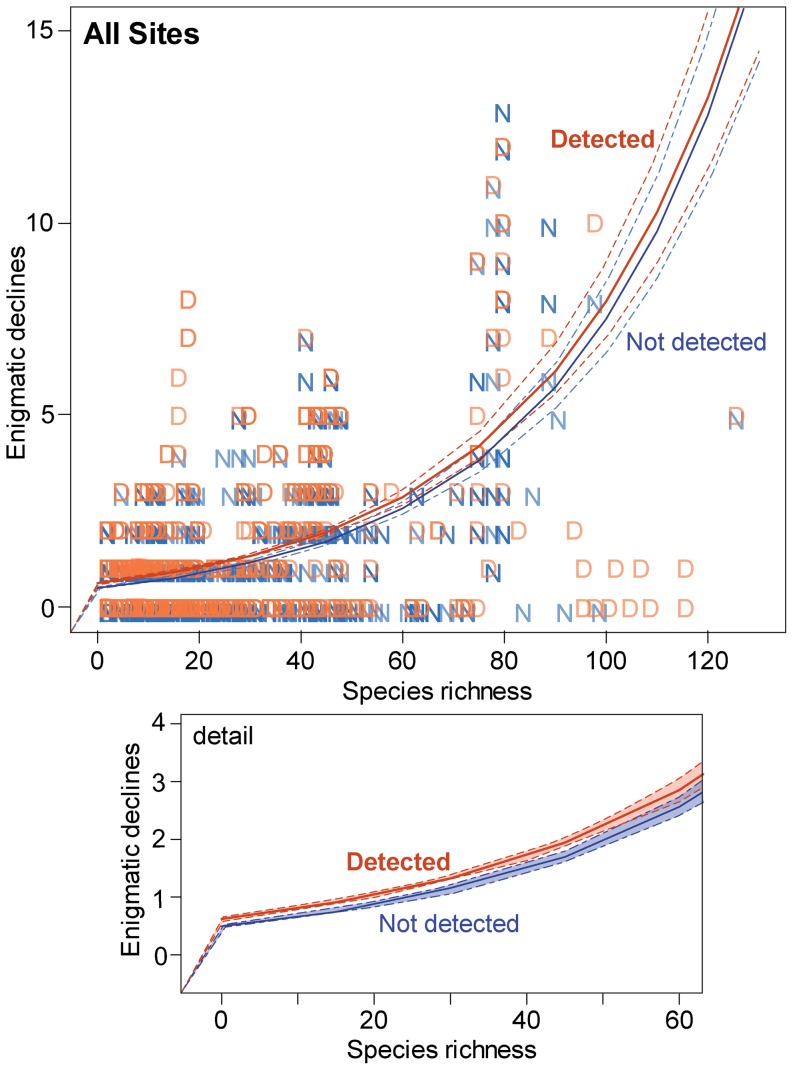
Global locations with enigmatic amphibian declines were positively associated with amphibian species richness (data from GAA [**7**]), and this relationship increased with the occurrence of the amphibian chytrid fungus, *Batrachochytrium dendrobatidis*, as modeled with logistic regression. Dashed lines indicate 95% confidence interval.

The Global Amphibian Assessment, GAA [7], reported that 47% of rapid amphibian species declines were ‘enigmatic’. While *Bd* has been suggested as a potential driver of these declines [40], data and analyses in support of this hypothesis have been lacking. Using the GAA data layer of world occurrences of enigmatic declines, we indirectly addressed this with data submitted to *Bd*-maps.net. We found that the average number of amphibian species experiencing enigmatic declines at a site was greater at sites where *Bd* was detected than where it was not detected (*p*<0.001; 3,961 sites used in analyses; Figure 2). The occurrence of enigmatic declines increased with increasing species richness, however the rate of increase of enigmatic decline occurrence with species richness did not differ between sites with and without *Bd* (*p* = 0.32; Figure 2). We repeated the analysis using data for the 3 families of amphibians [Bufonidae (908 sites), Hylidae (881 sites), and Ranidae (1,223 sites)] that dominated the database. As with the global data set, the average number of enigmatic declines was greater for sites with *Bd* than for sites without *Bd* for the Bufonidae and Ranidae, but the rate of increase in the relationship between the occurrence of enigmatic declines and species richness did not differ with *Bd* occurrence. The increase in enigmatic declines with richness was higher for sites without *Bd* for sites with hylids (*p*<0.001). Although such analyses may be biased, we report them because they may provide insights for follow-up studies. Due to these associations, we included both occurrences of enigmatic declines and species richness in our multivariate environmental models of *Bd* occurrence, below.

For species in which *Bd* infection was detected, 106 of 449 (24%) anurans and 12 of 60 (20%) caudates were listed in the three Threatened categories of the IUCN Red List (Critically Endangered, Endangered, Vulnerable; Table S1). It is important to note that these are counts of species that are recognized as being threatened without reference to what threats, according to the IUCN Threats Classification Scheme, are responsible for their classification. Animals under threat from *Bd* would be classified as 8.1 ‘Invasive non-native/alien species/diseases, 8.2 ‘Problematic native species/diseases’ or 8.4 ‘Problematic species/diseases of unknown origin’. Although *Bd* infection does not equate to it being a threat in every instance, upon inspection of the Red List, *Bd* may be under-reported as a concern, given the frequency of its occurrence as we report it here. For example, of the 12 caudates known to be infected with *Bd* that are currently ranked as threatened, *Bd* infection was listed as a threat for only one species, and disease, generally, was listed for a second species. To assess the relative impact of threats globally, an appropriate analysis would involve comparing the number of threatened species that are Red Listed because of a given process, versus the number of species that are exposed to that process. Currently, we are unable make such a comparison in the context of *Bd* because the only data we have are the infection status of a relatively small subset of the total number of amphibian species (1,240/6,996 species, 18%; species count from AmphibiaWeb.org, accessed 25 July 2012) and we lack conclusive data on symptoms of the disease chytridiomycosis for most of these species.

### *Bd* and environmental correlates: World and U.S.A. scales

**Figure 3:**
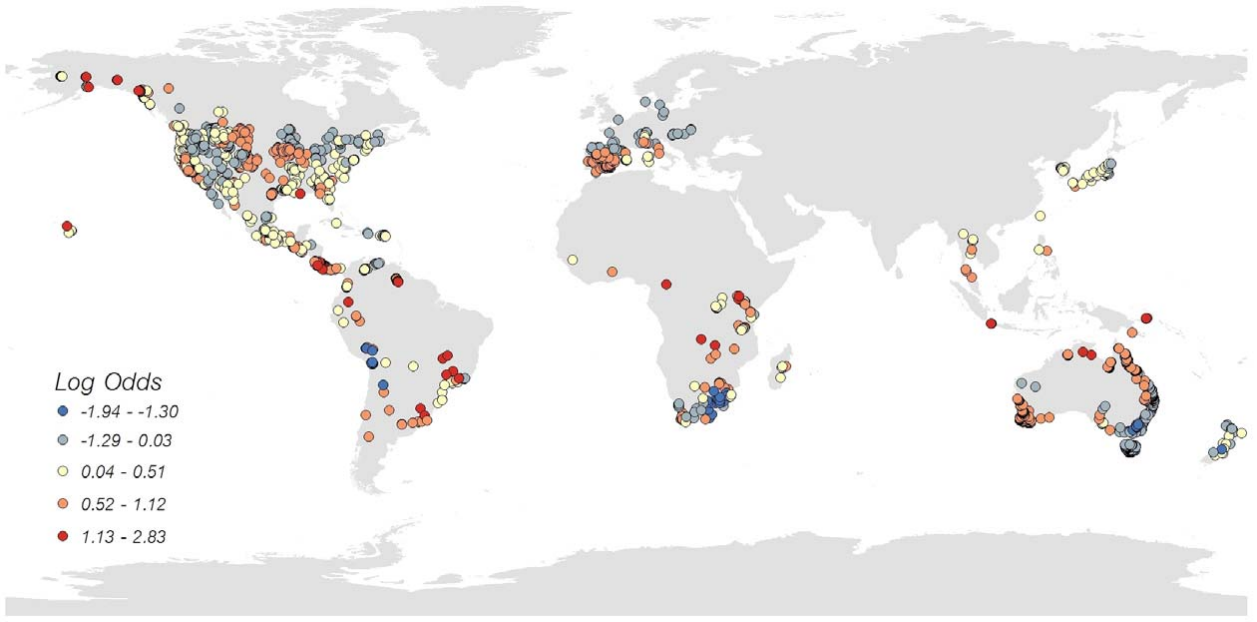
Odds of *Batrachochytrium dendrobatidis* (*Bd*) detection in amphibians derived from a regression model using data from *Bd*-maps.net from all species combined (data snapshot July 2010; N = 3,733 locations).

**Figure 4:**
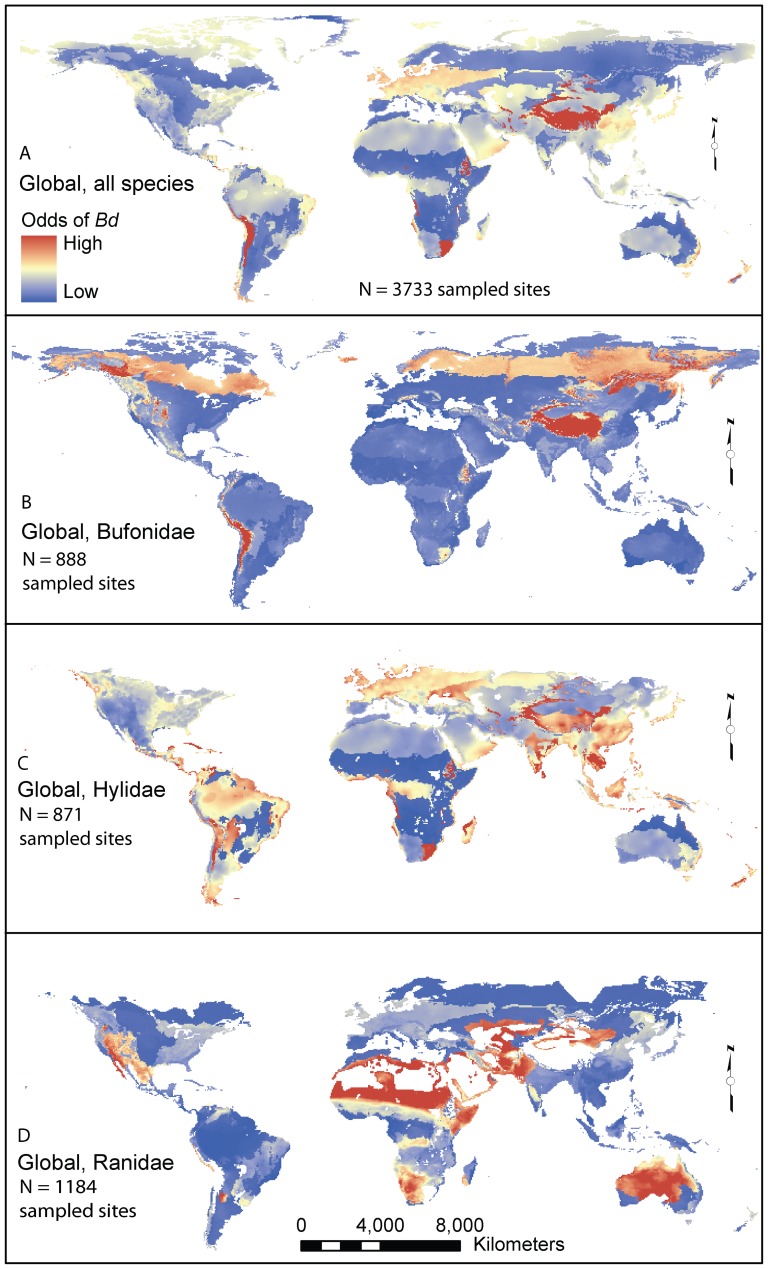
Extrapolated global maps of the odds of *Batrachochytrium dendrobatidis* (*Bd*) detection in amphibians derived from regression models. The global model using data from all species combined is shown (A), as well as subsets of the world data for species within the amphibian families Bufonidae (B), Hylidae (C), and Ranidae (D). Although these map projections depict the odds of *Bd* detection at the world scale, it should be noted that amphibians do not occur everywhere in the world, and in particular, the three amphibian families (B,C,D) are not native to all regions world-wide. Significant model parameters differed among models and included landscape-scale site attributes including climate metrics and biotic factors, such as biome or amphibian species richness (see text). Gaps in the mapped models are due to a lack of species richness data, or amphibian absence, for certain regions.

Our full model of odds of *Bd* occurrence by environmental parameters at sites using all world data (data snapshot from July 2010) included biomes, latitude, occurrence of enigmatic declines, temperature range, and average annual precipitation as significant explanatory variables (Table S2). Removing latitude from our analysis, which we considered warranted due to the overwhelming focus of *Bd-*surveillance in the northern hemisphere (Figure S1, Figure S2), resulted in only minor changes in the other coefficients, and slight changes to the order of biome entry into the model (Table 3; N = 3,733 sites used in analysis). After accounting for differences in odds of *Bd* detection between biomes, we found that the odds of *Bd* detection at discrete sites; 1) increased by 14% with each known increase in occurrence of enigmatic declines at a site; 2) decreased by 8.8% with each degree increase in temperature range at a site; and 3) decreased by 0.05% with each mm increase in average annual precipitation at a site (Table 3, Figure 3, Figure 4). Among biomes, sites in Montane Grasslands and Shrublands (N = 56 sites) had the highest odds of *Bd* occurrence. To better understand the inverse relationship of *Bd* presence with world temperature range values at sites, it is important to recognize that these temperature ranges represented the *differences* in average monthly values, and spanned 4.8 to 20.8^°^C. Large temperature ranges were found in: 1) Deserts and Xeric Shrublands; 2) Tropical and Subtropical Coniferous Forests; and 3) Temperate Coniferous Forests. Sites in montane areas of Mexico and Chile had moderately large temperature ranges. Sites with small temperature ranges in our analysis occurred in Tasmania, Italy, Denmark, Alaska, and South Africa.

**Table 3:** Significant parameters in the regression model using *Batrachochytrium dendrobatidis* (*Bd*) occurrence and data from wild-occurring amphibians having precise locations in the global *Bd* database (N = 3,733 locations).

Parameter	Coefficient	SE	P value
Intercept	1.186	0.262	––
Enigmatic Declines	0.141	0.028	<0.001
Temperature Range	−0.089	0.017	<0.001
Average Annual Precipitation	−0.0005	0.000	<0.001
Biome	––	––	<0.001
Tundra (included in intercept)	––	––	
Montane Grasslands & Shrublands	1.929	0.366	
Mediterranean Forest, Woodlands & Scrub	−0.598	0.126	
Tropical & Subtropical Dry Broadleaf Forests	−0.346	0.406	
Boreal Forests/Taiga	−0.649	0.340	
Mangroves	0.251	0.773	
Deserts & Xeric Shrublands	0.471	0.186	
Flooded Grasslands & Savannas	0.323	0.576	
Temperate Broadleaf & Mixed Forests	0.397	0.122	
Temperate Grasslands, Savannas & Shrublands	−0.346	0.172	
Temperate Coniferous Forests	0.229	0.124	
Tropical & Subtropical Grasslands	−0.582	0.198	
Tropical & Subtropical Moist Broadleaf Forests	0.224	0.172	
Tropical & Subtropical Coniferous Forests	0.503	0.278	

Individual biome coefficients represent additive shifts in the *Bd*-occurrence odds ratio, whereas other coefficients represent multiplicative changes in odds of detecting *Bd*. Logistic regression with likelihood ratio test statistic (Chi^2^) was used.

Family-scale analyses were first conducted using all data to explore potential *Bd*-associations between higher-order taxonomy (family) and geographic patterns. Our analyses included the three main amphibian families in our world sample (Ranidae, Bufonidae, Hylidae: 78% of sites; Table S3). In our Bufonidae model (N = 888 sites; Figure 4), the odds of *Bd* detection increased 44.7% with each 1,000-m increase in elevation, and were highest in Boreal Forest and Taiga, Temperate Coniferous Forests, Montane Grasslands and Shrublands, and Tropical and Subtropical Dry Broadleaf Forests. In Hylidae (N = 871 sites; Figure 4), the odds of detecting *Bd*: 1) increased by 10% with each incremental increase in occurrence of enigmatic declines; 2) increased by 1.9% with each degree increase in average annual temperature; and 3) decreased by 14.9% with each degree increase in average temperature range. The highest odds of *Bd* detection for Hylidae were in Montane Grasslands and Shrublands, Tropical and Subtropical Dry Broadleaf Forests, and Temperate Grasslands, Savannahs, and Shrublands. In Ranidae (N = 1,184 sites; Figure 4), the odds of *Bd* detection increased by 6.4% with each degree increase in average temperature range, decreased by 4% with each incremental increase in species richness, and decreased by 20% with each 1000-m increase in elevation. The highest odds of *Bd* detection in ranids occurred in two biomes, Tropical and Subtropical Grasslands, and Deserts and Xeric Shrubland. Several biomes were dropped from the ranid analysis due to data gaps.

Half of our compiled sites are located in the U.S.A., where we also were able to use environmental data with finer spatial resolution in our analyses. U.S.A. models of *Bd* occurrence were developed for all species combined, and for the three main families (83% of total sites sampled in U.S.A.), similar to the world-scale analyses. The full U.S.A. model with all taxa included (1,880 sites), showed an increase in odds of *Bd* detection with species richness, minimum temperature, an interaction between richness and minimum temperature, and biome. Due to the interaction term, interpretation of this model was more complex. For sites with species richness>15, the highest odds of *Bd* detection were at sites with average annual minimum temperatures<5°C. Sites with richness<10 species had roughly constant odds of *Bd* detection. For sites with average minimum temperature above 5°C, there was little change in odds of detection. *Bd* detection likelihood was greatest in Temperate Broadleaf and Mixed Forests. The U.S.A. Bufonidae model showed that the odds of *Bd* detection increased by 6.7% with each additional species at a site, and decreased 12.5% with each degree of average annual temperature. Two biomes were positively associated with bufonid *Bd* odds of detection: Deserts and Xeric Shrublands, and Temperate Coniferous Forests. The U.S.A. Hylidae model included only species richness and occurrence of enigmatic declines: when enigmatic declines = 1, there was an estimated 29% increase in odds of *Bd* detection when species richness increased by one. The map of this model was not informative due to the few enigmatic declines known for U.S.A. hylids. The U.S.A. Ranidae model included only biome. The odds of detecting *Bd* in U.S.A. ranids were highest in Desert and Xeric Shrublands, Temperate Broadleaf and Mixed Forests, and Temperate Coniferous Forests.

## Discussion

Preliminary analyses of data gathered over a four-year period support broad-scale differences in patterns of infection between amphibian families. These results provide insights to family-specific environmental traits associated with *Bd* infection. Downscaled environmental hypotheses and assessments are warranted to gauge relationships that may help explain infection patterns at local to regional scales.

Largely due to efforts of the *Bd* Mapping community, we show that the spatial distribution of *Bd* is highly heterogeneous. It appears that a proximal driver of this heterogeneity is the rapid, continuing spread of *Bd* into naïve regions, and it appears that *Bd* has not yet reached a global equilibrium. Within this increasing pattern of spread, we identified nested variables that may influence the ecology and epidemiology of *Bd* following potential introduction into a region; these variables contain taxonomic (see under- and over-infected families), environmental (e.g., temperature range and precipitation at a site), and community-level (e.g., species richness) determinants. While models that combine spatial-spread dynamics with stationary site-specific equilibria are required for understanding and predicting the current and further impact of *Bd*, model clarity will only be achieved when we have a better understanding of the fine-scale epidemiological processes that reflect amphibian diversity, habitat, and community-level interactions.

*Bd* relationships with temperature are supported by experimental work showing that *Bd* does not grow well at temperatures≥28°C [13], [41], field work describing increased infection prevalence and mortality under cooler conditions [30], [31], [42], [43], and other *Bd*-occurrence models, such as the downscaled model using the software Maxent which was applied to data from Australia [44]. Although altitude was not a significant predictor in the current analysis (Figure S3), the highest odds of detecting *Bd* were in the biome Montane Grasslands and Shrublands (Figure S4). This suggests that altitude *per se* is not a globally-uniform predictor of *Bd*-presence, and that the risk of chytridiomycosis to montane-associated species may need to be assessed using regional, spatially restricted data sets. Species-specific and site-specific approaches may yield a better understanding of the dynamics of *Bd*-occurrence among such montane amphibians.

Our analyses support the associations of *Bd* with global-level temperature metrics, supporting hypotheses that the future occurrence of chytridiomycosis will respond to changes in global climate conditions [32], [45]. However, there appears to be little support at this time that climate change is associated with chytridiomycosis, within at least parts of its range where this has been studied [35]. Our global data and the model created for Australia [44] suggest that both temperature range and precipitation may be of particular importance for future investigations. Our model predicts an almost 9% decrease in the odds of *Bd* detection with each degree-C increase in temperature range at a site, with Hylidae contributing significantly to this result and Ranidae having an opposing tendency. This aspect of our study will be particularly informative for predicting the effects of warmer or more variable weather patterns on the worldwide incidence of chytridiomycosis. A caveat here is that such broad-brush analyses inevitably miss fine-scale relationships between amphibians, their microhabitats, and the little-understood relationship between temperature and the ectothermic immune-response: these factors are all likely key to determining the local outcome of infection [46].

Our finding of increased occurrence of *Bd* with species richness supports the hypothesis that greater complexity of amphibian communities may amplify the transmission of *Bd*. Theoretical community epidemiology shows that when between-species transmission rates (β_between_) increase, both the prevalence of infection and the proportion of time that hosts are infected increases [47]. Where heterogeneity exists between species in their transmission potential, then even poor transmitters of the pathogen (β_within_ → 0) may suffer repeated exposures in multi-host systems. Additionally, the number of secondary infections caused by a single infected individual (*R*_0_) may increase in relation to increasing species (reservoir) density [48]. For amphibians, it is known that mortality as a consequence of exposure to *Bd* is both dose-dependent [15], [49], [50] and species-specific [17], [51]; some species succumb to very low levels of infection while others never exhibit clinical signs of disease [14], [52]. It is possible that the strong positive relationship between species richness and enigmatic decline detailed in Figure 2 reflects the epidemic ‘forcing’ of infection in heterogeneous speciose systems following the introduction of *Bd*. However, the concurrence of rare species with restricted geographic ranges in areas with high species richness also may contribute to this pattern, if those rare species are also more likely to be tied to Red List criteria for enigmatic declines. Nevertheless, the risk that chytridiomycosis poses to a single amphibian species may be strongly tied to location, as well as being species-specific, with the epidemiological outcome dependent on a potentially very large number of inter-related biotic and abiotic variables. Mechanistic multi-species epidemiological models incorporating taxonomic selectivity in risk alongside community composition and local-scale environmental drivers will provide the key to unlocking the site-specific dynamics of *Bd* and chytridiomycosis, and thus assessing the risk to particular species and amphibian communities. However, the spatial modeling of an infectious disease at this level of complexity, with such broad host-numbers and parameter values, has never been attempted and will present a considerable challenge to mathematical epidemiologists. To aid this process, the modeling of amphibian-*Bd* dynamics in simple systems with few hosts, aided by controlled laboratory experiments for accurate parameter assessment, will prove highly valuable [52], [53].

The impact of the *Bd* mapping project is still unfolding. First, this project demonstrates the value of social networking among scientists, with data compiled from hundreds of contributors who have assessed the occurrence of *Bd* at over 4,000 sites worldwide. *Bd*-maps.net allows an ongoing synergy among researchers and managers as global data are communicated or exported for further uses. Second, the project has led to a number of new studies and programs. As a consequence of the project, biologists have taken the initiative to: 1) initiate *Bd* monitoring and assessment in their region and to leverage funding for projects with expanded scope (e.g., the pan-European chytridiomycosis risk assessment RACE; www.bd-maps.eu); 2) use *Bd*-maps.net to direct work toward spatial and taxonomic gaps (e.g., U.S.A. states, Southeast Asia; e.g., *Bd* surveillance papers published in the Amphibian Diseases section of *Herpetological Review*); 3) to instigate disease mitigation programs in focal systems (e.g., Mallorca and *Alytes muletensis*; [54]); 4) include *Bd*-occurrence data in aquatic invasive species alerts for land management (e.g., for water-draw decisions for western US wildfire management; Figure S5); and 5) to facilitate novel investigations into the complex ecological patterns associated with *Bd* infection [55].

To enhance the utility of *Bd*-maps.net for the global science and management community, dedicated curation and community deposition of data are needed. Currently, part-time efforts of individuals at two institutions, the U.S. Forest Service, Pacific Northwest Research Station, Oregon, U.S.A., and Imperial College, London, U.K., allow limited technical assistance for data contributors. Broader institutional support from the global community could tie data reporting to permit processes and publications for broader community participation and uses. For example, infection trend monitoring could easily be conducted for local jurisdictions, would be more effective with surveillance reporting tied to existing permitting processes, and could lead to insights for understanding transmission vectors or infectivity correlates. Molecular typing of *Bd* isolates [16] is continuing and is planned to be integrated with mapping data to understand the global spread of infection within a population genomics framework [56]. The addition of molecular data to *Bd*-maps.net, alongside rapid next-generation genotyping techniques, may aid in identifying the major sources and sinks of infection, and the dominant global vectors of *Bd*. We are applying our lessons-learned in the development of B*d*-maps to the current development of a global Ranavirus Reporting System [57], [58]. This developing system includes data fields to assess data certainty and potential bias. Understanding of the vectors for both *Bd* and Ranaviruses will be key for regional biosecurity, with subsequent emphasis on management of the human-mediated transmission pathways. An expected outcome is the need for increased transparency of disease transmission pathways in amphibian trade within the context of the OIE legislation, as well as a call for all countries contributing to the OIE infrastructure to contribute to the detection and containment of novel wildlife diseases. These approaches are synergistic with those proposed to contain the emergence of newly emerging human diseases, and while the challenges are great, the imperative to curb the spread of these globally-devastating infections has never been greater.

## Materials and Methods

### Data compilation

*Bd* location data were compiled from published literature and unpublished studies (Table S1). We defined ‘site’ as a geographically distinct location having specific coordinates and/or locality description. A ‘record’ was a database entry for a single species for a single year, at a particular site. A given site may have a number of records for different species or years, some for which *Bd* was found, others for which it was not found. A ‘country centroid’ is a point designation for a type of site that placed sampled species in the country of origin, where finer-scale location data were not available. Country centroid data were compiled generally from early years of *Bd* surveillance before Global Positioning Systems were frequently used to determine site coordinates.

Site-level data were used in analyses, and if both positive and negative *Bd* records were available for a site, the site was designated *Bd*-positive. Analyses did not include data from captive animals, vague locations, or country centroids. For analyses, a snapshot of site-level data was taken in July 2010, including all data compiled in our database up to that time. Analyses were conducted using a global grid spatial resolution of 0.5 degrees latitude and longitude, which was the finest scale available for the global climate data. Finer-scale habitat patterns will not be apparent from our analyses, such as those with more restricted temperature regimes. Parameters used in our models are listed in Table S3.

To provide a more updated summary of *Bd* occurrence (Table 1, Table 2, Table S1), we report tallies of *Bd*-positive and *Bd*-negative data compiled up through a data snapshot taken on 30 January 2011. These summaries were used for the over/under-infection randomization analysis. Our data are available via a world *Bd* mapping database and interactive web application that we developed for the *Bd* mapping project, which has merged and extended pre-existing data compilation efforts (http://www.Bd-maps.net). The *Bd*-maps.net website facilitates automatic surveillance by country or species at several spatial scales which can be user-determined by an interactive mapping tool using Google Maps technology. Hence, real-time counts by country and species are available online, and recent data snapshots of these tallies were taken on 25 July 2012 for this paper. Some sensitive data were not released for precise mapping, and hence our hierarchical mapping method enables their mapping at world scale but not at finer scales. However, sensitive data were included in our data snapshots for both analyses and more recent summaries. For the purposes of this paper, a data snapshot was undertaken for our analyses (June 2010), and we have provided additional time-sensitive snapshots for more updated information in Figure 1 (August 2012) and Table S1 (January 2011). Data updates and analyses are time-intensive procedures to ensure duplicate records are screened from our counts, accounting for different snapshot dates reported here. The most recent (July 2012) counts of *Bd*-infected countries and species is the only realtime data summary available at this time.

Caution is needed due to several potential data constraints when interpreting our maps and analyses. Limited quality assurance was conducted, such that if inaccurate methods were used or results reported, those data may not have been screened out of this compilation (e.g., false positives and negatives may be included in the data). *Bd* prevalence in a population may be low, such that a small sample size of individuals screened for *Bd* may not detect it, and consequently could yield a false negative report (‘no detection’ does not indicate absence of *Bd*; see [39]). This could be a particular concern for data collected before 2008, when the recommendation for sample sizes>59 be collected to assess *Bd* occurrence when prevalence is low [39]. In addition, it is possible that negative data are not consistently reported, especially from early exploratory studies or due to the challenge of publishing negative data, and hence negative data may be under-represented in our compilation. To conduct adequate quality assurance of the global data set to reduce potential biases, individual studies would need to be screened, which would require each data source to be revisited and assessed. Our aggregation of data at the 0.5 degree latitude and longitude scale likely reduces these biases by compiling data among different sampling events, including separate studies (and their methods), researchers, species, seasons, and years. Finally, our world map of *Bd* detections (Figure 1) includes samples taken from living or dead animals—appearing healthy or symptomatic, native and introduced animals, and wild and captive animals. We do not map the disease chytridiomycosis wherein animals showed symptoms of the disease. This last point should be emphasized; we report *Bd* occurrence and hence the threat of disease and potential taxonomic or geographic sources of disease transmission, but we have not distinguished locations with mortality attributed to the disease and our maps do not represent declining amphibian populations. In addition, our logistic regression models should not be used for direct prediction, and coefficients from the fitted models should only be interpreted in terms of relative (not absolute) contribution to the odds of *Bd* detection. Because sites were not randomly selected, the inference of each model is restricted to those sites used in the analysis. However, with 3,733 sites used in our full model, we feel there is useful predictive capacity of those data to be used as hypotheses for future investigation and later downscaled analyses, especially for the better-sampled regions of the world and more heavily-sampled amphibian families.

During modeling, all of our 3,961 *Bd* sites during that data snapshot did not fall cleanly within available environmental coverages, and hence some were not included in regression analyses. For example, the biome coverage did not overlay cleanly with the *Bd* site data. For 227 sites, site location did not correspond to an environmental coverage after the overlay was conducted, and these were eliminated from the model. These sites were distributed around the world and often were near coastlines, suggesting the coastal accuracy of the landscape-scale environmental boundaries was poor; hence, these sites had very low elevations. However, there were often neighboring locations that were sorted into a biome, so locations with clusters of samples were not generally lost from analyses. We also deleted the ‘lake’ biome from analyses (1 site) because lentic habitats within other biome types were not distinguished.

### Statistical Analysis

Using logistic regression (programmed in R software [59]), we examined associations between the *Bd* occurrence at a site (world scale: all data and three well-sampled families separately – Bufonidae, Hylidae, Ranidae) and: 1) latitude; 2) elevation [60]; 3) biome [61]; 4) amphibian species richness [7]; and 5) global temperature and precipitation metrics ([62]; Climatic Research Unit, University of East Anglia, Norwich, UK; www.cru.uea.ac.uk) (Table S2). These parameters were chosen to represent world-scale ecological factors that may be correlated with *Bd*. Our intent was to use our robust data set to address existing hypotheses of *Bd*-occurrence correlations, and to contribute to new ecological research and surveillance directions. We did not include other factors that may better represent a human footprint signal from modern-day disturbances (e.g., proximity to roads, cities, agricultural areas) largely because these data layers are inconsistent at the world scale.

Additionally, because the United States was a relatively well-sampled large-scale region, we conducted similar analyses for all U.S.A. data and well-sampled families in the U.S.A. (Bufonidae, Hylidae, Ranidae). More precise temperature and precipitation data were used in the U.S.A. analyses (Parameter-elevation Regressions on Independent Slopes Model [PRISM], C. Daley, PRISM Climate Group, http://prism.oregonstate.edu/). Altogether, eight *Bd*-occurrence models were generated: world scale, all data and data from three families; U.S.A. scale, all data and data from three families.

Logistic regression also was used to examine the relationship between locations with enigmatic population declines [7] and both *Bd* detection and species richness (decline and richness data sources; [7]) detailed in Table S3. Again, we conducted eight analyses: world scale with all world data, and world data from three families; U.S.A. scale with all U.S.A. data, and U.S.A. data from three families.

Randomisation tests were used to determine whether each sampled amphibian family was over- or under-infected, compared to what we would expect by chance given an overall prevalence of 508/1055 species (using the January 2011 data snapshot reflected in Table 1, Table 2, and Table S1). Ten thousand unconstrained randomizations were performed using a computer program written in the statistical language R [59]; infection data was randomised across all species in the data set, and the number of species in the focal family that were randomly assigned as infected was counted. The null distribution generated was then compared to the observed number of infected species within the focal family, and the null hypothesis (that infection was random) was rejected if the observed value lay in the 2.5% for either tail of the null distribution, and statistical power was sufficient (no. species>6). Power-analyses using binomial tests indicated that with six species sampled in a family, there was sufficient power to detect a deviation in either direction.

## Supporting Information

Figure S1 **Occurrence of** ***Batrachochytrium dendrobatidis*** **(** ***Bd*** **) at sites by latitude.** The high number of sample sites in the Northern Hemisphere, particularly the United States and Spain, gives a pronounced skew to this distribution.
(TIF)

Figure S2 **Taxonomic patterns of** ***Batrachochytrium dendrobatidis*** **(** ***Bd*** **) occurrence by latitude.** The extraordinary peak in species richness in the 8°N range highlights the overlap of many families of amphibians in Central America and also the intensive sampling represented by Lips et al. (2003, 2006). Of additional interest are the very broad latitudinal ranges of the families Hylidae, Bufonidae, and Ranidae. This range explains, in part, why they were so widely sampled, and *Bd* was so widely detected among them, and therefore also why they were chosen as the three families we modeled. Note also how the number of families thins out at very high and very low latitudes.
(TIF)

Figure S3 **Occurrence of** ***Batrachochytrium dendrobatidis*** **at sites by elevation, with annotation by ecoregion of site.** Vertical black lines indicate the range of elevations covered by a particular ecoregion. For the most part, only ecoregions or aggregates of ecoregions with more than 20 sites represented are shown in the annotation. Low elevation sites (below 1000 m) were broadly distributed across the world, and across a wide variety of ecoregions. ‘bsl’  =  ‘below sea level’, for a site in the Coachella Valley, California, U.S.A. Country abbreviations: AR  =  Argentina, CA  =  Canada, CH  =  Switzerland, CL  =  Chile, ES  =  Spain, FR  =  France, IT  =  Italy, MX  =  Mexico, PE  =  Peru, US  =  United States.
(TIF)

Figure S4 **Occurrence of** ***Batrachochytrium dendrobatidis*** **(** ***Bd*** **) at sites among 15 world biomes.** ‘Lake’ (98) was dropped from our analyses, as there was only one site so classified owing to the coarse scale of the bioregional data relative to our site locations. Among biomes, the highest odds of detecting *Bd* were in Montane Grasslands and Shrublands (Biome 10, reported from Australia, New Zealand, South Africa, and the Chilean and Peruvian Andes), and the lowest odds of detection were in Mediterranean Forests, Woodlands, and Scrub (Biome 12, based on sites in Australia, Spain, Italy, South Africa, and California (U.S.A.), and in Tundra (Biome 11, based on sites in the interior and Kenai Peninsula of Alaska, U.S.A.).
(TIF)

Figure S5 ***Batrachochytrium dendrobatidis*** **(** ***Bd*** **) occurrence by 6^th^-field Hydrologic Units (HU; watershed) for the U.S.A.** Natural resource planning and management decisions often occur by watershed in the US. For example, disease disinfection protocols often stipulate disinfection between drainages, and 6^th^-field watershed delineations are used for water draw decisions during wildfire season in parts of the US. Aquatic invasive species including *Bd* are mapped by watershed to inform decision-makers about risk of transmission during water draw decisions. Map reflects data as of January 2011.
(TIF)

Table S1 ***Bd*** **detections by species, with references and countries of detection.** Family-level taxonomy is shown according to Frost et al. (2006), Grant et al. (2006), Frost (2009), and Hedges et al. (2008). Species name is shown as given in the report of the *Bd* occurrence. Where assignments to genus or species have changed since the species was reported as being found with *Bd*, the older name is given in parentheses. For further information on taxonomy, see Taxonomic Notes. The abbreviation (cap.) after a species name indicates an infected captive animal or animals. Status is Conservation Status according to the IUCN Red List (IUCN 2010). Categories are defined as: EX  =  extinct; EW  =  Extinct in the Wild; CR  =  Critically Endangered; EN  =  Endangered; VU  =  Vulnerable (the previous three categories are considered Threatened by the IUCN); NT  =  Near Threatened; LC  =  Least Concern; DD  =  Data Deficient; NE  =  Not Evaluated (newly discovered or newly recognized species). Last updated from published literature in March 2011; references are given in Supplemental Information text, File S1.
(DOCX)

Table S2 **Parameters used in regression analyses.** Numbers in parentheses after biomes indicate number of sites in the full analysis (N  =  3733 total sites). Sites with no biome assigned (n  =  227) were dropped from the analysis. Lake biome (N  =  1 site) was also dropped due to inconsistency of lentic habitat designation.
(DOCX)

Table S3 **Significant parameters in three family-scale logistic regression models using** ***Batrachochytrium dendrobatidis*** **(** ***Bd*** **) occurrence data from wild-occurring amphibians having exact and approximate locations in the global** ***Bd*** **database.** Individual biome coefficients represent additive shifts in the *Bd*-occurrence odds ratio, whereas other coefficients represent multiplicative changes in odds of detecting *Bd*. Logistic regression with likelihood ratio test statistic (Chi^2^) was used.
(DOCX)

File S1 **Supplemental Information: Taxonomic Notes; References for Supplemental Material.**
(DOCX)

## References

[1] Ferguson NM, Donnelly CA, Anderson RM (2001) Transmission intensity and impact of control policies on the foot and mouth epidemic in Great Britain. Nature 413: 542–548. 11586365 10.1038/35097116

[2] Berger L, Speare R, Daszak P, Green DE, Cunningham AA, et al (1998) Chytridiomycosis causes amphibian mortality associated with population declines in the rain forests of Australia and Central America. Proc Natl Acad Sci U S A 95: 9031–9036. 9671799 10.1073/pnas.95.15.9031 PMC21197

[3] Johnson PTJ (2006) Amphibian diversity: Decimation by disease. Proc Natl Acad Sci U S A 103: 3011–3012. 16492751 10.1073/pnas.0600293103 PMC1413947

[4] Gascon C (2007) Amphibian Conservation Action Plan. IUCN/SSC Amphibian Specialist Group, Gland, Switzerland and Cambridge, UK.

[5] McCallum ML (2007) Amphibian decline or extinction? Current declines dwarf background extinction rate. J Herpetol 41: 483–491.

[6] Skerratt LF, Berger L, Speare R, Cashins S, McDonald KR, et al (2007) Spread of chytridiomycosis has caused the rapid global decline and extinction of frogs. EcoHealth 4: 125–134.

[7] Stuart SN, Chanson JS, Cox NA, Young BE, Rodrigues AS, et al (2004) Status and trends of amphibian declines and extinctions worldwide. Science 306: 1783–1786. 15486254 10.1126/science.1103538

[8] Wake DB, Vredenburg VT (2008) Are we in the midst of the sixth mass extinction? A view from the world of amphibians. Proc Natl Acad Sci U S A 105: 11466–11473. 18695221 10.1073/pnas.0801921105 PMC2556420

[9] Colón-Gaud C, Whiles MR, Kilham SS, Lips KR, Pringle CM, et al (2009) Assessing ecological responses to catastrophic amphibian declines: patterns of macroinvertebrate production and food web structure in upland Panamanian streams. imnol Oceanogr 54: 331–343.

[10] Colón-Gaud C, Whiles MR, Kilham SS, Lips KR, Pringle CM, et al (2010) Macroinvertebrate community responses to catastrophic amphibian declines in neotropical streams. J North Amer Benthol Soc 2: 1185–1198.

[11] Connelly S, Pringle CM, Kilham S, Whiles MR, Lips KR, et al (2011) Do tadpoles affect leaf decomposition in neotropical streams? Freshwater Biol 56: 1863–1875.

[12] Fisher MC, Henk DA, Briggs C, Brownstein JS, Madoff L, et al (2012) Emerging fungal threats to animal, plant and ecosystem health. Nature 484: 186–194. 22498624 10.1038/nature10947 PMC3821985

[13] Longcore JE, Pessier AP, Nichols DK (1999) *Batrachochytrium dendrobatidis* gen et sp nov, a chytrid pathogenic to amphibians. Mycologia 91: 219–227.

[14] Fisher MC, Garner T, Walker J (2009) Global emergence of *Batrachochytrium dendrobatidis* and amphibian chytridiomycosis in space, time and host. Ann Rev Microbiol 63: 291–310. 19575560 10.1146/annurev.micro.091208.073435

[15] Garner TW, Walker S, Bosch J, Leech S, Rowcliffe M, et al (2009) Life history trade-offs influence mortality associated with the amphibian pathogen *Batrachochytrium dendrobatidis* . Oikos 118: 783–791.

[16] Farrer RA, Weinert LA, Bielby J, Garner TWJ, Balloux F, et al (2011) Multiple emergences of genetically diverse amphibian-infecting chytrids include a globalized hypervirulent recombinant lineage. Proc Natl Acad Sci U S A doi:10.1073/pnas.1111915108. 10.1073/pnas.1111915108 PMC3219125 22065772

[17] Reeder NMM, Pessier AP, Vredenburg VT (2012) A reservoir species for the emerging amphibian pathogen *Batrachochytrium dendrobatidis* thrives in a landscape decimated by disease. PLoS ONE 7(3): e33567 doi:10.1371/journal.pone.0033567. 22428071 10.1371/journal.pone.0033567 PMC3299797

[18] Daszak P, Strieby A, Cunningham AA, Longcore JE, Brown CC, et al (2004) Experimental evidence that the bullfrog (*Rana catesbeiana*) is a potential carrier of chytridiomycosis, an emerging fungal disease of amphibians. Herpetol J 14: 201–207.

[19] Garner TW, Perkins M, Govindarajulu P, Seglie D, Walker S, et al (2006) The emerging amphibian pathogen *Batrachochytrium dendrobatidis* globally infects introduced populations of the North American bullfrog, *Rana catesbeiana* . Biol Lett 2: 455–459. 17148429 10.1098/rsbl.2006.0494 PMC1686185

[20] Fisher MC, Garner TWJ (2007) The relationship between the introduction of *Batrachochytrium dendrobatidis*, the international trade in amphibians and introduced amphibian species. Fungal Biol Rev 21: 2–9.

[21] Picco AM, Collins JP (2008) Amphibian commerce as a likely source of pathogen pollution. Conserv Biol 22: 1582–1589. 18717688 10.1111/j.1523-1739.2008.01025.x

[22] Garmyn A, Van Rooij P, Pasmans F, Hellebuyck T, Van Den Broeck W, et al (2012) Waterfowl: Potential environmental reservoirs of the chytrid fungus *Batrachochytrium dendrobatidis* . PLoS ONE 7(4): e35038 doi:10.1371/journal.pone.0035038. 22514705 10.1371/journal.pone.0035038 PMC3325947

[23] El Mouden EH, Slimani T, Donaire D, Fernández-Beaskoetxea S, Fisher MC, et al (2011) First record of the chytrid fungus *Batrachochytrium dendrobatidis* in North Africa. Herpetol Rev 42: 71–75.

[24] Kirshtein JD, Anderson CW, Wood JS, Longcore JE, Voytek MA (2007) Quantitative PCR detection of *Batrachochytrium dendrobatidis* DNA from sediments and water. Dis Aquat Org 77: 11–15. 17933393 10.3354/dao01831

[25] OIE (2008) Report of the meeting of the OIE Aquatic animal health standards commission, Paris, 3–7 March 2008. 76th General Session

[26] Swei A, Rowley JJL, Rödder D, Diesmos MLL, Diesmos AC, et al (2011) Is chytridiomycosis an emerging infectious disease in Asia? PLoS ONE 6(8): e23179 doi:10.1371/journal.pone.0023179. 21887238 10.1371/journal.pone.0023179 PMC3156717

[27] Olson DH, Ronnenberg KL (2008) http://www.parcplace.org/bdmap2008update.html.

[28] Aanensen DM (2009) The development and use of bioinformatic web applications for Infectious Disease Microbiology. Thesis. https://unicorn.lib.ic.ac.uk/uhtbin/ckey/825195.

[29] Woodhams DC, Alford RA (2005) Ecology of chytridiomycosis in rainforest stream frog assemblages of tropical Queensland. Conserv Biol 19: 1449–1459.

[30] Berger L, Speare R, Hines H, Marantelli G, Hyatt AD, et al (2004) Effect of season and temperature on mortality in amphibians due to chytridiomycosis. Aust Vet J 82: 31–36. 15354853 10.1111/j.1751-0813.2004.tb11137.x

[31] Ron S (2005) Predicting the distribution of the amphibian pathogen *Batrachochytrium dendrobatidis* in the New World. Biotropica 37: 209–221.

[32] Bosch J, Carrascal LM, Duran L, Walker S, Fisher MC (2007) Climate change and outbreaks of amphibian chytridiomycosis in a montane area of Central Spain; is there a link? Proc R Soc Lond [Biol] 274: 253–260. 10.1098/rspb.2006.3713 PMC1685858 17148254

[33] Kriger KM, Hero JM (2007) Large-scale seasonal variation in the prevalence and severity of chytridiomycosis. J Zool 271: 352–359.

[34] Lips KR, Brem F, Brenes R, Reeve JD, Alford RA, et al (2006) Emerging infectious disease and the loss of biodiversity in a Neotropical amphibian community. Proc Natl Acad Sci U S A 103: 3165–3170. 16481617 10.1073/pnas.0506889103 PMC1413869

[35] Lips KR, Diffendorfer J, Mendelson JR III, Sears MW (2008) Riding the wave: Reconciling the roles of disease and climate change in amphibian declines. PLoS Biol 6: 441–454. 10.1371/journal.pbio.0060072 PMC2270328 18366257

[36] Muths E, Pilliod DS, Livo LJ (2008) Distribution and environmental limitations of an amphibian pathogen in the Rocky Mountains, USA. Biol Cons 141: 1484–1492.

[37] Murray KA, Skerratt LF, Speare R, McCallum H (2009) Impact and dynamics of disease in species threatened by the amphibian chytrid fungus, *Batrachochytrium dendrobatidis* . Conserv Biol 23: 1242–1252. 19774709 10.1111/j.1523-1739.2009.01211.x

[38] Aanensen DM, Huntley DM, Feil EJ, al-Own F, Spratt BG (2009) EpiCollect: Linking smartphones to web applications for epidemiology, ecology and community data collection. PLos ONE 4(9): e6968 doi:10.1371/journal.pone.0006968. 19756138 10.1371/journal.pone.0006968 PMC2735776

[39] Skerratt LF, Berger L, Hines HB, McDonald KR, Mendez D, et al (2008) Survey protocol for detecting chytridiomycosis in all Australian frog populations. Dis Aquat Org 80: 85–94. 18717061 10.3354/dao01923

[40] Bielby J, Cooper N, Cunningham AA, Garner TW, Purvis A (2008) Predicting susceptibility to future declines in the world's frogs. Conserv Lett 1: 82–90.

[41] Piotrowski JS, Annis SL, Longcore JE (2004) Physiology of *Batrachochytrium dendrobatidis*, a chytrid pathogen of amphibians. Mycologia 96: 9–15. 21148822

[42] Retallick RWR, McCallum H, Speare R (2004) Endemic infection of the amphibian chytrid fungus in a frog community post-decline. PloS Biol 2: 1965–1971. 10.1371/journal.pbio.0020351 PMC521176 15502873

[43] Forrest MJ, Schlaepfer MA (2011) Nothing a hot bath won’t cure: Infection rates of amphibian chytrid fungus correlate negatively with water temperature under natural field settings. PLoS ONE 6(2): e28444 doi:10.1371/journal.pone.0028444. 22205950 10.1371/journal.pone.0028444 PMC3244395

[44] Murray KA, Retallick RWR, Puschendorf R, Skerratt LF, Rosauer D, et al (2011) Assessing spatial patterns of disease risk to biodiversity: implications for the management of the amphibian pathogen, *Batrachochytrium dendrobatidis* . J Anim Ecol 48: 163–173.

[45] Pounds AJ, Bustamante MR, Coloma LA, Consuegra JA, Fogden MPL, et al (2006) Widespread amphibian extinctions from epidemic disease driven by global warming. Nature 439: 161–167. 16407945 10.1038/nature04246

[46] Lovich R, Ryan MJ, Pessier A, Claypool B (2008) Infection with the fungus *Batrachochytrium dendrobatidis* in a non-native *Lithobates berlandieri* below sea level in the Coachella Valley, California, USA. Herpetol Rev 39.

[47] Fenton A, Pedersen AB (2005) Community epidemiology framework for classifying disease threats. Emerg Infect Dis 11: 1815–1821. 16485464 10.3201/eid1112.050306 PMC3367628

[48] de Castro F, Bolker B (2005) Mechanisms of disease-induced extinction. Ecol Lett 8: 117–126.

[49] Vredenburg VT, Knapp R, Tunstall T, Briggs CJ (2010) Dynamics of an emerging disease drive large-scale amphibian population extinctions. Proc Natl Acad Sci U S A 107: 9689–9694. 20457913 10.1073/pnas.0914111107 PMC2906868

[50] Kinney VC, Heemeyer JL, Pessier AP, Lannoo MJ (2011) Seasonal pattern of *Batrachochytrium dendrobatidis* infection and mortality in *Lithobates areolatus*: Affirmation of Vredenburg's ‘10,000 Zoospore Rule’. PLoS ONE 6: e16708. 21423745 10.1371/journal.pone.0016708 PMC3053364

[51] Woodhams DC, Ardipradja K, Alford RA, Marantelli G, Reinert LK, et al (2007) Resistance to chytridiomycosis varies among amphibian species and is correlated with skin peptide defenses. Anim Conserv 10: 409–417.

[52] Briggs CJ, Vredenburg VT, Knapp RA, Rachowicz LJ (2005) Investigating the population-level effects of chytridiomycosis: An emerging infectious disease of amphibians. Ecology 86: 3149–3159.

[53] Mitchell KM, Churcher TS, Garner TWG, Fisher MC (2008) Persistence of the emerging pathogen *Batrachochytrium dendrobatidis* outside the amphibian host greatly increases the probability of host extinction. Proc R Soc [Biol] 275: 329–334. 10.1098/rspb.2007.1356 PMC2593721 18048287

[54] Lubick N (2010) Emergency medicine for frogs. Nature 465, 680–681. 10.1038/465680a 20535176

[55] Bancroft B, Han B, Searle C, Michael L, Olson DH, et al (2011) Environmental and biological correlates of species susceptibility to *Batrachochytrium dendrobatidis* in amphibians. Biol Conserv 20: 1911–1920.

[56] James TY, Litvintseva A, Vilgalys R, Morgan JA, Taylor JW, et al (2009) Rapid global expansion of the fungal disease chytridiomycosis into declining and healthy amphibian populations. PLoS Pathogens 5: e1000458 doi:10.1371/journal.ppat.1000458. 19478871 10.1371/journal.ppat.1000458 PMC2680619

[57] Duffus A, Olson D (2011) The establishment of a global Ranavirus reporting system. Froglog 96: 37.

[58] Marschang RE, Miller D (2011) 2011 International Ranavirus symposium. J Herpetol Med Surgery 21: 1–2.

[59] R Development Core Team (2009) R: A language and environment for statistical computing. R Foundation for Statistical Computing, Vienna, Austria ISBN 3-900051-07-0, URL http://www.R-project.org.

[60] USGS [United States Geological Survey] (2006) Global digital elevation model GTOPO30. [US Geological Survey ] global digital elevation model GTOPO30<http://edc.usgs.gov/products/elevation/gtopo30/gtopo30.html>(accessed 28 October 2008).

[61] Olson DM (2001) BioScience 933–938 http://www.worldwildlife.org/science/data/item1875.html(accessed 28 October 2008).

[62] Mitchell TD (2004) Tyndall Centre Working Paper No. 55.<http://www.tyndall.ac.uk/publications/working_papers/wp55_summary.shtml>(Accessed 4 November 2008)

