# Supplementary figures and images for: Mapping the Global Emergence of Batrachochytrium dendrobatidis, the Amphibian Chytrid Fungus
Source: PLoS One. 2013 Feb 27;8(2):e56802. doi: 10.1371/journal.pone.0056802 (PMC3584086; doi:10.1371/journal.pone.0056802)

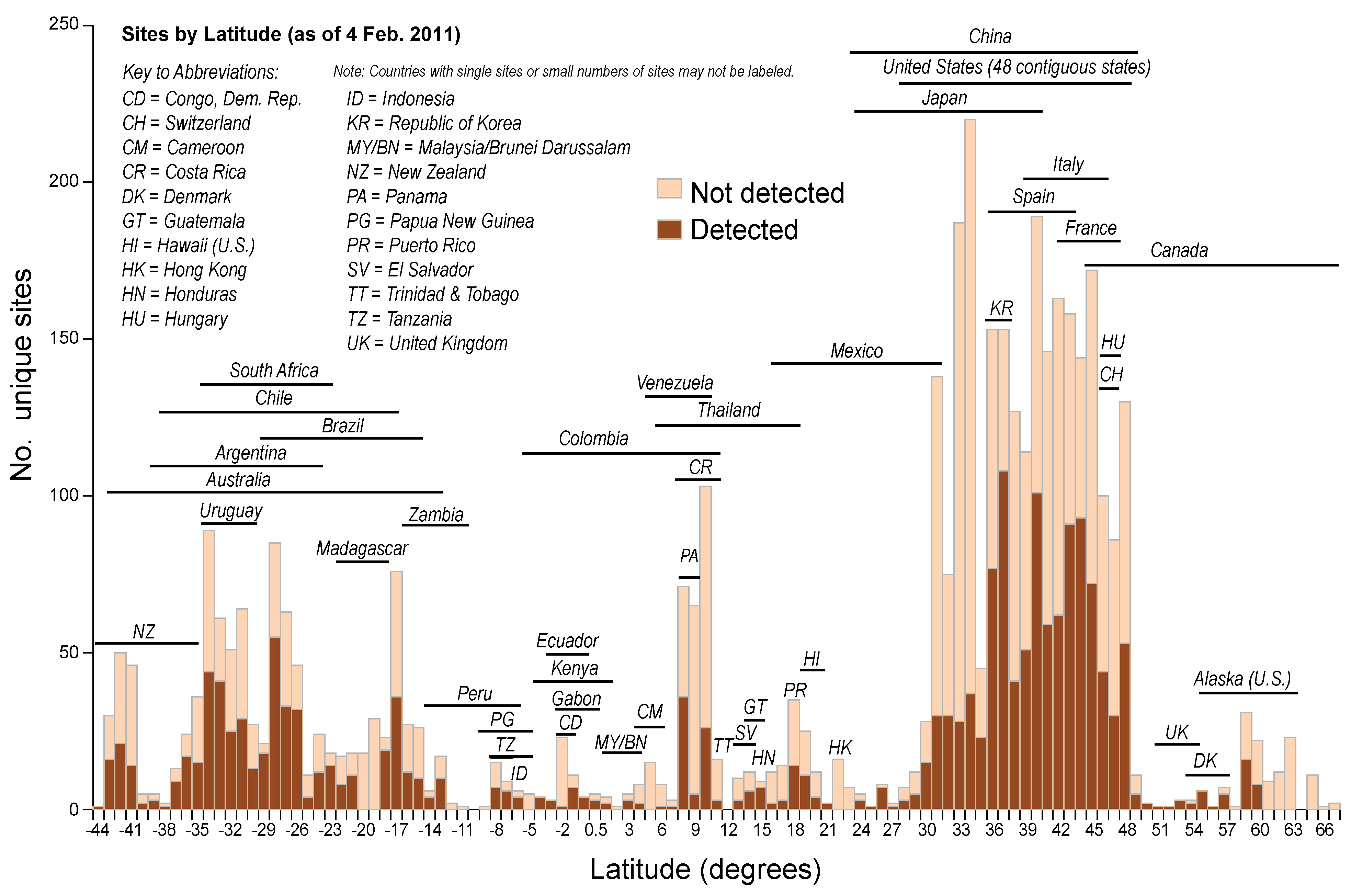

Supplement: Figure S1 — Occurrence of Batrachochytrium dendrobatidis ( Bd ) at sites by latitude. The high number of sample sites in the Northern Hemisphere, particularly the United States and Spain, gives a pronounced skew to this distribution. (TIF) [file pone.0056802.s001.tif]

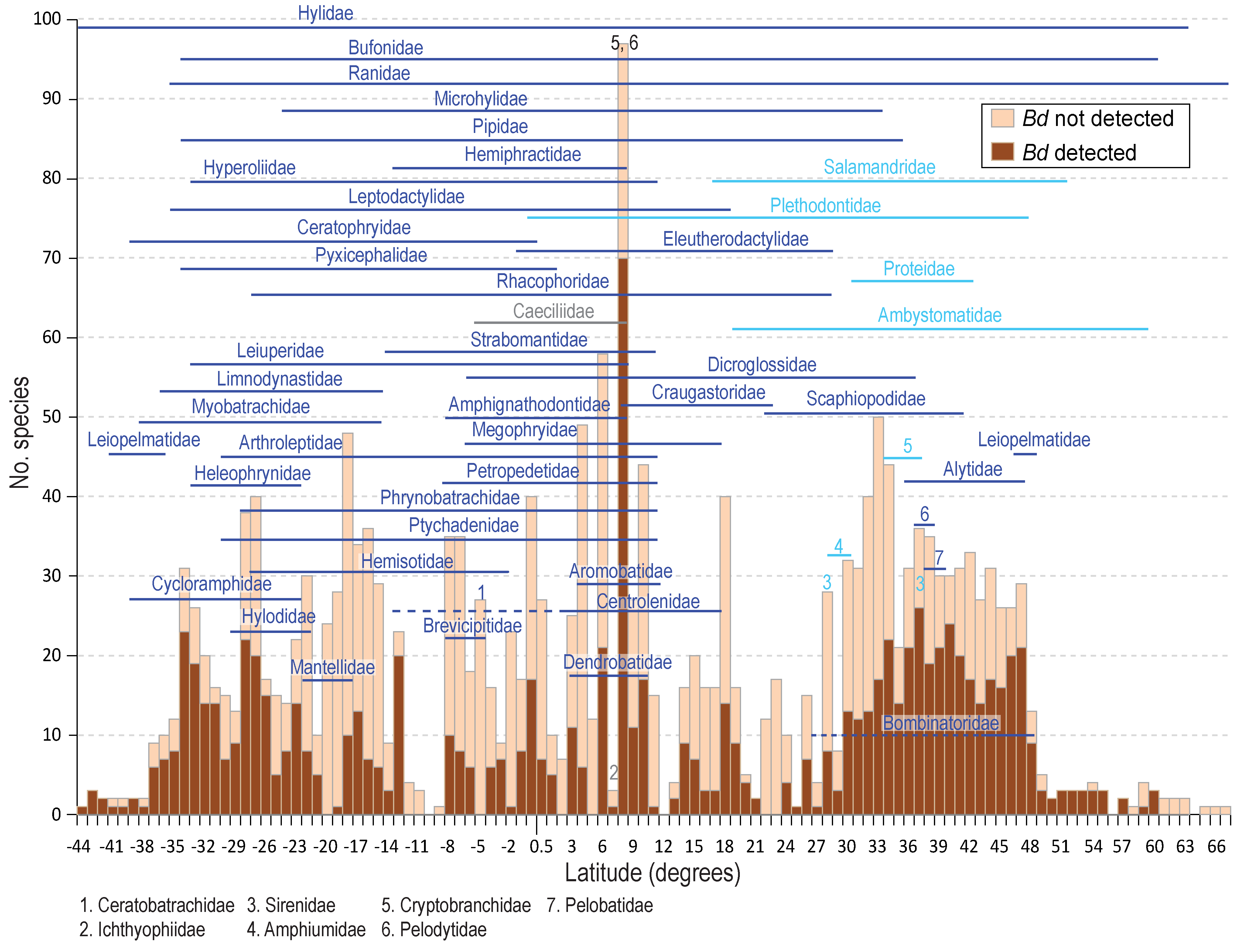

Supplement: Figure S2 — Taxonomic patterns of Batrachochytrium dendrobatidis ( Bd ) occurrence by latitude. The extraordinary peak in species richness in the 8°N range highlights the overlap of many families of amphibians in Central America and also the intensive sampling represented by Lips et al. (2003, 2006). Of additional interest are the very broad latitudinal ranges of the families Hylidae, Bufonidae, and Ranidae. This range explains, in part, why they were so widely sampled, and Bd was so widely detected among them, and therefore also why they were chosen as the three families we modeled. Note also how the number of families thins out at very high and very low latitudes. (TIF) [file pone.0056802.s002.tif]

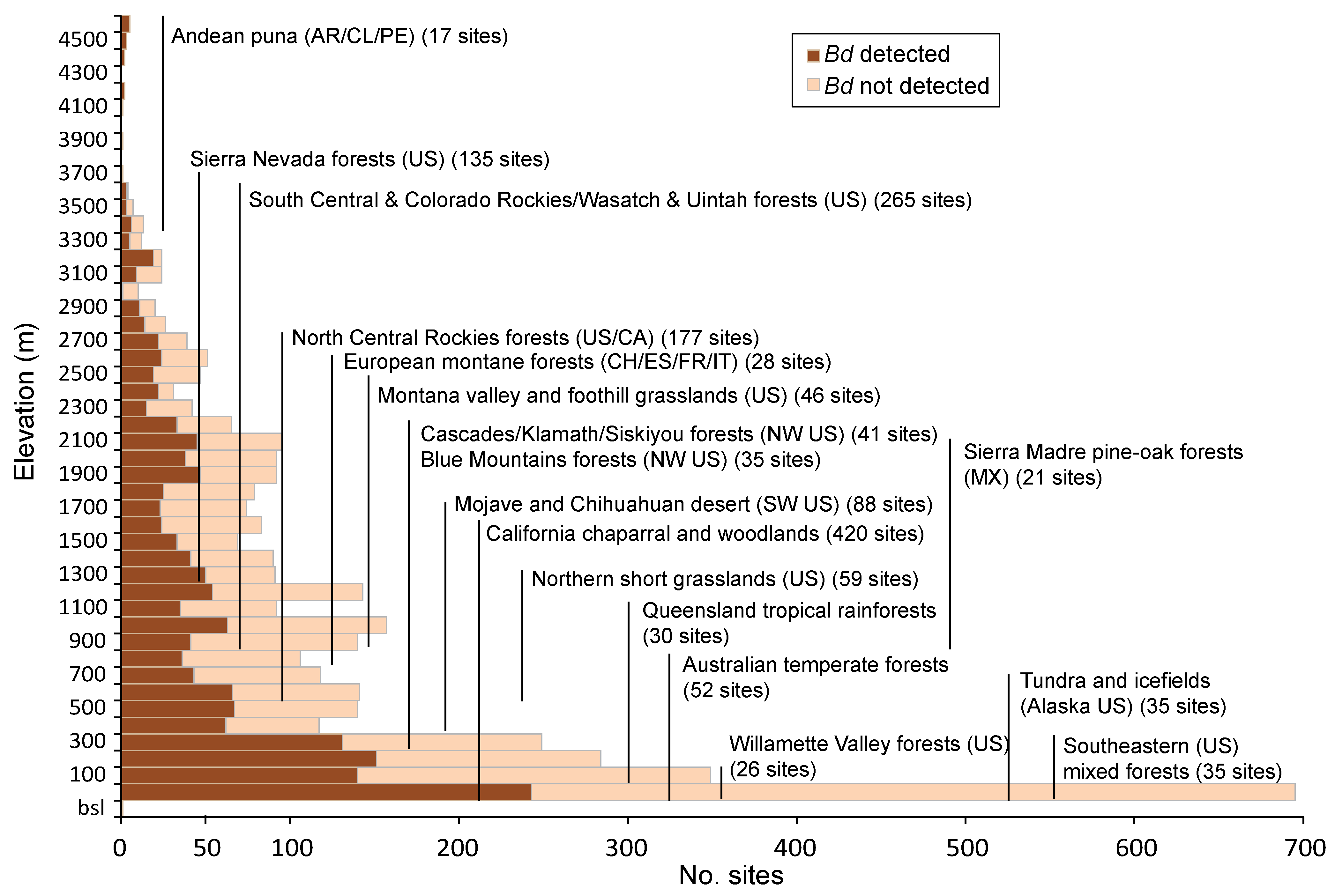

Supplement: Figure S3 — Occurrence of Batrachochytrium dendrobatidis at sites by elevation, with annotation by ecoregion of site. Vertical black lines indicate the range of elevations covered by a particular ecoregion. For the most part, only ecoregions or aggregates of ecoregions with more than 20 sites represented are shown in the annotation. Low elevation sites (below 1000 m) were broadly distributed across the world, and across a wide variety of ecoregions. ‘bsl’ = ‘below sea level’, for a site in the Coachella Valley, California, U.S.A. Country abbreviations: AR = Argentina, CA = Canada, CH = Switzerland, CL = Chile, ES = Spain, FR = France, IT = Italy, MX = Mexico, PE = Peru, US = United States. (TIF) [file pone.0056802.s003.tif]

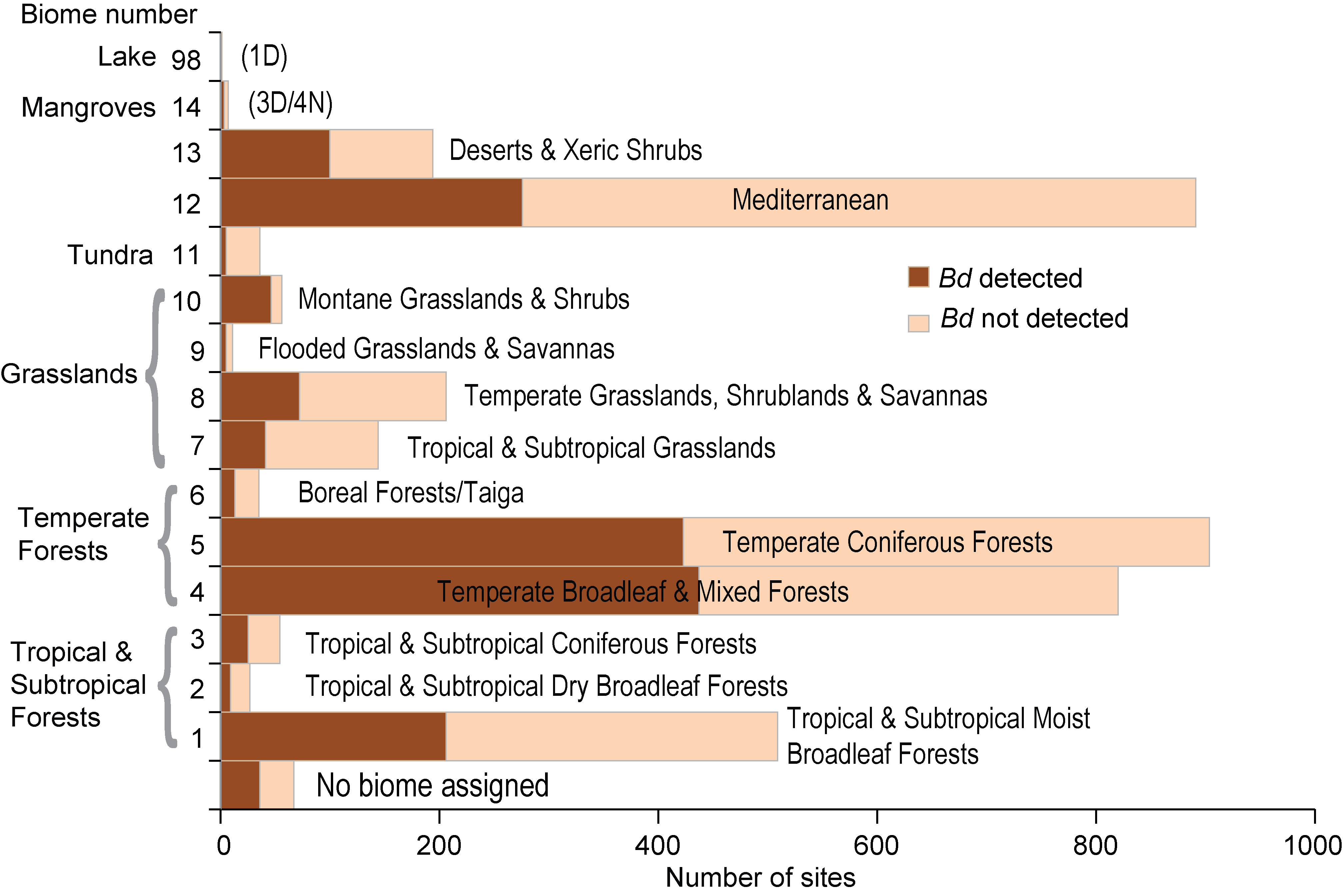

Supplement: Figure S4 — Occurrence of Batrachochytrium dendrobatidis ( Bd ) at sites among 15 world biomes. ‘Lake’ (98) was dropped from our analyses, as there was only one site so classified owing to the coarse scale of the bioregional data relative to our site locations. Among biomes, the highest odds of detecting Bd were in Montane Grasslands and Shrublands (Biome 10, reported from Australia, New Zealand, South Africa, and the Chilean and Peruvian Andes), and the lowest odds of detection were in Mediterranean Forests, Woodlands, and Scrub (Biome 12, based on sites in Australia, Spain, Italy, South Africa, and California (U.S.A.), and in Tundra (Biome 11, based on sites in the interior and Kenai Peninsula of Alaska, U.S.A.). (TIF) [file pone.0056802.s004.tif]

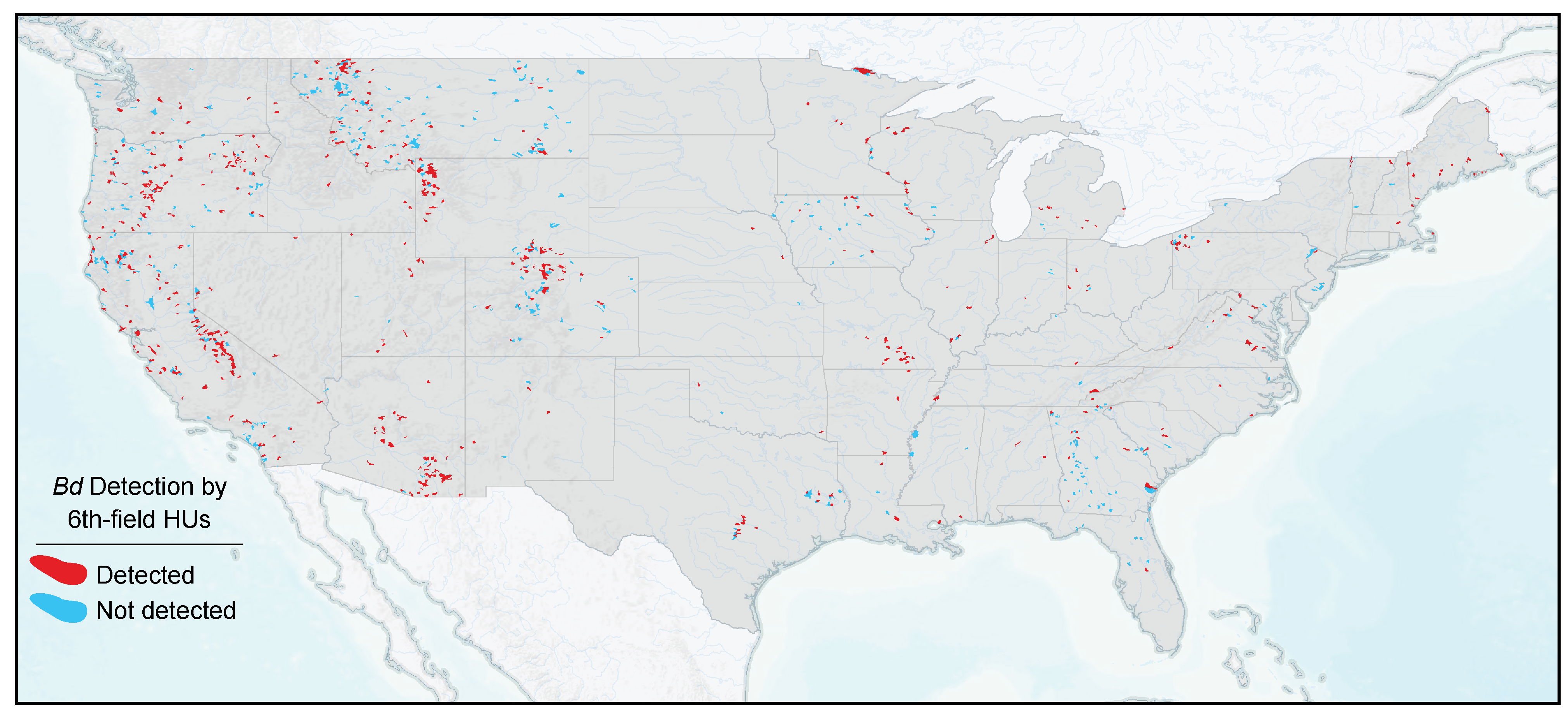

Supplement: Figure S5 — Batrachochytrium dendrobatidis ( Bd ) occurrence by 6th-field Hydrologic Units (HU; watershed) for the U.S.A. Natural resource planning and management decisions often occur by watershed in the US. For example, disease disinfection protocols often stipulate disinfection between drainages, and 6th-field watershed delineations are used for water draw decisions during wildfire season in parts of the US. Aquatic invasive species including Bd are mapped by watershed to inform decision-makers about risk of transmission during water draw decisions. Map reflects data as of January 2011. (TIF) [file pone.0056802.s005.tif]
