# Supplementary material for: Mapping the Global Emergence of Batrachochytrium dendrobatidis, the Amphibian Chytrid Fungus
Source: PLoS One. 2013 Feb 27;8(2):e56802. doi: 10.1371/journal.pone.0056802 (PMC3584086; doi:10.1371/journal.pone.0056802)
Supplement: Table S1 — Bd detections by species, with references and countries of detection. Family-level taxonomy is shown according to Frost et al. (2006), Grant et al. (2006), Frost (2009), and Hedges et al. (2008). Species name is shown as given in the report of the Bd occurrence. Where assignments to genus or species have changed since the species was reported as being found with Bd, the older name is given in parentheses. For further information on taxonomy, see Taxonomic Notes. The abbreviation (cap.) after a species name indicates an infected captive animal or animals. Status is Conservation Status according to the IUCN Red List (IUCN 2010). Categories are defined as: EX = extinct; EW = Extinct in the Wild; CR = Critically Endangered; EN = Endangered; VU = Vulnerable (the previous three categories are considered Threatened by the IUCN); NT = Near Threatened; LC = Least Concern; DD = Data Deficient; NE = Not Evaluated (newly discovered or newly recognized species). Last updated from published literature in March 2011; references are given in Supplemental Information text, File S1. (DOCX) [file pone.0056802.s006.docx]

**Table S1:** *Bd* detections by species, with references and countries of detection. Family-level taxonomy is shown according to Frost et al. (2006), Grant et al. (2006), Frost (2009), and Hedges et al. (2008). Species name is shown as given in the report of the *Bd* occurrence. Where assignments to genus or species have changed since the species was reported as being found with *Bd*, the older name is given in parentheses. For further information on taxonomy, see Taxonomic Notes. The abbreviation (cap.) after a species name indicates an infected captive animal or animals. Status is Conservation Status according to the IUCN Red List (IUCN 2010). Categories are defined as: EX = extinct; EW = Extinct in the Wild; CR = Critically Endangered; EN = Endangered; VU = Vulnerable (the previous three categories are considered Threatened by the IUCN); NT = Near Threatened; LC = Least Concern; DD = Data Deficient; NE = Not Evaluated (newly discovered or newly recognized species). Last updated from published literature in March 2011; references are given in Supplemental Information text, File S1.

**Part 1. Anura**

| **Family** | **Species** | **Country** | **Reference** | **Status** |
| --- | --- | --- | --- | --- |
| Alytidae^[[1]](#footnote-1)^ | *Alytes muletensis* | Spain | Walker et al. (2008); S. Walker, unpub. | VU |
| Alytidae^1^ | *Alytes obstetricans* | France, Spain, Switzerland | Bosch et al. (2001); Garner et al. (2005); S. Walker, unpub. | LC |
| Alytidae^1^ | *Discoglossus jeannae* | Spain | S. Walker, unpub. | NT |
| Alytidae^1^ | *Discoglossus sardus* | Italy | Bielby et al. (2009) | LC |
| Alytidae^1^ | *Discoglossus scovazzi* | Morocco | El Mouden et al. (2011) | LC |
| Aromobatidae | *Allobates (Colostethus) talamancae* | Panama | Lips et al. (2006) | LC |
| Aromobatidae | *Aromobates meridensis* | Venezuela | Lampo et al. (2008) | CR |
| Aromobatidae | *Mannophryne collaris* | Venezuela | Sánchez et al. (2008) | EN |
| Aromobatidae | *Mannophryne cordilleriana* | Venezuela | Lampo et al. (2006a) | CR |
| Aromobatidae | *Mannophryne herminae* | Venezuela | Nicolás (2007) | NT |
| Aromobatidae | *Mannophryne olmonae* | Trinidad & Tobago | Alemu et al. (2008) | CR |
| Arthroleptidae | *Leptopelis christyi* | Uganda | Goldberg et al. (2007) | LC |
| Arthroleptidae | *Leptopelis kivuensis* | Uganda | Goldberg et al. (2007) | NT |
| Arthroleptidae | *Leptopelis spiritusnoctis* | Nigeria | Imasuen et al. (2011) | LC |
| Arthroleptidae | *Leptopelis viridis (hyloides)* | Nigeria | Imasuen et al. (2011) | LC |
| Bombinatoridae | *Bombina pachypus* | Italy | Stagni et al. (2002) | EN |
| Bombinatoridae | *Bombina variegata* | Hungary | J. Vörös, E. Sos, R. Dankovics, A. Hettyey, F. Hock, unpub. | LC |
| Bufonidae | *Amietophrynus (Bufo) funereus* | Uganda | Goldberg et al. (2007) | LC |
| Bufonidae | *Amietophrynus gutturalis* | Kenya | Kielgast et al. (2009) | LC |
| Bufonidae | *Amietophrynus rangeri* | South Africa | C. Weldon, unpub. | LC |
| Bufonidae | *Amietophrynus regularis* | Kenya | Kielgast et al. (2009) | LC |
| Bufonidae | *Anaxyrus (Bufo) americanus* | Canada, United States | Ouellet et al. (2005); Longcore et al. (2007); Zippel and Tabaka (2008); Rodriguez et al. (2009) | LC |
| Bufonidae | *Anaxyrus (Bufo) baxteri* | United States | A. Pessier, unpub. | EW |
| Bufonidae | *Anaxyrus (Bufo) boreas* | Canada, United States | Raverty and Reynolds (2001); Morehouse et al. (2003); Muths et al. (2003); Thompson et al. (2004); Green and Muths (2005); Adams et al. (2007); Pearl et al. (2007); Young et al. (2007); Muths et al. (2008); Deguise and Richardson (2009); Hasken et al. (2009); Murphy et al. (2009); Schock et al. (2009); Slough (2009); Adams et al. (2010); Pilliod et al. (2010); unpub. data from E. Bull; D. E. Green; P. Johnson; M. Hahr; K. Kendell; B. Maxell; J. Moore; D. Pilliod and E. Muths; D. Sumerlin; S. Wagner and J. Johnson; C. Tait; Canadian Cooperative Wildlife Health Centre | NT |
| Bufonidae | *Anaxyrus (Bufo) californicus* | United States | R. Fisher, unpub.; D.E. Green, unpub. | EN |
| Bufonidae | *Anaxyrus (Bufo) canorus* | United States | Carey et al. (1999); Green and Kagarise Sherman (2001); C. Brown, unpub.; A. Lind and R. Grasso, unpub.; G. Padgett-Flohr, unpub.; U.S. Forest Service Sierra Nevada Amphibian Monitoring Program, unpub. | EN |
| Bufonidae | *Anaxyrus (Bufo) houstonensis* | United States | Gaertner et al. (2007, 2010) | EN |
| Bufonidae | *Anaxyrus (Bufo) punctatus* | United States | Sredl et al. (2002); R. Fisher, unpub. | LC |
| Bufonidae | *Anaxyrus (Bufo) terrestris* | United States | Rizkalla (2010) | LC |
| Bufonidae | *Anaxyrus (Bufo) woodhousii* | United States | Carey and Livo (2009); Saenz et al. (2010); B. Maxell, unpub. | LC |
| Bufonidae | *Atelopus bomolochos* | Ecuador | Ron and Merino-Viteri (2000) | CR |
| Bufonidae | *Atelopus carbonerensis* | Venezuela | Lampo et al. (2006a) | CR |
| Bufonidae | *Atelopus chiriquiensis* | Costa Rica, Panama | Lips et al. (2003); Berger et al. (1998); Puschendorf et al. (2009) | CR |
| Bufonidae | *Atelopus cruciger* | Venezuela | Bonaccorso et al. (2003) | CR |
| Bufonidae | *Atelopus ignescens* | Ecuador | Ron and Merino-Viteri (2000) | EX |
| Bufonidae | *Atelopus mittermeieri* | Colombia | Ruiz and Rueda-Almonacid (2008) | EN |
| Bufonidae | *Atelopus mucabajiensis* | Venezuela | Lampo et al. (2006a, 2006b) | CR |
| Bufonidae | *Atelopus patazensis* | Peru | Venegas et al. (2008) | NE |
| Bufonidae | *Atelopus sorianoi* | Venezuela | Lampo et al. (2006a) | CR |
| Bufonidae | *Atelopus varius* | Costa Rica, Panama | Berger et al. (1998); Puschendorf (2003); Puschendorf et al. (2006a, 2009) | CR |
| Bufonidae | *Atelopus zeteki* | Panama | Lips et al. (2006) | CR |
| Bufonidae | *Bufo bufo* | Spain, United Kingdom | Garner et al. (2005); Bosch and Martinez-Solano (2006); J. Bosch, unpub.; S. Walker, unpub. | LC |
| Bufonidae | *Bufo gargarizans* | Rep. of Korea | Yang et al. 2009 | LC |
| Bufonidae | *Bufo japonicus* | Japan | S. Okada, unpub. | LC |
| Bufonidae | *Epidalea (Bufo) calamita* | United Kingdom, Spain | T. Garner, unpub.; S. Walker, unpub. | LC |
| Bufonidae | *Incilius (Bufo) nebulifer* | United States | Gaertner et al. (2010) | LC |
| Bufonidae | *Incilius (Bufo) occidentalis* | Mexico | D. E. Green, unpub. | LC |
| Bufonidae | *Incilius (Bufo) periglenes* | Costa Rica | Lips et al. (2006) | EX |
| Bufonidae | *Leptophryne cruentata* | Indonesia | Kusrini et al. (2008) | CR |
| Bufonidae | *Melanophryniscus moreirae* | Brazil | Ferriera et al. (2008) | NT |
| Bufonidae | *Nectophrynoides asperginis* | Tanzania | Weldon and du Preez (2004) | EW |
| Bufonidae | *Nectophrynoides spp.^[[2]](#footnote-2)^* | Tanzania | Moyer and Weldon (2006) | NE |
| Bufonidae | *Peltophryne (Bufo) longinasus dunni* | Cuba | Díaz et al. (2007) | EN |
| Bufonidae | *Peltophryne (Bufo) taladai* | Cuba | A. Rodriguez, unpub. | VU |
| Bufonidae | *Rhaebo (Bufo) haematiticus* | Panama | Berger et al. (1998); Lips et al. (2006) | LC |
| Bufonidae | *Rhinella inca* | Peru | Catenazzi et al. (2011) | LC |
| Bufonidae | *Rhinella marina (Bufo marinus)* | Australia, Panama, Venezuela | Berger et al. (1998); Lips et al. (2006); Sánchez et al. (2008); K. Murray, unpub. | LC |
| Bufonidae | *Rhinella quechua* | Bolivia | Barrionuevo et al. (2008) | VU |
| Bufonidae | *Rhinella (Bufo) spinulosus* | Chile | R. Solis, unpub. | LC |
| Bufonidae | *Vandijkophrynus (Bufo) robinsoni* | South Africa | Weldon (2005) | LC |
| Calyptocephalellidae | *Calyptocephalella gayi* | Chile | Soto-Azat and Cunningham (2010) | VU |
| Centrolenidae | *Centrolene buckleyi* | Colombia | Velásquez-E. et al. (2008) | VU |
| Centrolenidae | *Centrolene ilex* | Panama | Lips et al. (2006) | LC |
| Centrolenidae | *Cochranella albomaculata* | Panama | Berger et al. (1998); Lips et al. (2006) | LC |
| Centrolenidae | *Cochranella euknemos* | Panama | Lips et al. (2006) | LC |
| Centrolenidae | *Espadarana (Centrolene, Centrolenella, Cochranella) prosoblepon* | Costa Rica, Panama | Berger et al. (1998); Lips et al. (2006); Picco and Collins (2007) | LC |
| Centrolenidae | *Hyalinobatrachium bergeri* | Peru | Catenazzi et al. (2011) | LC |
| Centrolenidae | *Hyalinobatrachium colymbiphyllum* | Panama | Lips et al. (2006) | LC |
| Centrolenidae | *Hyalinobatrachium valerioi* | Costa Rica | Goldberg et al. (2009) | LC |
| Centrolenidae | *Nymphargus (Cochranella) griffithsi* | Colombia | Velásquez-E. et al. (2008) | VU |
| Ceratophryidae | *Atelognathus patagonicus* | Argentina | Fox et al. (2006) | EN |
| Ceratophryidae | *Ceratophrys calcarata* | Japan (cap.) | Une et al. (2008) | LC |
| Ceratophryidae | *Ceratophrys cornuta* | Japan (cap.) | Une et al. (2008); Goka et al. (2009) | LC |
| Ceratophryidae | *Ceratophrys cranwelli* | Japan (cap.) | Une et al. (2008); Goka et al. (2009) | LC |
| Ceratophryidae | *Ceratophrys ornata* | Japan (cap.) | Goka et al. (2009) | NT |
| Ceratophryidae | *Chacophrys pierottii* | Japan (cap.) | Une et al. (2008) | LC |
| Ceratophryidae | *Lepidobatrachus laevis* | Japan (cap.) | Une et al. (2008); Goka et al. (2009) | LC |
| Ceratophryidae | *Telmatobius atacamensis* | Argentina | Barrionuevo and Mangione (2006) | CR |
| Ceratophryidae | *Telmatobius marmoratus* | Chile, Peru | Seimon et al. (2007); Catenazzi et al. (2010, 2011) ; I. De la Riva, unpub.; R. Solis, unpub. | VU |
| Ceratophryidae | *Telmatobius niger* | Ecuador | Berger et al. (1999); Ron and Merino-Viteri (2000) | CR |
| Ceratophryidae | *Telmatobius pisanoi* | Argentina | Barrionuevo and Mangione (2006) | EN |
| Ceratophryidae | *Telmatobius spp.* | Chile | R. Solis, unpub. | NE |
| Craugastoridae^[[3]](#footnote-3)^ | *Craugastor (Eleutherodactylus) aurilegulus* | Honduras | Puschendorf et al. (2006b) | EN |
| Craugastoridae | *Craugastor azueroensis* | Panama | Lips et al. (2006) | EN |
| Craugastoridae | *Craugastor bransfordii* | Costa Rica, Panama | Lips et al. (2006); Puschendorf et al. (2006a, 2009); Saenz et al. (2009) | LC |
| Craugastoridae | *Craugastor crassidigitus* | Costa Rica, Panama | Lips et al. (2006); Puschendorf et al. (2006a, 2009); Saenz et al. (2009) | LC |
| Craugastoridae | *Craugastor (Eleutherodactylus) emcelae* | Panama | Berger et al. (1998) | CR |
| Craugastoridae | *Craugastor fitzingeri* | Costa Rica | Puschendorf et al. (2006a, 2009); Goldberg et al. (2009) | LC |
| Craugastoridae | *Craugastor gollmeri* | Panama | Lips et al. (2006) | LC |
| Craugastoridae | *Craugastor megacephalus* | Panama | Lips et al. (2006) | LC |
| Craugastoridae | *Craugastor (Eleutherodactylus) melanostictus* | Costa Rica | Lips et al. (2003); Puschendorf et al. (2009) | LC |
| Craugastoridae | *Craugastor noblei* | Costa Rica, Panama | Lips et al. (2006); Saenz et al. (2009) | LC |
| Craugastoridae | *Craugastor podiciferus* | Costa Rica, Panama | Lips et al. (2006); Puschendorf et al. (2006a, 2009) | NT |
| Craugastoridae | *Craugastor punctariolus* | Panama | Lips et al. (2006) | EN |
| Craugastoridae | *Craugastor rostralis* | Honduras | Kolby et al. (2010) | NT |
| Craugastoridae | *Craugastor (Eleutherodactylus) saltator (mexicanus)* | Mexico | Lips et al. (2004) | LC |
| Craugastoridae | *Craugastor tabasarae* | Panama | Lips et al. (2006) | CR |
| Craugastoridae | *Craugastor talamancae* | Costa Rica, Panama | Lips et al. (2006); Puschendorf et al. (2006a, 2009) | LC |
| Craugastoridae | *Craugastor (Eleutherodactylus) underwoodi* | Costa Rica | Picco and Collins (2007) | LC |
| Cycloramphidae | *Rhinoderma darwinii* | Chile | Bourke et al. (2010) | VU |
| Cycloramphidae | *Odontophrynus maisuma* | Uruguay | Borteiro et al. (2009) | NE |
| Cycloramphidae | *Odontophrynus occidentalis* | Argentina | Gutierrez et al. (2010) | LC |
| Cycloramphidae | *Thoropa taophora* | Brazil | Toledo et al. (2006a) | NE |
| Dendrobatidae | *Colostethus fraterdanieli* | Colombia | Velásquez-E. et al. (2008) | NT |
| Dendrobatidae | *Colostethus inguinalis* | Panama | K Lips, unpub. | LC |
| Dendrobatidae | *Colostethus panamensis* | Panama | Lips et al. (2006) | LC |
| Dendrobatidae | *Colostethus pratti* | Panama | Lips et al. (2006) | LC |
| Dendrobatidae | *Dendrobates auratus* | Costa Rica, United States | Pessier et al. (1999); Saenz et al. (2009) | LC |
| Dendrobatidae | *Dendrobates tinctorius (azureus)* | United States | Pessier et al. (1999) | LC |
| Dendrobatidae | *^[[4]](#footnote-4)^Hyloxalus abditaurantius* | Colombia | Velásquez-E. et al. (2008) | LC |
| Dendrobatidae | *Hyloxalus fascianigrus^[[5]](#footnote-5)^ (fascianiger)* | Colombia | Velásquez-E. et al. (2008) | NT |
| Dendrobatidae | *Hyloxalus lehmanni* | Colombia | Velásquez-E. et al. (2008) | NT |
| Dendrobatidae | *Oophaga (Dendrobates) pumilio* | Costa Rica | Puschendorf et al. (2006a, 2009); Saenz et al. (2009) | LC |
| Dendrobatidae | *Phyllobates lugubris* | Belgium (cap.), Costa Rica (wild), Germany (cap.) | Mutschmann et al. (2000); Saenz et al. (2009) | LC |
| Dendrobatidae | *Phyllobates vittatus* | Germany (cap.) | Mutschmann et al. (2000) | EN |
| Dendrobatidae | *Silverstoneia (Colostethus) flotator* | Panama | Lips et al. (2006) | LC |
| Dendrobatidae | *Silverstoneia (Colostethus) nubicola* | Panama | Lips et al. (2006) | LC |
| Dicroglossidae | *Fejervarya limnocharis* | Japan | Goka et al. (2009) | LC |
| Dicroglossidae | *Limnonectes macrocephalus* | Philippines | A. Diesmos and R. Brown, unpub.^[[6]](#footnote-6)^ | NT |
| Dicroglossidae | *Limnonectes microdiscus* | Indonesia | Kusrini et al. (2008) | LC |
| Dicroglossidae | *Limnonectes woodworthi* | Philippines | A. Diesmos and R. Brown, unpub.^6^ | LC |
| Dicroglossidae | *Occidozyga laevis* | Philippines | A. Diesmos and R. Brown, unpub. ^6^ | LC |
| Dicroglossidae | *Occidozyga lima* | Japan (cap.) | Une et al. (2008) | LC |
| Eleutherodactylidae^[[7]](#footnote-7)^ | *Eleutherodactylus antillensis* | Puerto Rico | Burrowes et al. (2008) | LC |
| Eleutherodactylidae | *Eleutherodactylus brittoni* | Puerto Rico | Burrowes et al. (2008) | LC |
| Eleutherodactylidae | *Eleutherodactylus coqui* | Puerto Rico, United States | Beard and O'Neill (2005); Burrowes et al. (2008); Longo et al. (2010); D. E. Green, unpub. | LC |
| Eleutherodactylidae | *Diasporus (Eleutherodactylus) diastema* | Costa Rica, Panama | Lips et al. (2006); Saenz et al. (2009) | LC |
| Eleutherodactylidae | *Diasporus(Eleutherodactylus) vocator* | Panama | Lips et al. (2006) | LC |
| Eleutherodactylidae | *Eleutherodactylus karlschmidti* | Puerto Rico | Burrowes et al. (2008); D. E. Green, unpub. | CR |
| Eleutherodactylidae | *Eleutherodactylus locustus* | Puerto Rico | Burrowes et al. (2008); P. Burrowes, A. Longo, & R. Joglar, unpub. | CR |
| Eleutherodactylidae | *Eleutherodactylus patriciae* | Dominican Republic | Joglar et al. (2007) | EN |
| Eleutherodactylidae | *Eleutherodactylus pituinus* | Dominican Republic | Joglar et al. (2007) | EN |
| Eleutherodactylidae | *Eleutherodactylus planirostris* | United States | Rizkalla (2010) | LC |
| Eleutherodactylidae | *Eleutherodactylus portoricensis* | Puerto Rico | Burrowes et al. (2008); Longo et al. (2010) | EN |
| Eleutherodactylidae | *Eleutherodactylus richmondi* | Puerto Rico | Burrowes et al. (2008); P. Burrowes, A. Longo, & R. Joglar, unpub. | CR |
| Eleutherodactylidae | *Eleutherodactylus unicolor* | Puerto Rico | Burrowes et al. (2008) | VU |
| Eleutherodactylidae | *Eleutherodactylus wightmanae* | Puerto Rico | Burrowes et al. (2008) | EN |
| Heleophrynidae | *Hadromophryne (Heleophryne) natalensis* | Lesotho, South Africa | Smith et al. (2007); C. Weldon, unpub. | LC |
| Heleophrynidae | *Heleophryne purcelli* | South Africa | Weldon (2005) | LC |
| Heleophrynidae | *Heleophryne regis* | South Africa | Weldon (2005) | LC |
| Hemiphractidae | *Gastrotheca (Hyla) antoniiochoai* | Peru | Catenazzi et al. (2011) | DD |
| Hemiphractidae | *Gastrotheca cornuta* | Panama | Lips et al. (2006) | EN |
| Hemiphractidae | *Gastrotheca dendronastes* | Colombia | Velásquez-E. et al. (2008) | VU |
| Hemiphractidae | *Gastrotheca excubitor* | Peru | Catenazzi et al. (2011) | VU |
| Hemiphractidae | *Gastrotheca nicefori* | Venezuela | Sánchez et al. (2008) | LC |
| Hemiphractidae | *Gastrotheca ochoai* | Peru | Catenazzi et al. (2011) | DD |
| Hemiphractidae | *Gastrotheca pseustes* | Ecuador | Ron and Merino-Viteri (2000) | EN |
| Hylidae | *Acris crepitans* | United States | Pessier et al. (1999); Rothermel et al. (2008); Saenz et al. (2010); J. Krebs, unpub.; J. Ware and K. Duncan, unpub. | LC |
| Hylidae | *Acris (crepitans) blanchardi* (listed as Threatened in several states, Endangered in Canada) | United States | Steiner and Lehtinen (2008); Zippel and Tabaka (2008); Gaertner et al. (2009 Herp Rev) | NE |
| Hylidae | *Acris gryllus* | United States | Rizkalla (2009, 2010) | LC |
| Hylidae | *Agalychnis callidryas* | Panama | Lips et al. (2006) | LC |
| Hylidae | *Agalychnis moreletii* | Mexico, El Salvador | Felger et al. (2007); Frías-Alvarez et al. (2008) | CR |
| Hylidae | *Aplastodiscus callipygius* | Brazil | Toledo et al. (2006a) | LC |
| Hylidae | *Aplastodiscus leucopygius* | Brazil | Toledo et al. (2006a) | LC |
| Hylidae | *Bokermannohyla circumdata* | Brazil | Toledo et al. (2006a) | LC |
| Hylidae | *Bokermannohyla hylax* | Brazil | Toledo et al. (2006a) | LC |
| Hylidae | *Dendropsophus ebraccatus (Hyla ebraccata)* | Costa Rica | Picco and Collins (2007) | LC |
| Hylidae | *Dendropsophus meridensis* | Venezuela | Sánchez et al. (2008) | EN |
| Hylidae | *Dendropsophus (Hyla) microcephala* | Panama | Lips et al. (2006) | LC |
| Hylidae | *Dendropsophus parviceps* | Peru | I. De la Riva, unpub. | LC |
| Hylidae | *Duellmanohyla rufioculis* | Costa Rica | Picco and Collins (2007) | LC |
| Hylidae | *Duellmanohyla soralia* | Honduras | Kolby et al. (2010) | CR |
| Hylidae | *Duellmanohyla uranochroa* | Costa Rica | Puschendorf et al. (2006a, 2009) | CR |
| Hylidae | *Exerodonta melanomma* | Mexico | Frías-Alvarez et al. (2008) | VU |
| Hylidae | *Hyla arborea* | Spain | S. Walker, unpub. | LC |
| Hylidae | *Hyla arenicolor* | United States | Bradley et al. (2002); Arizona Game & Fish Dept, unpub. | LC |
| Hylidae | *Hyla chrysoscelis* | United States | Rothermel et al. (2008) | LC |
| Hylidae | *Hyla cinerea* | United States | Longcore et al. (2007) | LC |
| Hylidae | *Hyla euphorbiacea* | Mexico | Frías-Alvarez et al. (2008) | NT |
| Hylidae | *Hyla eximia* | Mexico | Frías-Alvarez et al. (2008) | LC |
| Hylidae | *Hyla japonica* | Japan, Rep. of Korea | Yang et al. (2009); Goka et al. (2009); S. Okada, unpub. | LC |
| Hylidae | *Hyla meridionalis* | Morocco, Spain | El Mouden et al. (2011); S. Walker, unpub. | LC |
| Hylidae | *Hyla sarda* | Italy | J. Bielby, T. Garner, & S. Bovero, unpub. | LC |
| Hylidae | *Hyla versicolor* | Canada, United States | Ouellet et al. (2005); Rothermel et al. (2008) | LC |
| Hylidae | *Hyla wrightorum* | United States | Sredl et al. (2002) | LC |
| Hylidae | *Hylomantis (Phyllomedusa) lemur* | Panama | Lips et al. (2006) | CR |
| Hylidae | *Hyloscirtus alytolylax* | Colombia | Velásquez-E. et al. (2008) | NT |
| Hylidae | *Hyloscirtus armatus* | Peru | Catenazzi et al. (2011) | LC |
| Hylidae | *Hyloscirtus bogotensis* | Colombia | Ruiz and Rueda-Almonacid (2008) | NT |
| Hylidae | *Hyloscirtus colymba* | Panama | Lips et al. (2006) | CR |
| Hylidae | *Hyloscirtus palmeri* | Panama | Lips et al. (2006) | LC |
| Hylidae | *Hyloscirtus platydactylus* | Venezuela | Sánchez et al. (2008) | VU |
| Hylidae | *Hyloscirtus (Hyla) psarolaima* | Ecuador | Ron and Merino-Viteri (2000) | EN |
| Hylidae | *Hypsiboas albopunctatus* | Brazil | Toledo et al. (2006a) | LC |
| Hylidae | *Hypsiboas balzani* | Peru | Catenazzi et al. (2011) | LC |
| Hylidae | *Hypsiboas cordobae* | Argentina | Gutierrez et al. (2010) | DD |
| Hylidae | *Hypsiboas crepitans* | Venezuela | Sánchez et al. (2008) | LC |
| Hylidae | *Hypsiboas pulchellus* | Uruguay | Borteiro et al. (2009) | LC |
| Hylidae | *Hypsiboas semilineatus* | Brazil | Toledo et al. (2006a) | LC |
| Hylidae | *Isthmohyla pseudopuma* | Costa Rica | Puschendorf et al. (2006a, 2009); Picco and Collins (2007) | LC |
| Hylidae | *Litoria adelaidensis* | Australia | Berger et al. (1999); K. Murray, unpub. | LC |
| Hylidae | *Litoria aurea* | Australia, New Zealand | Speare and Berger (2005); A. Haigh, unpub.; K. Murray, unpub. | VU |
| Hylidae | *Litoria burrowsi (burrowsae)* | Australia | Pauza et al. 2010 | LC |
| Hylidae | *Litoria caerulea* | Australia, Japan (cap.), United States (cap.) | Berger et al. (1998, 1999); Pessier et al. (1999); Goka et al. (2009); K. Murray, unpub. | LC |
| Hylidae | *Litoria chloris* | Australia | Berger et al. (1999); Kriger and Hero (2007a); Murray et al. (2009); K. Murray, unpub. | LC |
| Hylidae | *Litoria citropa* | Australia | Berger et al. (1999) | LC |
| Hylidae | *Litoria (Nyctimystes) dayi* | Australia | Berger et al. (1999); K. Murray, unpub. | EN |
| Hylidae | *Litoria ewingi* | Australia, New Zealand | Berger et al. (1999); Obendorf (2005); Obendorf and Dalton (2006); Pauza and Driessen (2008); Pauza et al. (2010); A. Haigh, unpub.; K. Murray, unpub. | LC |
| Hylidae | *Litoria fallax* | Australia | Kriger and Hero (2007a); K. Murray, unpub. | LC |
| Hylidae | *Litoria genimaculata* | Australia | Berger et al. (1999); K. Murray, unpub. | LC |
| Hylidae | *Litoria gracilenta* | Australia | K. Murray, unpub. | LC |
| Hylidae | *Litoria infrafrenata* | Australia | Berger et al. (1999); K. Murray, unpub. | LC |
| Hylidae | *Litoria jungguy* | Australia | Retallick et al. (2004); K. Murray, unpub. | NT |
| Hylidae | *Litoria latopalmata* | Australia | Kriger and Hero (2007a); Simpkins et al. (2010); K. Murray, unpub. | LC |
| Hylidae | *Litoria lesueuri* | Australia | Berger et al. (1998, 1999); Morehouse et al. (2003); Kriger et al. (2007); Rowley et al. (2007b); K. Murray, unpub. | LC |
| Hylidae | *Litoria lorica* | Australia | K. Murray, unpub. | CR |
| Hylidae | *Litoria moorei* | Australia | Berger et al. (1999); K. Murray, unpub. | LC |
| Hylidae | *Litoria nannotis* | Australia | Berger et al. (1998); K. Murray, unpub. | EN |
| Hylidae | *Litoria nasuta* | Australia | K. Murray, unpub. | LC |
| Hylidae | *Litoria pearsoniana* | Australia | Berger et al. (1999); Kriger and Hero (2007a); Murray et al. (2009); K. Murray, unpub. | NT |
| Hylidae | *Litoria peronii* | Australia | Briggs and Burgin (2004); K. Murray, unpub. | LC |
| Hylidae | *Litoria raniformis* | Australia, New Zealand | Waldman et al. (2001); Vörös et al. (2011); A. Haigh, unpub.; K. Murray, unpub. | EN |
| Hylidae | *Litoria rheocola* | Australia | Berger et al. (1998, 1999); K. Murray, unpub. | EN |
| Hylidae | *Litoria spenceri* | Australia | Berger et al. (1998, 1999); K. Murray, unpub. | CR |
| Hylidae | *Litoria tyleri* | Australia | Kriger and Hero (2007a) | LC |
| Hylidae | *Litoria wilcoxii* | Australia | Kriger and Hero (2006, 2007); Simpkins et al. (2010); K. Murray, unpub. | LC |
| Hylidae | *Litoria xanthomera* | Australia | K. Murray, unpub. | LC |
| Hylidae | *Osteopilus septentrionalis* | United States | Rizkalla (2010) | LC |
| Hylidae | *Osteopilus vastus* | Dominican Republic | Joglar et al. (2007) | EN |
| Hylidae | *Phrynomedusa cf. marginata* | Brazil | Toledo et al. (2006a) | LC |
| Hylidae | *Plectrohyla dasypus* | Honduras | Kolby and Padgett-Flohr (2009); Kolby et al. (2010) | CR |
| Hylidae | *Pseudacris crucifer* | United States | Saenz et al. (2010); D. E. Green, unpub. | LC |
| Hylidae | *Pseudacris fouquettei* | United States | Rothermel et al. (2008); Saenz et al. (2010) | LC |
| Hylidae | *Pseudacris maculata* | Canada, United States | Muths et al. (2003); Rittmann et al. (2003); Rogers and Banulis (2004); Rodriguez et al. (2009); Schock et al. (2009); Wilson and Rogers (2009); D. E. Green, unpub.; B. Maxell, unpub.; J. Nachtmann, unpub. | LC |
| Hylidae | *Pseudacris ocularis* | United States | Rizkalla (2010) | LC |
| Hylidae | *Pseudacris regilla* | United States | Pearl et al. (2007); D. E. Green, unpub.; V. Hemingway, unpub.; P. Johnson, unpub.; G. Padgett-Flohr, unpub.; S. Wagner and J. Johnson, unpub. | LC |
| Hylidae | *Pseudacris sierra* | United States | Lowe (2009) | NE |
| Hylidae | *Pseudacris* spp. | United States | A. Pessier, unpub. |  |
| Hylidae | *Pseudacris triseriata* | United States | Green and Muths (2005); Ouellet et al. (2005); Sredl et al. (2002); Young et al. (2007); Carey and Livo (2009); D. E. Green, unpub. | LC |
| Hylidae | *Pseudis paradoxa* | Venezuela | Sánchez et al. (2008) | LC |
| Hylidae | *Ptychohyla erythromma* | Mexico | Lips et al. (2004) | EN |
| Hylidae | *Ptychohyla hypomykter* | Guatemala, Honduras | Mendelson et al. (2004); Kolby et al. (2010) | CR |
| Hylidae | *Scarthyla vigilans* | Venezuela | Sánchez et al. (2008) | LC |
| Hylidae | *Scinax albicans* | Brazil | Toledo et al. (2006a) | LC |
| Hylidae | *Scinax squalirostris* | Uruguay | Borteiro et al. (2009) | LC |
| Hylidae | *Smilisca phaeota* | Panama | Morehouse et al. (2003); Lips et al. (2006) | LC |
| Hylidae | *Smilisca sordida* | Costa Rica | Goldberg et al. (2009); Saenz et al. (2009) | LC |
| Hylodidae | *Hylodes dactylocinus* | Brazil | Toledo et al. (2006a) | DD |
| Hylodidae | *Hylodes magalhaesi* | Brazil | Carnaval et al. (2005); Toledo et al. (2006b) | DD |
| Hylodidae | *Hylodes meridionalis* | Brazil | Toledo et al. (2006a) | LC |
| Hylodidae | *Hylodes perplicatus* | Brazil | Toledo et al. (2006a) | LC |
| Hylodidae | *Hylodes phyllodes* | Brazil | Toledo et al. (2006a) | LC |
| Hylodidae | *Hylodes sp. (aff. sazimai)* | Brazil | Toledo et al. (2006a) | DD |
| Hylodidae | *Megaelosia cf. boticariana* | Brazil | Toledo et al. (2006a) | DD |
| Hylodidae | *Megaelosia massarti* | Brazil | Toledo et al. (2006a) | DD |
| Hyperoliidae | *Afrixalus dorsalis* | Nigeria | Imasuen et al. (2011) | LC |
| Hyperoliidae | *Afrixalus fornasini* | Kenya, Japan (cap.) | Goka et al. (2009); Kielgast et al. (2009) | LC |
| Hyperoliidae | *Afrixalus nigeriensis* | Nigeria | Imasuen et al. (2011) | NT |
| Hyperoliidae | *Afrixalus paradorsalis* | Nigeria | Imasuen et al. (2011) | LC |
| Hyperoliidae | *Afrixalus sp. nov.* | Tanzania | Moyer and Weldon (2006) | NE |
| Hyperoliidae | *Afrixalus sylvaticus* | Kenya | Kielgast et al. (2009) | EN |
| Hyperoliidae | *Afrixalus uluguruensis* | Tanzania | Moyer and Weldon (2006) | EN |
| Hyperoliidae | *Hyperolius acuticeps* | Kenya, Tanzania | Moyer and Weldon (2006); Kielgast et al. (2009) | LC |
| Hyperoliidae | *Hyperolius concolor* | Nigeria | Imasuen et al. (2011) | LC |
| Hyperoliidae | *Hyperolius fusciventris burtoni* | Nigeria | Imasuen et al. (2011) | LC |
| Hyperoliidae | *Hyperolius glandicolor* | Kenya | Kielgast et al. (2009) | LC |
| Hyperoliidae | *Hyperolius kivuensis* | DR Congo, Kenya, Uganda | Goldberg et al. (2007); Greenbaum et al. (2008); Kielgast et al. (2009) | LC |
| Hyperoliidae | *Hyperolius kuligae* | DR Congo | Greenbaum et al. (2008) | LC |
| Hyperoliidae | *Hyperolius lateralis* | Kenya | Kielgast et al. (2009) | LC |
| Hyperoliidae | *Hyperolius picturatus* | Nigeria | Imasuen et al. (2011) | LC |
| Hyperoliidae | *Hyperolius pictus* | Tanzania | Moyer and Weldon (2006) | LC |
| Hyperoliidae | *Hyperolius pseudargus* | Tanzania | Moyer and Weldon (2006) | LC |
| Hyperoliidae | *Hyperolius puncticulatus* | Tanzania | Moyer and Weldon (2006) | EN |
| Hyperoliidae | *Hyperolius sp. 1* | Nigeria | Imasuen et al. (2011) | NE |
| Hyperoliidae | *Hyperolius sp. 2* | Nigeria | Imasuen et al. (2011) | NE |
| Hyperoliidae | *Hyperolius sp. 3* | Nigeria | Imasuen et al. (2011) | NE |
| Hyperoliidae | *Hyperolius sp. 5* | Nigeria | Imasuen et al. (2011) | NE |
| Hyperoliidae | *Hyperolius spinigularis* | Tanzania | Moyer and Weldon (2006) | LC |
| Hyperoliidae | *Hyperolius sylvaticus* | Nigeria | Imasuen et al. (2011) | LC |
| Hyperoliidae | *Hyperolius tuberilinguis* | Kenya | Kielgast et al. (2009) | LC |
| Hyperoliidae | *Hyperolius viridiflavus* | Kenya | Kielgast et al. (2009) | LC |
| Hyperoliidae | *Kassina senegalensis* | Kenya, Tanzania, South Africa | Weldon (2005); Moyer and Weldon (2006); Kielgast et al. (2009) | LC |
| Leiopelmatidae | *Ascaphus truei* | United States | Hossack et al. (2010) | LC |
| Leiopelmatidae | *Leiopelma archeyi* | New Zealand | Bell et al. (2004); A. Haigh, unpub. | CR |
| Leiuperidae^[[8]](#footnote-8)^ | *Engystomops (Physalaemus) pustulosus* | Panama, Venezuela | Lips et al. (2006); Sánchez et al. (2008) | LC |
| Leiuperidae | *Physalaemus henselii* | Uruguay | Borteiro et al. (2009) | LC |
| Leiuperidae | *Physalaemus (Engystomops) petersi* | Ecuador | McCracken et al. (2009) | LC |
| Leiuperidae | *Pleurodema bibroni* | Uruguay | Bardier et al. (2011) | NT |
| Leiuperidae | *Pleurodema marmorata (marmoratum)* | Chile, Peru | Seimon et al. (2007); R. Solis, unpub. | LC |
| Leiuperidae | *Pleurodema thaul* | Chile | Soto-Azat and Cunningham (2010) | LC |
| Leptodactylidae | *Leptodactylus albilabris* | Puerto Rico | Burrowes et al. (2008); P Burrowes, A. Longo, & R. Joglar, unpub. | LC |
| Leptodactylidae | *Leptodactylus discodactylus* | Ecuador | McCracken et al. (2009) | LC |
| Leptodactylidae | *Leptodactylus fallax* | Dominica, Montserrat | Malhotra et al. (2007); M. C. Fisher, unpub. ;  Durrell Wildlife Conservation Trust, unpub. | CR |
| Leptodactylidae | *Leptodactylus gracilis* | Argentina | Ghirardi et al. (2009) | LC |
| Leptodactylidae | *Leptodactylus ocellatus* | Argentina | Herrera et al. (2005); Ghirardi et al. (2009) | LC |
| Leptodactylidae | *Leptodactylus pentadactylus* | Japan (cap.), Panama, Ecuador | Lips et al. (2006); Une et al. (2008); McCracken et al. (2009) | LC |
| Leptodactylidae | *Leptodactylus rhodomystax* | Ecuador | McCracken et al. (2009) | LC |
| Leptodactylidae | *Leptodactylus* spp. | Venezuela | Lampo et al. (2006) |  |
| Limnodynastidae | *Adelotus brevis* | Australia | Berger et al. (1999); Speare and Berger (2005); Kriger and Hero (2007a); Murray et al. (2009); K. Murray, unpub. | NT |
| Limnodynastidae | *Heleioporus australiacus* | Australia | Berger et al. (1999) | VU |
| Limnodynastidae | *Heleioporus barycragus* | Australia | K. Murray, unpub. | LC |
| Limnodynastidae | *Heleioporus eyrei* | Australia | Berger et al. (1999); Speare and Berger (2005); K. Murray, unpub. | LC |
| Limnodynastidae | *Lechriodus fletcheri* | Australia | Berger et al. (1999) | LC |
| Limnodynastidae | *Limnodynastes dorsalis* | Australia | Berger et al. (1999); K. Murray, unpub. | LC |
| Limnodynastidae | *Limnodynastes dumerilii* | Australia | Berger et al. (1998, 1999); K. Murray, unpub. | LC |
| Limnodynastidae | *Limnodynastes peronii* | Australia | Kriger and Hero (2007a); K. Murray, unpub. | LC |
| Limnodynastidae | *Limnodynastes tasmaniensis* | Australia | Berger et al. (1998, 1999); Simpkins et al. (2010); K. Murray, unpub. | LC |
| Limnodynastidae | *Limnodynastes terraereginae* | Australia | K. Murray, unpub. | LC |
| Limnodynastidae | *Neobatrachus kunapalari* | Australia | Berger et al. (1999) | LC |
| Limnodynastidae | *Neobatrachus pelobatoides* | Australia | K. Murray, unpub. | LC |
| Megophryidae | *Leptobrachium hasseltii* | Indonesia | Kusrini et al. (2008) | LC |
| Microhylidae | *Cophixalus ornatus* | Australia | Kriger and Hero (2007b) | LC |
| Microhylidae | *Nelsonophryne aterrima* | Panama | Lips et al. (2006) | LC |
| Microhylidae | *Phrynomantis bifasciatus* | Tanzania | Moyer and Weldon (2006) | LC |
| Microhylidae | *Plethodontohyla tuberata* | Japan (cap.) | Une et al. (2008) | VU |
| Myobatrachidae | *Assa darlingtoni* | Australia | Kriger and Hero (2007a) | LC |
| Myobatrachidae | *Crinia georgiana* | Australia | Speare and Berger (2005); K. Murray, unpub. | LC |
| Myobatrachidae | *Crinia glauerti* | Australia | K. Murray, unpub. | LC |
| Myobatrachidae | *Crinia insignifera* | Australia | K. Murray, unpub. | LC |
| Myobatrachidae | *Crinia pseudinsignifera* | Australia | Berger et al. (1999); K. Murray, unpub. | LC |
| Myobatrachidae | *Crinia signifera* | Australia | Pauza et al. (2010) | LC |
| Myobatrachidae | *Crinia subinsignifera* | Australia | K. Murray, unpub. | LC |
| Myobatrachidae | *Crinia tasmaniensis* | Australia | Pauza et al. (2010) | LC |
| Myobatrachidae | *Geocrinia rosea* | Australia | Speare and Berger (2005); K. Murray, unpub. | LC |
| Myobatrachidae | *Geocrinia vitellina* | Australia | K. Murray, unpub. | VU |
| Myobatrachidae | *Mixophyes fasciolatus* | Australia | Berger et al. (1998, 1999); Symonds et al. (2007); K. Murray, unpub. | LC |
| Myobatrachidae | *Mixophyes fleayi* | Australia | Berger et al. (1998); Symonds et al. (2007); K. Murray, unpub. | EN |
| Myobatrachidae | *Mixophyes iteratus* | Australia | K. Murray, unpub. | EN |
| Myobatrachidae | *Mixophyes* spp. | Australia | Berger et al. (1999); K. Murray, unpub. |  |
| Myobatrachidae | *Pseudophryne corroboree* | Australia | K. Murray, unpub. | CR |
| Myobatrachidae | *Pseudophryne pengilleyi* | Australia | Berger et al. (1999); K. Murray, unpub. | EN |
| Myobatrachidae | *Taudactylus acutirostris* | Australia | Berger et al. (1998, 1999) | CR |
| Myobatrachidae | *Taudactylus eungellensis* | Australia | Berger et al. (1998); Retallick et al. (2004); K. Murray, unpub. | CR |
| Myobatrachidae | *Uperoleia fusca* | Australia | Kriger and Hero (2007a) | LC |
| Myobatrachidae | *Uperoleia laevigata* | Australia | K. Murray, unpub. | LC |
| Pelobatidae | *Pelobates cultripes* | Spain | S. Walker, unpub. | NT |
| Pelobatidae | *Pelobates varaldii* | Morocco | El Mouden et al. (2011) | EN |
| Petropedetidae | *Petropedetes (Arthroleptides) yakusini* | Tanzania | Weldon and du Preez (2004); Moyer & Weldon (2006) | EN |
| Phrynobatrachidae^[[9]](#footnote-9)^ | *Phrynobatrachus acridoides* | Kenya | Kielgast et al. (2009) | LC |
| Phrynobatrachidae | *Phrynobatrachus calcaratus* | Nigeria | Imasuen et al. (2011) | LC |
| Phrynobatrachidae | *Phrynobatrachus liberiensis* | Nigeria | Imasuen et al. (2011) | NT |
| Phrynobatrachidae | *Phrynobatrachus natalensis* | South Africa | C. Weldon, unpub. | LC |
| Phrynobatrachidae | *Phrynobatrachus plicatus* | Nigeria | Imasuen et al. (2011) | LC |
| Pipidae | *Hymenochirus boettgeri* | Canada | Raverty and Reynolds (2001) | LC |
| Pipidae | *Silurana (Xenopus) tropicalis* | Ghana, Nigeria | Parker et al. (2002); Imasuen et al. (2011) | LC |
| Pipidae | *Xenopus borealis* | Kenya | Kielgast et al. (2009) | LC |
| Pipidae | *Xenopus fraseri* | Cameroon | Soto-Azat et al. (2009) | LC |
| Pipidae | *Xenopus gilli* | South Africa | Weldon (2005); Soto-Azat et al. (2009) | EN |
| Pipidae | *Xenopus laevis* | Botswana, Chile, Ghana, Japan (cap.), Malawi, South Africa | Weldon (2005); Speare and Berger (2005); Goka et al. (2009); Soto-Azat et al. (2009); Solís et al. (2010); J. Longcore, unpub.; C. Weldon, unpub. | LC |
| Pipidae | *Xenopus muelleri* | Swaziland | Weldon (2005) | LC |
| Pipidae | *Xenopus petersii* | Botswana | Weldon (2005) | LC |
| Pipidae | *Xenopus victorianus* | Kenya | Kielgast et al. (2009) | LC |
| Pipidae | *Xenopus wittei* | Uganda | Goldberg et al. (2007) | LC |
| Ptychadenidae | *Ptychadena aequiplicata* | Tanzania | Weldon and du Preez (2004) | LC |
| Ptychadenidae | *Ptychadena anchietae* | Kenya, Tanzania | Berger and Speare (2000); Weldon and du Preez (2004); Speare and Berger (2005); Kielgast et al. (2009) | LC |
| Ptychadenidae | *Ptychadena longirostris* | Nigeria | Imasuen et al. (2011) | LC |
| Ptychadenidae | *Ptychadena mascareniensis* | Kenya, Uganda | Goldberg et al. (2007); Rödder et al. (2009) | LC |
| Ptychadenidae | *Ptychadena pumilio* | Nigeria | Imasuen et al. (2011) | LC |
| Pyxicephalidae | *Amietia (Afrana) angolensis* | Kenya, South Africa | Weldon (2005); Kielgast et al. (2009); C. Weldon, unpub. | LC |
| Pyxicephalidae | *Amietia (Afrana) dracomontana* | Lesotho, South Africa | Weldon (2005) | LC |
| Pyxicephalidae | *Amietia (Afrana) fuscigula* | South Africa | Hopkins and Channing (2003); Lane et al. (2003); Weldon (2005); C. Weldon, unpub. | LC |
| Pyxicephalidae | *Amietia vertebralis* | Lesotho | Weldon (2005) | LC |
| Pyxicephalidae | *Amietia wittei* | Kenya | Kielgast et al. (2009) | DD |
| Pyxicephalidae^[[10]](#footnote-10)^ | *Cacosternum boettgeri* | South Africa | Weldon (2005); C. Weldon, unpub. | LC |
| Pyxicephalidae | *Strongylopus fasciatus* | South Africa | Weldon (2005) | LC |
| Pyxicephalidae | *Strongylopus grayii* | South Africa | Hopkins and Channing (2003); C. Weldon, unpub. | LC |
| Pyxicephalidae | *Strongylopus hymenopus* | Lesotho, South Africa | Smith et al. (2007); M.-O. Roedel, unpub. | LC |
| Pyxicephalidae | *Tomopterna cryptotis* | South Africa | Weldon (2005) | LC |
| Pyxicephalidae | *Tomopterna natalensis* | South Africa | Weldon (2005); C. Weldon, unpub. | LC |
| Ranidae | *Babina (Rana) pleuraden* | China | Bai et al. (2010) | LC |
| Ranidae | *Glandirana (Rana) rugosa* | Japan | Goka et al. (2009) | LC |
| Ranidae | *Hylarana (Amnirana) albolabris* | Nigeria | Imasuen et al. (2011) | LC |
| Ranidae | *Hylarana (Rana) chalconota* | Indonesia | Kusrini et al. (2008) | LC |
| Ranidae | *Hylarana (Rana) luzonensis* | Philippines | A. Diesmos and R. Brown, unpub.^6^ | NT |
| Ranidae | *Hylarana (Rana) similis* | Philippines | A. Diesmos and R. Brown, unpub.^6^ | NT |
| Ranidae | *Lithobates (Rana) berlandieri* | United States | Sredl and Caldwell (2000); Lovich et al. (2008); Arizona Game & Fish Dept, unpub. | LC |
| Ranidae | *Lithobates (Rana) blairi* | United States | Sredl and Caldwell (2000); Carey and Livo (2009); Arizona Game & Fish Dept, unpub. | LC |
| Ranidae | *Lithobates catesbeianus*  *(Rana catesbeiana)* | Brazil, Canada, China, France, Italy, Japan, Rep. of Korea, United Kingdom, United States, Uruguay, Venezuela | Rosen and Schwalbe (2002); Sredl et al. (2002); Mazzoni et al. (2003); Morehouse et al. (2003); Hanselmann et al. (2004); Cunningham et al. (2005); Daszak et al. (2005); Ouellet et al. (2005); Pearl and Green (2005); Bettaso and Rachowicz (2006); Charbonneau (2006); Garner et al. (2006); Govindarajulu et al. (2006); Green and Dodd (2007); Longcore et al. (2007); Morgan et al. (2007); Pearl et al. (2007); Schlaepfer et al. (2007); Adams et al. (2008); Grant et al. (2008); Rothermel et al. (2008); Sánchez et al. (2008); Goka et al. (2009); Rizkalla (2009);Yang et al. (2009); Adams et al. (2010); Bai et al. (2010); Rimer and Briggler (2010); Rizkalla (2010); Sadinski et al. (2010); Saenz et al. (2010); Tupper et al. (2011); Also unpublished data from: M. Byrne; R. Fisher; T. Garner; D. E. Green; M. Hayes;V. Hemingway; G Padgett-Flohr; J Longcore; J. Kolby; J. Krebs; J. Mendelson.; P. Rosen and D. Caldwell.; J. Ware and K. Duncan; Arizona Game & Fish dept., unpub.; Canadian Coop. Wildlife Health Centre, unpub.; http://www.promedmail.org (Y. Ume) | LC |
| Ranidae | *Lithobates (Rana) chiricahuensis* | United States | Morell (1999); Bradley et al. (2002); Rosen and Schwalbe (2002); Sredl et al. (2002); Arizona Game & Fish Dept, unpub.; D. E. Green, unpub.; P. C. Rosen and D. Caldwell, unpub. | VU |
| Ranidae | *Lithobates (Rana) clamitans* | Canada, United States | Ouellet et al. (2005); Longcore et al. (2007); Byrne et al. (2008); Grant et al. (2008); Rothermel et al. (2008); St-Amour et al. (2008); Timpe et al. (2008); Groner and Relyea (2010); Monsen-Collar et al. (2010); Sadinski et al. (2010); Tupper et al. (2011); D. E. Green, unpub. | LC |
| Ranidae | *Lithobates (Rana) heckscheri* | United States | D. E. Green, unpub. | LC |
| Ranidae | *Lithobates maculatus (Rana maculata)* | El Salvador, Honduras | Puschendorf et al. (2006b); Felger et al. (2007); Kolby et al. (2010) | LC |
| Ranidae | *Lithobates (Rana) magnaocularis* | Mexico | Hale et al. (2005) | LC |
| Ranidae | *Lithobates (Rana) megapoda* | Mexico | Frías-Alvarez et al. (2008) | VU |
| Ranidae | *Lithobates (Rana) montezumae* | Mexico | Frías-Alvarez et al. (2008) | LC |
| Ranidae | *Lithobates neovolcanicus (Rana neovolcanica)* | Mexico | Frías-Alvarez et al. (2008) | NT |
| Ranidae | *Lithobates (Rana) palustris* | Canada, United States | Ouellet et al. (2005); Longcore et al. (2007); Grant et al. (2008); Rothermel et al. (2008); Timpe et al. (2008);Todd-Thompson et al. (2009); Rimer and Briggler (2010) | LC |
| Ranidae | *Lithobates (Rana) pipiens* | Canada, United States | Carey et al. (1999); Muths et al. (2003); Ouellet et al. (2005); Longcore et al. (2007); Young et al. (2007); Loda and Otis (2009); Rodriguez et al. (2009); Sadinski et al. (2010); Canadian Cooperative Wildlife Health Centre, unpub.; D. E. Green, unpub.; K. Kendell, unpub.; J. Longcore, unpub.; B. Maxell, unpub.; Northern Leopard Frog Recovery Team, unpub.; A. Pessier, unpub.; S. Wagner and J. Johnson, unpub. | LC |
| Ranidae | *Lithobates pustulosus (Rana pustulosa)* | Mexico | Hale et al. (2005) | LC |
| Ranidae | *Lithobates (Rana) septentrionalis* | Canada, United States | Ouellet et al. (2005); Longcore et al. (2007); Rodriguez et al. (2009); Sadinski et al. (2010); D. E. Green, unpub. | LC |
| Ranidae | *Lithobates sevosus (Rana sevosa)* | United States | Drake et al. (2007) | CR |
| Ranidae | *Lithobates (Rana) spectabilis* | Mexico | Frías-Alvarez et al. (2008); D. E. Green, unpub. | LC |
| Ranidae | *Lithobates sphenocephalus (Rana sphenocephala)* | United States | Mitchell and Green (2002); Daszak et al. (2005); Drake et al. (2007); Timpe et al. (2008); Venesky and Brem (2008); Rothermel et al. (2008); D. E. Green, unpub.; J. Ware and K. Duncan, unpub. | LC |
| Ranidae | *Lithobates (Rana) subaquavocalis^[[11]](#footnote-11)^* | United States | Sredl et al. (2002); Arizona Game & Fish Dept, unpub. | CR |
| Ranidae | *Rana sylvatica (Lithobates sylvaticus)* | Canada, United States | Rittmann et al. (2003); Muths et al. (2003); Green and Muths (2005); Ouellet et al. (2005); Longcore et al. (2007); Reeves and Green (2006); Young et al. (2007); Reeves (2008); Rothermel et al. (2008); Zellmer et al. (2008); Chatfield et al. (2009); Rodriguez et al. (2009); Schock et al. (2009); Slough (2009); Sadinski et al. (2010); D. E. Green, unpub.; J. Longcore, unpub. | LC |
| Ranidae | *Lithobates (Rana) tarahumarae* | Mexico, United States | Hale et al. (2005); Arizona Game & Fish Dept, unpub.; T. Jones, unpub. | VU |
| Ranidae | *Lithobates vibicarius (Rana vibicaria)* | Costa Rica | Puschendorf et al. (2006a, 2009) | CR |
| Ranidae | *Lithobates (Rana) warszewitschii* | Panama | Lips et al. (2006) | LC |
| Ranidae | *Lithobates (Rana) yavapaiensis* | Mexico, United States | Bradley et al. (2002); Rosen and Schwalbe (2002); Sredl et al. (2002); Morehouse et al. (2003); Hale et al. (2005); Schlaepfer et al. (2007); Arizona Game & Fish Dept, unpub.; D. E. Green, unpub.; T. Jones, unpub. | LC |
| Ranidae | *Odorrana andersonii* | China | Bai et al. (2010) | LC |
| Ranidae | *Odorrana (Rana) narina (*briefly *Eburana)* | Japan | Goka et al. (2009) | EN |
| Ranidae | *Pelophylax kl. esculentus (Rana esculenta)* | Denmark, Germany, Italy, Switzerland | Adams et al. (2008); Scalera et al. (2008); A. Hettyey, unpub; U. Reyer, unpub. | LC |
| Ranidae | *Pelophylax (Rana) lessonae* | Switzerland, Italy | Garner et al. (2005); Simoncelli et al. (2005); Di Rosa et al. (2007) | LC |
| Ranidae | *Pelophylax nigromaculatus (Rana nigromaculata)* | Japan | Goka et al. (2009) | NT |
| Ranidae | *Pelophylax (Rana) perezi* | Spain | S. Walker et al., unpub. | LC |
| Ranidae | *Pelophylax porosus porosus (Rana porosa porosa)* | Japan | Goka et al. (2009) | LC |
| Ranidae | *Pelophylax ridibundus (Rana ridibunda)* | Switzerland | Garner et al. (2005) | LC |
| Ranidae | *Rana arvalis* | Germany | Mutschmann et al. (2000) | LC |
| Ranidae | *Rana aurora* | Canada, United States | Pearl et al. (2007); Adams et al. (2007, 2010); D. E. Green, unpub.; N. Nieto, unpub. | LC |
| Ranidae | *Rana boylii* | United States | Lowe (2009); Adams et al. (2010); P. Johnson, unpub.; S. Kupferberg, unpub; G. Padgett-Flohr, unpub. | NT |
| Ranidae | *Rana cascadae* | United States | Adams et al. (2010); K. Pope, unpub.; S. Wagner and J. Johnson, unpub. | NT |
| Ranidae | *Rana chaochiaoensis* | China | Bai et al. (2010) | LC |
| Ranidae | *Rana draytonii* | United States  (US Threatened) | Morgan et al. (2007); Adams et al. (2010); Tatarian and Tatarian (2010); R. Fisher, unpub.; V. Hemingway, unpub.; T. James, unpub.; P. Johnson, unpub.; G. Padgett-Flohr, unpub. | VU |
| Ranidae | *Rana iberica* | Spain | S. Walker, unpub. | NT |
| Ranidae | *Rana latastei* | Italy | Garner et al. (2005) | VU |
| Ranidae | *Rana luteiventris* | United States | Muths et al. (2003, 2008); Pearl et al. (2007); Adams et al. (2010); E Bull, unpub.; C. Goldberg and L. Watts, unpub.; D. E. Green, unpub.; J. Lowe, unpub.; B. Maxell, unpub.; D. Pilliod and E. Muths, unpub. | LC |
| Ranidae | *Rana muscosa* | United States | Fellers et al. (2001); Morehouse et al. (2003); Morgan et al. (2007); Woodhams et al. (2007); R. Fisher, unpub.; D. E. Green, unpub.; R. Knapp, unpub.; D. McGriff, unpub.; S. Muskopf, unpub.; G. Padgett-Flohr, unpub. | EN |
| Ranidae | *Rana ornativentris* | Japan (cap.) | Goka et al. (2009) | LC |
| Ranidae | *Rana pretiosa* | United States | Pearl et al. (2007, 2009); Hayes et al. (2009); Adams et al. (2010); D. E. Green, unpub. | VU |
| Ranidae | *Rana pyrenaica* | Spain | S. Walker, unpub. | EN |
| Ranidae | *Rana sierrae* | United States | Briggs et al. (2010); Morgan et al. (2007); R. Knapp, unpub. | EN |
| Ranidae | *Rana tagoi* | Japan | S. Okada, unpub. | LC |
| Ranidae | *Rana temporaria* | Denmark, France, Spain, Hungary | Garner et al. (2005); Scalera et al. (2008); S. Walker, unpub. | LC |
| Rhacophoridae | *Buergeria buergeri* | Japan | S. Okada, unpub. | LC |
| Rhacophoridae | *Buergeria japonica* | Japan | Goka et al. (2009) | LC |
| Rhacophoridae | *Chiromantis rufescens* | Nigeria | Imasuen et al. (2011) | LC |
| Rhacophoridae | *Rhacophorus margaritifer (javanus)* | Indonesia | Kusrini et al. (2008) | LC |
| Rhacophoridae | *Rhacophorus viridis viridis* | Japan | S. Okada, unpub. | LC |
| Scaphiopodidae | *Scaphiopus holbrookii* | United States | Tupper et al. (2011) | LC |
| Strabomantidae^[[12]](#footnote-12)^ | *Bryophryne cophites* | Peru | Catenazzi et al. (2011) | EN |
| Strabomantidae | *Bryophryne zonalis* | Peru | Catenazzi et al. (2011) | NE |
| Strabomantidae | *Noblella* sp. | Peru | Catenazzi et al. (2011) | — |
| Strabomantidae | *Pristimantis aureolineatus* | Ecuador | McCracken et al. (2009) | LC |
| Strabomantidae | *Pristimantis (Eleutherodactylus) caryophyllaceus* | Panama | Morehouse et al. (2003); Lips et al. (2006) | NT |
| Strabomantidae | *Pristimantis cerasinus (*was *Craugastor)* | Panama | Lips et al. (2006) | LC |
| Strabomantidae | *Pristimantis (Eleutherodactylus) chrysops* | Colombia | Velásquez-E. et al. (2008) | EN |
| Strabomantidae | *Pristimantis (Eleutherodactylus) cruentus* | Panama | Berger et al. (1998); Lips et al. (2006) | LC |
| Strabomantidae | *Pristimantis danae* | Peru | Catenazzi et al. (2011) | LC |
| Strabomantidae | *Pristimantis (Eleutherodactylus) elegans* | Colombia | Ruiz and Rueda-Almonacid (2008) | VU |
| Strabomantidae | *Pristimantis (Eleutherodactylus) erythropleura* | Colombia | Velásquez-E. et al. (2008) | LC |
| Strabomantidae | *Pristimantis (Eleutherodactylus) fenestratus* | Peru | I. De la Riva, unpub. | LC |
| Strabomantidae | *Pristimantis (Eleutherodactylus) gracilis* | Colombia | Velásquez-E. et al. (2008) | VU |
| Strabomantidae | *Pristimantis lanthanites* | Ecuador | McCracken et al. (2009) | LC |
| Strabomantidae | *Pristimantis (Eleutherodactylus) museosus* | Panama | Lips et al. (2006) | EN |
| Strabomantidae | *Pristimantis ockendeni* | Ecuador | McCracken et al. (2009) | LC |
| Strabomantidae | *Pristimantis (Eleutherodactylus) palmeri* | Colombia | Velásquez-E. et al. (2008) | LC |
| Strabomantidae | *Pristimantis pharangobates (rhabdolaemus)* | Peru | Catenazzi et al. (2011) | LC |
| Strabomantidae | *Pristimantis platydactylus* | Peru | Catenazzi et al. (2011) | LC |
| Strabomantidae | *Pristimantis (Eleutherodactylus) ridens* | Panama | Lips et al. (2006) | LC |
| Strabomantidae | *Pristimantis salaputium* | Peru | Catenazzi et al. (2011) | DD |
| Strabomantidae | *Pristimantis (Eleutherodactylus) silverstonei* | Colombia | Velásquez-E. et al. (2008) | NT |
| Strabomantidae | *Pristimantis (Eleutherodactylus) thectopternus* | Colombia | Velásquez-E. et al. (2008) | LC |
| Strabomantidae | *Pristimantis toftae* | Peru | Catenazzi et al. (2011) | LC |
| Strabomantidae | *Pristimantis (Eleutherodactylus) w-nigrum* | Colombia | Velásquez-E. et al. (2008) | LC |
| Strabomantidae | *Pristimantis waoranii* | Ecuador | McCracken et al. (2009) | DD |
| Strabomantidae | *Psychrophrynella sp. nov.* | Peru | Catenazzi et al. (2011) | NE |
| Strabomantidae | *Psychrophrynella usurpator* | Peru | Catenazzi et al. (2011) | EN |
| Strabomantidae | *Strabomantis (Craugastor) bufoniformis* | Panama | Lips et al. (2006) | LC |
| Strabomantidae | *Strabomantis (Eleutherodactylus) ruizi* | Colombia | Velásquez-E. et al. (2008) | EN |

**Part 2. Caudata**

| **Family** | **Species** | **Country** | **Reference** | **Status** |
| --- | --- | --- | --- | --- |
| Ambystomatidae | *Ambystoma altamirani* | Mexico | Frías-Alvarez et al. (2008) | EN |
| Ambystomatidae | *Ambystoma californiense* | United States | Padgett-Flohr and Longcore (2005) | VU |
| Ambystomatidae | *Ambystoma gracile* | United States | S. Wagner and J. Johnson, unpub. | LC |
| Ambystomatidae | *Ambystoma granulosum* | Mexico | Frías-Alvarez et al. (2008) | CR |
| Ambystomatidae | *Ambystoma jeffersonianum* | United States | Brodman and Briggler (2008) | LC |
| Ambystomatidae | *Ambystoma macrodactylum* | United States | S. Wagner and J. Johnson, unpub; D. E. Green, unpub.; C. Goldberg and L. Watts, unpub. | LC |
| Ambystomatidae | *Ambystoma maculatum* | Australia (cap.), Canada | Ouellet et al. (2005) | LC |
| Ambystomatidae | *Ambystoma mexicanum* (cap.) | Australia | Berger et al. (1999) | CR |
| Ambystomatidae | *Ambystoma rivulare* | Mexico | Frías-Alvarez et al. (2008) | DD |
| Ambystomatidae | *Ambystoma tigrinum* | Canada, United States | Davidson et al. (2003); Morehouse et al. (2003); Muths et al. (2008); Sredl et al. (2002); D. E. Green, unpub.; B. Maxell, unpub.; Canadian Coop. Wildlife Health Centre, unpub. | LC |
| Ambystomatidae | *Ambystoma velasci* | Mexico | Frías-Alvarez et al. (2008) | LC |
| Ambystomatidae^[[13]](#footnote-13)^ | *Dicamptodon aterrimus* | United States | D. E. Green, unpub. | LC |
| Ambystomatidae^11^ | *Dicamptodon tenebrosus* | United States | S. Kupferberg, unpub. | LC |
| Cryptobranchidae | *Andrias japonicus* | Japan | Goka et al. (2009) | NT |
| Cryptobranchidae | *Cryptobranchus alleganiensis alleganiensis* | United States | Briggler et al. (2008);  Gonyor et al. (2011); G. Lipps, unpub. | NT |
| Cryptobranchidae | *Cryptobranchus alleganiensis bishopi* | United States | Briggler et al. (2008) | NT |
| Plethodontidae | *Batrachoseps attenuatus* | United States | Weinstein (2009) | LC |
| Plethodontidae | *Batrachoseps gavilanensis* | United States | Weinstein (2009) | LC |
| Plethodontidae | *Batrachoseps nigriventris* | United States | Weinstein (2009) | LC |
| Plethodontidae | *Batrachoseps relictus* | United States | Weinstein (2009) | DD |
| Plethodontidae | *Batrachoseps wrighti* | United States | Weinstein (2009) | VU |
| Plethodontidae | *Bolitoglossa colonnea* | Panama | Lips et al. (2006) | LC |
| Plethodontidae | *Bolitoglossa lincolni* | Guatemala | Rovito et al. (2009) | NT |
| Plethodontidae | *Bolitoglossa occidentalis* | Guatemala | Rovito et al. (2009) | LC |
| Plethodontidae | *Bolitoglossa schizodactyla* | Panama | Lips et al. (2006) | LC |
| Plethodontidae | *Dendrotriton bromeliacius* | Guatemala | Rovito et al. (2009) | CR |
| Plethodontidae | *Desmognathus conanti* | United States | Timpe et al. (2008) | NE |
| Plethodontidae | *Desmognathus fuscus* | United States | Grant et al. (2008); Hossack et al. (2010) | LC |
| Plethodontidae | *Desmognathus monticola* | United States | Hossack et al. (2010) | LC |
| Plethodontidae | *Eurycea bislineata* | United States | Grant et al. (2008); D. E. Green, unpub. | LC |
| Plethodontidae | *Eurycea cirrigera* | United States | Byrne et al. 2008 | LC |
| Plethodontidae | *Eurycea longicauda* | United States | Rimer and Briggler (2010) | LC |
| Plethodontidae | *Eurycea nana* | United States | Gaertner et al. (2007, 2009a) | VU |
| Plethodontidae | *Eurycea neotenes* | United States | Gaertner et al. (2009a) | VU |
| Plethodontidae | *Eurycea pterophila* | United States | Gaertner et al. (2009a) | DD |
| Plethodontidae | *Eurycea quadridigitata* | United States | Saenz et al. (2010) | LC |
| Plethodontidae | *Eurycea sosorum* | United States | Gaertner et al. (2007, 2009a) | VU |
| Plethodontidae | *Eurycea spelaea* | United States | Rimer and Briggler (2010) | LC |
| Plethodontidae | *Eurycea tonkawae* | United States | Gaertner et al. (2007, 2009a) | EN |
| Plethodontidae | *Oedipina collaris* | Panama | Lips et al. (2006) | DD |
| Plethodontidae | *Plethodon cinereus* | United States | Lauer et al. (2007) | LC |
| Plethodontidae | *Plethodon neomexicanus* | United States | Cummer et al. (2005) | NT |
| Plethodontidae | *Pseudotriton ruber* | United States | Speare and Berger (2005); Montanucci (2009) | LC |
| Proteidae | *Necturus maculosus* | United States | D. E. Green, unpub. | LC |
| Salamandridae | *Calotriton (Euproctus) asper^[[14]](#footnote-14)^* | Spain | S. Walker, unpub. | NT |
| Salamandridae | *Cynops ensicauda* | Japan | Goka et al. (2009); S. Okada, unpub. | EN |
| Salamandridae | *Cynops pyrrhogaster* | Japan | S. Okada, unpub. | LC |
| Salamandridae | *Euproctus platycephalus* | Italy | Bovero et al. (2008) ; M. C. Fisher, unpub. | EN |
| Salamandridae | *Ichthyosaura (Triturus, Mesotriton) alpestris^[[15]](#footnote-15)^* | Switzerland, Spain | Garner et al. (2005); S. Walker, unpub. | LC |
| Salamandridae | *Lissotriton (Triturus) helveticus^[[16]](#footnote-16)^* | France, Spain | S. Walker, unpub. | LC |
| Salamandridae | *Lissotriton (Triturus) vulgaris^[[17]](#footnote-17)^* | United Kingdom | Garner et al. (2005) | LC |
| Salamandridae | *Notophthalmus viridescens*  *(N. v. viridescens; N. v. dorsalis)* | Canada, United States | Ouellet et al. (2005); Rothermel et al. (2008); Zippel and Tabaka (2008); Chatfield et al. (2009); Bakkegard and Pessier (2010); Groner and Relyea (2010); D. E. Green, unpub.; M. Whitney, unpub. | LC |
| Salamandridae | *Pleurodeles waltl* | Spain, Portugal | J. Bosch, unpub.; M. C. Fisher, unpub.; S. Walker, unpub. | NT |
| Salamandridae | *Salamandra salamandra* | France, Spain | Bosch and Martinez-Solano (2006); S. Walker, unpub. | LC |
| Salamandridae | *Taricha granulosa* | United States | S. Kupferberg, unpub. | LC |
| Salamandridae | *Taricha torosa* | United States | Padgett-Flohr and Longcore (2007); R. Knapp, unpub. | LC |
| Salamandridae | *Triturus marmoratus* | Spain | S. Walker, unpub. | LC |
| Salamandridae | *Triturus pygmaeus* | Spain | S. Walker, unpub. | NT |
| Sirenidae | *Siren intermedia* | United States | Talley et al. (2011) | LC |

1. Formerly family Discoglossidae, before Frost et al. (2006). [↑](#footnote-ref-1)
2. Based on the location of the record, this is not *Nectophrynoides asperginis*, which has (or perhaps had) an extremely restricted range—the spray zone of a single waterfall in the Kihansi Gorge of the Udzungwa Mountains of Tanzania. [↑](#footnote-ref-2)
3. The family Craugastoridae is one of three subdivided by Hedges et al. (2008) from the family Brachycephalidae as assigned by Frost et al. (2006). All species in the genus *Craugastor* were formerly known as *Eleutherodactylus*, and in several cases were reported as such in their first occurrence with *Bd*. [↑](#footnote-ref-3)
4. The three species of *Hyloxalus* shown were formerly placed in the genus *Colostethus* (Frost [2008]). [↑](#footnote-ref-4)
5. The species name for *Hyloxalus fascianiger* was corrected to *Hyloxalus fascianigrus* in Frost (2007). [↑](#footnote-ref-5)
6. Reported at www.google.com/hostednews/afp/article/ALeqM5iqFIa4AFOylJaFUUECJw0z0-99Rg [↑](#footnote-ref-6)
7. The family Eleutherodactylidae is one of three subdivided by Hedges et al. (2008) from the family Brachycephalidae as assigned by Frost et al. (2006). All species now in the genus *Diasporus* were formerly known as *Eleutherodactylus*, and were reported as such in their first occurrence with *Bd*. [↑](#footnote-ref-7)
8. Formerly placed in the family Leptodactylidae. [↑](#footnote-ref-8)
9. Formerly placed in the family Petropedetidae. [↑](#footnote-ref-9)
10. Formerly placed in the family Ranidae. [↑](#footnote-ref-10)
11. Now treated as a (possibly extinct) subspecies of *Rana (Lithobates) chiricahuensis*. [↑](#footnote-ref-11)
12. The family Strabomantidae is one of three subdivided by Hedges et al. (2008) from the family Brachycephalidae as assigned by Frost et al. (2006). All species now in the genera *Pristimantis* and *Strabomantis* were formerly known as *Eleutherodactylus*, and many were reported as such in their first occurrence with *Bd*. [↑](#footnote-ref-12)
13. Formerly family Dicamptodontidae. [↑](#footnote-ref-13)
14. Recently moved from the genus *Euproctus*, based on a new understanding of population and genetic relationships (Frost 2009). [↑](#footnote-ref-14)
15. Reported with *Bd* as *Triturus alpestris*, briefly *Mesotriton alpestris*, now (according to Frost [2009]) *Ichthyosaura alpestris*. [↑](#footnote-ref-15)
16. Reported with *Bd* as *Triturus helveticus*; recently reclassified as *Lissotriton* (Frost 2009). [↑](#footnote-ref-16)
17. Reported with *Bd* as *Triturus vulgaris*; recently reclassified as *Lissotriton* (Frost 2009). [↑](#footnote-ref-17)
