# Supplementary material for: Mapping the Global Emergence of Batrachochytrium dendrobatidis, the Amphibian Chytrid Fungus
Source: PLoS One. 2013 Feb 27;8(2):e56802. doi: 10.1371/journal.pone.0056802 (PMC3584086; doi:10.1371/journal.pone.0056802)
Supplement: Table S2 — Parameters used in regression analyses. Numbers in parentheses after biomes indicate number of sites in the full analysis (N = 3733 total sites). Sites with no biome assigned (n = 227) were dropped from the analysis. Lake biome (N = 1 site) was also dropped due to inconsistency of lentic habitat designation. (DOCX) [file pone.0056802.s007.docx]

**Table S2:**  Parameters used in regression analyses. Numbers in parentheses after biomes indicate number of sites in the full analysis (N = 3733 total sites). Sites with no biome assigned (n = 227) were dropped from the analysis. Lake biome (N = 1 site) was also dropped due to inconsistency of lentic habitat designation.

| **Parameters In Regression Models** | |
| --- | --- |
| Latitude |  |
| Elevation (m) |  |
| Climate metrics |  |
|  | Minimum annual temperature (°C) |
|  | Maximum annual temperature (°C) |
|  | Annual temperature range (°C) |
|  | Average annual temperature (°C) |
|  | Average annual precipitation (mm) |
| Species Richness |  |
| Biomes |  |
|  | 1: Tropical & Subtropical Moist Broadleaf Forests (452) |
|  | 2: Tropical & Subtropical Dry Broadleaf Forests (23) |
|  | 3: Tropical & Subtropical Coniferous Forests (54) |
|  | 4: Temperate Broadleaf & Mixed Forests (801) |
|  | 5: Temperate Coniferous Forests (892) |
|  | 6: Boreal Forests/Taiga (35) |
|  | 7: Tropical & Subtropical Grasslands (140) |
|  | 8: Temperate Grasslands, Savannas & Shrublands (206) |
|  | 9: Flooded Grasslands & Savannas (11) |
|  | 10: Montane Grasslands & Shrublands (56) |
|  | 11: Tundra (36) |
|  | 12: Mediterranean Forests, Woodlands & Scrub (828) |
|  | 13: Deserts & Xeric Shrublands (193) |
|  | 14: Mangroves (6) |
