# Supplementary material for: Mapping the Global Emergence of Batrachochytrium dendrobatidis, the Amphibian Chytrid Fungus
Source: PLoS One. 2013 Feb 27;8(2):e56802. doi: 10.1371/journal.pone.0056802 (PMC3584086; doi:10.1371/journal.pone.0056802)
Supplement: Table S3 — Significant parameters in three family-scale logistic regression models using Batrachochytrium dendrobatidis ( Bd ) occurrence data from wild-occurring amphibians having exact and approximate locations in the global Bd database. Individual biome coefficients represent additive shifts in the Bd-occurrence odds ratio, whereas other coefficients represent multiplicative changes in odds of detecting Bd. Logistic regression with likelihood ratio test statistic (Chi2) was used. (DOCX) [file pone.0056802.s008.docx]

**Table S3:** Significant parameters in three family-scale logistic regression models using *Batrachochytrium dendrobatidis* (*Bd*) occurrence data from wild-occurring amphibians having exact and approximate locations in the global *Bd* database. Individual biome coefficients represent additive shifts in the *Bd*-occurrence odds ratio, whereas other coefficients represent multiplicative changes in odds of detecting *Bd*. Logistic regression with likelihood ratio test statistic (Chi^2^) was used.

**Part 1: Bufonidae (N = 885 sites)**

| **Parameter** | **Coefficient** | **SE** | **P value** |
| --- | --- | --- | --- |
| Intercept | 2.445 | 1.846 | -- |
| Elevation | 0.0006 | 0.0001 | 0.004 |
| Minimum Temperature | 0.232 | 0.050 | 0.005 |
| Average Temperature | -0.250 | 0.065 | 0.00008 |
| **Biome** | -- | -- | <0.00001 |
| Tropical & Subtropical Moist Broadleaf Forests | Included in intercept |  |  |
| Montane Grasslands & Shrublands | 3.103 | 4.772 |  |
| Tundra | 0.126 | 1.635 |  |
| Mediterranean Forest, Woodlands & Scrub | -0.708 | 0.802 |  |
| Deserts & Xeric Shrublands | -0.198 | 0.486 |  |
| Tropical & Subtropical Dry Broadleaf Forests | -0.364 | 0.382 |  |
| Tropical & Subtropical Coniferous Forests | 0.710 | 1.942 |  |
| Temperate Broadleaf & Mixed Forests | -0.048 | 0.309 |  |
| Temperate Conifer Forests | -0.099 | 0.234 |  |
| Boreal Forests/Taiga | -0.583 | 1.364 |  |
| Tropical & Subtropical Grasslands | -0.132 | 0.214 |  |
| Temperate Grasslands, Savannas & Shrublands | -0.004 | 0.167 |  |
| Flooded Grasslands & Savannas | -0.155 | 0.165 |  |

**Part 2: Hylidae (N = 1179 sites)**

| **Parameter** | **Coefficient** | **SE** | **P value** |
| --- | --- | --- | --- |
| Intercept | 9.763 | 1.895 | -- |
| Latitude | -0.055 | 0.010 | <0.00001 |
| Minimum Temperature | -0.108 | 0.039 | <0.002 |
| Maximum Temperature | -0.0523 | 0.064 | 0.0004 |
| Average Temperature Range | -0.538 | 0.102 | <0.00001 |
| **Biome** | -- | -- | <0.00001 |
| Tropical & Subtropical Moist Broadleaf Forests | Included in intercept |  |  |
| Montane Grasslands & Shrublands | 1.557 | 3.196 |  |
| Mediterranean Forest, Woodlands & Scrub | -0.043 | 1.079 |  |
| Deserts & Xeric Shrublands | 0.308 | 0.559 |  |
| Tropical & Subtropical Dry Broadleaf Forests | 0.186 | 0.417 |  |
| Tropical & Subtropical Coniferous Forests | 0.027 | 0.246 |  |
| Temperate Broadleaf & Mixed Forests | -0.215 | 0.171 |  |
| Temperate Conifer Forests | 0.040 | 0.134 |  |
| Tropical & Subtropical Grasslands | -0.252 | 0.172 |  |
| Temperate Grasslands, Savannas & Shrublands | -0.016 | 0.104 |  |

**Part 3: Ranidae (N = 866 sites).**

| **Parameter** | **Coefficient** | **SE** | **P value** |
| --- | --- | --- | --- |
| Intercept | -0.786 | 0.439 |  |
| **Biome** | -- | -- | <0.00001 |
| Tropical & Subtropical Moist Broadleaf Forests | Included in intercept |  |  |
| Montane Grasslands & Shrublands | NA |  |  |
| Tundra | -3.163 | 2.285 |  |
| Mediterranean Forest, Woodlands & Scrub | 0.916 | 0.764 |  |
| Deserts & Xeric Shrublands | 1.094 | 0.388 |  |
| Tropical & Subtropical Dry Broadleaf Forests | 0.267 | 0.269 |  |
| Tropical & Subtropical Coniferous Forests | 0.361 | 0.169 |  |
| Temperate Broadleaf & Mixed Forests | 0.272 | 0.114 |  |
| Temperate Conifer Forests | 0.174 | 0.085 |  |
| Boreal Forests/Taiga | -0.061 | 0.089 |  |
| Tropical & Subtropical Grasslands | 0.240 | 0.123 |  |
| Temperate Grasslands, Savannas & Shrublands | -0.011 | 0.050 |  |
