# Supplementary material for: Mapping the Global Emergence of Batrachochytrium dendrobatidis, the Amphibian Chytrid Fungus
Source: PLoS One. 2013 Feb 27;8(2):e56802. doi: 10.1371/journal.pone.0056802 (PMC3584086; doi:10.1371/journal.pone.0056802)
Supplement: File S1 — Supplemental Information: Taxonomic Notes; References for Supplemental Material. (DOCX) [file pone.0056802.s009.docx]

**Mapping the Global Emergence of *Batrachochytrium dendrobatidis*, the Amphibian Chytrid Fungus**

Deanna H. Olson, David M. Aanensen, Kathryn L. Ronnenberg, Christopher I. Powell, Susan F. Walker, Jon Bielby, Trenton W. J. Garner, George Weaver, the *Bd* Mapping Group, and Matthew C. Fisher

**Supplemental Information**

**Taxonomic Notes**

Genera were assigned to families for summarization (Table 1 in main text) and analysis (Table 2 in main text) based on the most recent available comprehensive taxonomic references (Frost et al. 2006, Frost 2008, Frost 2009, Frost 2011). We chose recent family designations to explore patterns of *Bd* susceptibility and occurrence because these classifications were based on both genetic and morphological data, and hence may more likely yield meaningful inference. Some North American species were assigned to genus according to Crother (2008), and dendrobatid frogs were assigned to family and genus based on Grant et al. (2006). Eleutherodactylid frogs were assigned to family and genus based on Hedges et al. (2008); centrolenid frogs based on Cisneros-Heredia et al. (2007). For the eleutherodactylid frogs of Central and South America and the Caribbean, older sources count them among the Leptodactylidae, whereas Frost et al. (2006) put them in the family Brachycephalidae. More recent work (Heinicke et al. 2007) suggests that most of the genera that were once “*Eleutherodactylus*” (including those species currently assigned to the genera *Eleutherodactylus*, *Craugastor*, *Euhyas*, *Phrynopus*, and *Pristimantis* and assorted others), may belong in a separate, or even several different new families. Subsequent work (Hedges et al. 2008) has divided them among three families, the Craugastoridae, the Eleutherodactylidae, and the Strabomantidae, which were used in our classification.

In cases where *Bd*-positive individuals were reported under both the more recent and the previous genus, we were careful to count them as only one species for our tallies. Species are referred to by their current binomial (as given in the IUCN Red List, IUCN 2010), both here and in the automatic synonymy of the online database. Where more recent classification has changed, that is noted in Table S1, and the *Bd* Maps database contains an automatic synonymy. Spelling was standardized according to Frost (2011).

**References for Supplemental Text, Figures, and Tables**

Adams MJ, Galvan S, Reinitz D, Cole RA, Pyare S, et al. (2007) Incidence of the fungus *Batrachochytrium dendrobatidis* in amphibian populations along the northwest coast of North America. Herpetol Rev 38: 430-431.

Adams MJ, Galvan S, Scalera R, Grieco C, Sindaco R (2008) *Batrachochytrium dendrobatidis* in amphibian populations in Italy. Herpetol Rev 39: 324-326.

Adams MJ, Chelgren ND, Reinitz D, Cole RA, Rachowicz LJ, et al. (2010) Using occupancy models to understand the distribution of an amphibian pathogen, *Batrachochytrium dendrobatidis*. Ecol Appl 20: 289-302.

Alemu I JB, Cazabon MNE, Dempewolf L, Hailey A, Lehtinen RM, et al. (2008) Presence of the chytrid fungus *Batrachochytrium dendrobatidis* in populations of the critically endangered frog *Mannophryne olmonae* in Tobago, West Indies. EcoHealth 5: 34-39.

Bai C, Garner TWJ, Li Y (2010) First evidence of *Batrachochytrium dendrobatidis* in China: Discovery of chytridiomycosis in introduced American bullfrogs and native amphibians in the Yunnan Province, China. EcoHealth 7: 127-134. Doi: 10.1007/s10393-010-0307-0

Bakkegard KA, Pessier AP (2010) *Batrachochytrium dendrobatidis* in adult *Notophthalmus viridescens* in north-central Alabama, USA. Herpetol Rev 41: 45-47.

Bardier C, Ghirardi R, Levy M, Maneyro R (2011) First case of chytridiomycosis in an adult specimen of a native anuran from Uruguay. Herpetol Rev 42: 65-66.

Barrasso DA, Cajade R, Nenda SJ, Baloriani G, Herrera R (2009) Introduction of the American bullfrog *Lithobates catesbeianus* (Anura: Ranidae) in natural and modified environments: an increasing conservation problem in Argentina. S Amer J Herpetol 4, 69-75.

Barrionuevo S, Mangione S (2006) Chytridiomycosis in two species of *Telmatobius* (Anura: Leptodactylidae) from Argentina. Dis Aquat Org 73: 171-174.

Barrionuevo JS, Aguayo R, Lavilla EO (2008) First record of chytridiomycosis in Bolivia (*Rhinella quechua*; Anura: Bufonidae). Dis Aquat Org 82: 161-163.

Beard KH, O’Neill EM (2005) Infection of an invasive frog *Eleutherodactylus coqui* by the chytrid fungus *Batrachochytrium dendrobatidis* in Hawaii. Biolog Conserv 126: 591-595.

Bell BD, Carver S, Mitchell NJ, Pledger S (2004) The recent decline of a New Zealand endemic: how and why did populations of Archey’s frog *Leiopelma archeyi* crash over 1996-2001? Biolog Conserv 120: 189-199.

Berger L, Speare R, Daszak P, Green DE, Cunningham AA, et al. (1998) Chytridiomycosis causes amphibian mortality associated with population declines in the rain forests of Australia and Central America. Proc Natl Acad Sci U S A 95: 9031-9036.

Berger L, Speare R, Hyatt AD (1999) Chytrid fungi and amphibian declines: overview, implications and future directions. In: Campbell A (ed.) Declines and disappearances of Australian frogs*.* Environment Australia, Canberra, pp 23-33.

Bettaso JB, Rachowicz LJ (2006) Overwintering larvae of bullfrogs (*Rana catesbeiana*) as biological reservoirs of the pathogen causing chytridiomycosis (*Batrachochytrium dendrobatidis*) in Trinity County, California. Northwest Nat 87, 162.

Bielby J, Bovero S, Sotgiu G, Tessa G, Favelli M, et al. (2009) Fatal chytridiomycosis in the Tyrrhenian painted frog. EcoHealth 6: 27-32. doi: 10.1007/s10393-009-0232-2

Blackburn DC, Evans BJ, Pessier AP, Vredenburg VT (2010) An enigmatic mortality event in the only population of the Critically Endangered Cameroonian frog *Xenopus longipes*. Afr J Herpetol 2010: 1-12 iFirst, Doi: 10.1080/04416651.2010.495674

Blackburn LM (2001) Status of Blanchard’s cricket frogs (*Acris crepitansblanchardi*) along their decline front: population parameters, malformation rates and disease. Muncie, Indiana, USA: Ball State University. Master’s thesis.

Bonaccorso E, Guyasamin JM, Méndez D, Speare R (2003) Chytridiomycosis as a possible cause of population declines in *Atelopus cruciger* (Anura: Bufonidae). Herpetol Rev 34: 331-334.

Borteiro C, Cruz JC, Kolenc F, Aramburu A (2009) Chytridiomycosis in frogs from Uruguay. Dis Aquat Org 84: 159-162.

Bosch J, Martínez-Solano I (2006) Chytrid fungus infection related to unusual mortalities of *Salamandra salamandra* and *Bufo bufo* in the Peñalara Natural Park, Spain. Oryx 40: 84-89.

Bosch J, Martínez-Solano I, García-París M (2001) Evidence of a chytrid fungus infection involved in the decline of the common Midwife Toad (*Alytes obstricans*) in protected areas of central Spain. Biol Conserv 97: 331-337.

Bourke J, Mutschmann F, Ohst T, Ulmer P, Gutsche A, et al. (2010) *Batrachochytrium dendrobatidis* in Darwin’s frog *Rhinoderma* spp. in Chile. Dis Aquat Org 92: 217-221. doi: 10.3354/dao02239 (2010).

Bovero S, Sotgiu G, Angelini C, Doglio S, Gazzaniga E, et al. (2008) Detection of chytridiomycosis caused by *Batrachochytrium dendrobatidis* in the endangered Sardinian brook newt *Euproctus platycephalus* in southern Sardinia, Italy. J Wildl Dis 44: 712-715.

Bradley GA, Rosen PC, Sredl MJ, Jones TR, Longcore JE (2002) Chytridiomycosis in native Arizona frogs. J Wildl Dis 38: 206-212.

Briggler JT, Larson KA, Irwin KJ (2008) Presence of the amphibian chytrid fungus (*Batrachochytrium dendrobatidis*) on hellbenders (*Cryptobranchus alleganiensis*) in the Ozark Highlands. Herpetol Rev 39: 443-444.

Briggs C, Burgin S (2004) Congo Red, an effective stain for revealing the chytrid fungus, *Batrachochytrium dendrobatidis*, in epidermal skin scrapings from frogs. Mycologist 18: 98-103.

Briggs CJ, Knapp RA, Vredenburg VT (2010) Enzootic and epizootic dynamics of the chytrid fungal pathogen of amphibians. Proc Natl Acad Sci USA 107: 9695-9700.

Brodman R, Briggler JT (2008) *Batrachochytrium dendrobatidis* in *Ambystoma jeffersonianum* larvae in southern Indiana. Herpetol Rev 39: 320-321.

Burrowes PA, Longo AV, Joglar RL, Cunningham AA (2008) Geographic distribution of *Batrachochytrium dendrobatidis* in Puerto Rico. Herpetol Rev 39: 321-324.

Byrne MW, Davie EP, Gibbons JW (2008) *Batrachochytrium dendrobatidis* occurrence in *Eurycea cirrigera*. Southeastern Naturalist 7: 551-555.

Carey C, Livo LJ (2009) Chytridiomycosis in Woodhouse’s Toad (*Anaxyrus woodhousii*) in Colorado. Herpetol Rev 40: 50-52.

Carey C, Cohen N, Rollins-Smith LA (1999) Amphibian declines: An immunological perspective. Dev Comp Immun 23: 459-472.

Carnaval ACOQ, Toledo LF, Haddad CFB, Britto FB (2005) Chytrid fungus infects high-altitude stream-dwelling *Hylodes magalhaesi* (Leptodactylidae) in the Brazilian Atlantic rainforest. FrogLog 70: 3-4.

Catenazzi A, Vredenburg VT, Lehr E (2010) *Batrachochytrium dendrobatidis* in the live frog trade of *Telmatobius* (Anura: Ceratophryidae) in the tropical Andes. Dis Aquat Org 92: 187-191. doi:10.3354/dao02250 (2010a).

Catenazzi A, Lehr E, Rodriguez LO, Vredenburg VT (2011) *Batrachochytrium dendrobatidis* and the collapse of anuran species richness in the upper Manu National Park, southeastern Peru. Conserv Biol 25: 382-391. doi: 10.1111/j.1523-1739.2010.01604.x

Charbonneau M (2006) Amphibian diseases: Pesticide immunotoxicity and chytridiomycosis in larval *Rana catesbeiana* and ranaviral disease in *Rana sylvatica* tadpoles of central Ontario. MSc thesis, Trent University, Peterborough, Ontario, Canada.

Chatfield MWH, Rothermel BB, Brooks CS, Kay JB (2009) Detection of *Batrachochytrium dendrobatidis* in amphibians from the Great Smoky Mountains of North Carolina and Tennessee, USA. Herpetol Rev 40: 176-179.

Chestnut T, Johnson JE, Wagner RS (2008) Results of amphibian chytrid (*Batrachochytrium dendrobatidis*) sampling in Denali National Park, Alaska, USA. Herpetol Rev 39: 202-204.

Cisneros-Heredia DF, McDiarmid RW (2007) Revision of the characters of Centrolenidae (Amphibia: Anura: Athesphatanura), with comments on its taxonomy and the description of new taxa of glassfrogs. Zootaxa 1572.

Crother BI (2008) Scientific and standard English names of amphibians and reptiles of North America north of Mexico, with comments regarding confidence in our understanding. Soc Study Amph Rept Herpetol Circ 37 (2008).

Cummer MR, Green DE, O’Neill EM (2005) Aquatic chytrid pathogen detected in a terrestrial Plethodontid salamander. Herpetol. Rev. 36, 248-249.

Cunningham AA, Garner TWJ, Aguilar-Sanchez V, Banks B, Foster J, et al. (2005) Emergence of amphibian chytridiomycosis in Britain. Vet. Rec. 157, 386-387.

Daszak P, Strieby A, Cunningham AA, Longcore JE, Brown CC, et al. (2005) Amphibian population declines at Savannah River site are linked to climate, not chytridiomycosis. Ecology 86: 3232-3237.

Daversa D, Bosch J, Jeffery K (2011) First survey of the chytrid fungus, *Batrachochytrium dendrobatidis*, in amphibian populations of Gabon, Africa. Herpetol Rev 42: 67-69.

Davidson EW, Parris M, Collins JP, Longcore JE, Pessier AP et al. (2003) Pathogenicity and transmission of chytridiomycosis in Tiger Salamanders (*Ambystoma tigrinum*). Copeia 2003: 601-607.

Deguise I, Richardson JS (2009) Prevalence of the chytrid fungus (*Batrachochytrium dendrobatidis*) in Western Toads in southwestern British Columbia, Canada. Northwestern Nat 90: 35-38.

Di Leo K (2010) An assessment of the correlation between amphibian populations, chytridiomycete communities, and the ecological integrity of the habitat. Masters thesis, Ecology and Evolution. Rutgers University, Camden, NJ.

Di Rosa I, Simoncelli F, Fagotti A, Pascolini R (2007)The proximate cause of frog declines? Nature 447: E4-E5.

Díaz L, Cádiz A, Chong A, Silva A (2007) First report of chytridiomycosis in a dying toad (Anura: Bufonidae) from Cuba: A new conservation challenge for the island. EcoHealth 4: 172-175.

Doherty-Bone TM, Bielby J, Gonwouo NL, LeBreton M, Cunningham AA (2008) In a vulnerable position? Preliminary survey work fails to detect the amphibian chytrid pathogen in the highlands of Cameroon, an amphibian hotspot. Herpetol J 18: 115-118.

Drake DL, Altig R, Grace JB, Walls SG (2007) Occurrence of oral deformities in larval anurans. Copeia 2007: 449-458.

El Mouden EH, Slimani T, Donaire D, Fernández-Beaskoetxea S, Fisher MC, et al. (2011) First record of the chytrid fungus *Batrachochytrium dendrobatidis* in North Africa. Herpetol Rev 42: 71-75.

Enciso MA, Villena M, Mendoza AP, Chávez G (2008) Rapid survey of amphibian skin diseases in a mountain forest at the northern Andes of Peru. FrogLog 87: 4-7.

Felger J, Enssle J, Mendez D, Speare R (2007) Chytridiomycosis in El Salvador. Salamandra 43: 122-127.

Fellers GM, Green DE, Longcore JE (2001) Oral chytridiomycosis in the Mountain Yellow-legged Frog (*Rana muscosa*). Copeia 2001: 945-953.

Ferreira TK, Lamarão FRM, Moraes MO, Van Sluys M (2008) Amphibian chytrid infection in *Melanophryniscus moreirae* (Bufonidae) in the Brazilian Atlantic Rainforest. Herpetol Rev 39: 445-446.

Fox SF, Greer AL, Torres-Cervantes R, Collins JP (2006) First case of ranavirus-associated morbidity and mortality in natural populations of a South American frog, *Atelognathus patagonicus*. Dis Aquat Org 72: 87-92.

Frías-Alvarez P, Vredenburg VT, Familiar-López M, Longcore JE, González-Bernal E et al. (2008) Chytridiomycosis survey in wild and captive Mexican amphibians. EcoHealth 5: 18-26.

Frost DR (2008) Amphibian species of the world: an online reference. Version 5.2 (15 July, 2008). Electronic database accessible at http://research.amnh.org/herpetology/amphibia/index.php American Museum of Natural History, New York, USA.

Frost DR (2009) Amphibian species of the world: an online reference. Version 5.3 (12 February, 2009). Electronic database accessible at http://research.amnh.org/herpetology/amphibia/index.php American Museum of Natural History, New York, USA.

Frost DR (2010) Amphibian species of the world: an online reference. Version 5.4 (8 April, 2010). Electronic database accessible at http://research.amnh.org/vz/herpetology/amphibia/

American Museum of Natural History, New York, USA.

Frost DR (2011) Amphibian species of the world: an online reference. Version 5.5 (31 January, 2011). Electronic database accessible at http://research.amnh.org/vz/herpetology/amphibia/

American Museum of Natural History, New York, USA.

Frost DR, Grant T, Faivovich J, Bain RH, Haas A et al. (2006) The amphibian tree of life. Bull Amer Mus Nat Hist 297.

Gaertner JP, Hahn D, Forstner MRJ (2007) Detection of *Batrachochytrium dendrobatidis* in four endemic Central Texas amphibians. Abstracts of Nov. 2007 *Bd* Conference, Available at: http://www.parcplace.org/documents/Bd_Program_post-FINAL.pdf.

Gaertner JP, Gaston MA, Spontak D, Forstner MRJ, Hahn D (2009a) Seasonal variation in the detection of *Batrachochytrium dendrobatidis* in a Texas population of Blanchard’s Cricket Frog (*Acris crepitans blanchardi*). Herpetol Rev 40: 184-187.

Gaertner JP, Forstner MRJ, O’Donnell L, Hahn D (2009b) Detection of *Batrachochytrium dendrobatidis* in endemic salamander species from Central Texas. EcoHealth 6: 20-26. doi: 10.1007/s10393-009-0229-x

Gaertner JP, McHenry D, Forstner MRJ, Hahn D (2010) Annual variation of *Batrachochytrium dendrobatidis* in the Houston Toad (*Bufo houstonensis*) and a sympatric congener (*Bufo nebulifer*). Herpetol Rev 41: 456-459.

Garcia G, Cunningham AA, Horton DL, Garner TWJ, Hyatt A et al. (2007) Mountain chickens *Leptodactylus fallax* and sympatric amphibians appear to be disease free on Montserrat. Oryx 41: 398-401.

Garner TWJ, Walker S, Bosch J, Hyatt AD, Cunningham AA et al. (2005) Chytrid fungus in Europe. Emerg Inf Dis 11: 1639-1640.

Garner TWJ, Perkins MW, Govindarajulu P, Seglie D, Walker S et al. (2006) The emerging amphibian pathogen *Batrachochytrium dendrobatidis* globally infects introduced populations of the North American bullfrog, *Rana catesbeiana*. Biol Lett 2: 455-459.

Ghirardi R, Lescano JN, Longo MS, Robledo G, Steciow MM et al. (2009) *Batrachochytrium dendrobatidis* in Argentina: First record in *Leptodactylus gracilis* and another record in *Leptodactylus ocellatus*. Herpetol Rev 40: 175-176.

Glenney GW, Julian JT, Quartz WM (2010) Preliminary amphibian health survey in the Delaware Water Gap National Recreation Area. J Aquat Anim Health 22: 102-114. Doi: 10.1577/H09-037.1

Goka K, Yokoyama J, Une Y, Kuroki T, Suzuki K et al. (2009) Amphibian chytridiomycosis in Japan: distribution, haplotypes and possible route of entry into Japan. Molec Ecol 18: 4757-4774.

Goldberg CS, Hawley TJ, Waits LP (2009) Local and regional patterns of amphibian chytrid prevalence on the Osa Peninsula, Costa Rica. Herpetol Rev 40: 309-311.

Goldberg TL, Readel AM, Lee MH (2007) Chytrid fungus in frogs from an equatorial African montane forest in western Uganda. J Wildl Dis 43: 521-524.

Gonyor JL, Yabsley MJ, Jensen JB (2011) A preliminary survey of *Batrachochytrium dendrobatidis* exposure in hellbenders from a stream in Georgia. Herpetol Rev 42: 58-59.

Govindarajulu PP, Garner TWJ, Anholt BR (2006) Assessing prevalence of chytrid fungus (*Batrachochytrium dendrobatidis*) in native amphibians and bullfrogs (*Rana catesbeiana*) on Vancouver Island, British Columbia. Northwest Nat 87: 170.

Grant EHC, Bailey LL, Ware JL, Duncan KL (2008) Prevalence of the amphibian pathogen *Batrachochytrium dendrobatidis* in stream and wetland amphibians in Maryland, USA. Appl Herpetol 5: 233-241.

Grant T, Frost DR, Caldwell JP, Gagliardo R, Haddad CFB et al. (2006) Phylogenetic systematics of dart-poison frogs and their relatives (Amphibia: Athesphatanura: Dendrobatidae). Bull Amer Mus Nat Hist 299.

Gratwicke B, Alonso A, Elie T, Kolowski J, Lock J et al. (2011) *Batrachochytrium dendrobatidis* not detected on amphibians from two lowland sites in Gabon, Africa. Herpetol Rev 42: 69-71.

Green DE, Dodd CK (2007) Presence of amphibian chytrid fungus *Batrachochytrium dendrobatidis* and other amphibian pathogens at warm-water fish hatcheries in southeastern North America. Herpetol Conserv Biol 2: 43-47.

Green DE, Muths E (2005) Health evaluation of amphibians in and near Rocky Mountain National Park (Colorado, USA). Alytes 22: 109-129.

Greenbaum E, Kusamba C, Aristote MM, Reed K (2008) Amphibian chytrid fungus infections in *Hyperolius* (Anura: Hyperoliidae) from eastern Democratic Republic of Congo. Herpetol Rev 39: 70-73.

Groner ML, Relyea RA (2010) *Batrachochytrium dendrobatidis* is present in northwest Pennsylvania, USA, with high prevalence in *Notophthalmus viridescens*. Herpetol Rev 41: 462-465.

Gutierrez FR, Arellano ML, Moreno LE, Natale GS (2010) *Batrachochytrium dendrobatidis* in Argentina: First record of infection in *Hypsiboas cordobae* and *Odontophrynus occidentalis* tadpoles, in San Luis Province. Herpetol Rev 41: 323-325.

Hale SF, Rosen PC, Jarchow JL, Bradley GA (2005) Effects of chytrid fungus on the Tarahumara Frog (*Rana tarahumarae*) in Arizona and Sonora, Mexico. In: Gottfried GJ, Gebow BS, Eskew LG, Edminster CB (compilers) Connecting mountain islands and desert seas: biodiversity and management of the Madrean Archipelago II. 2004 May 11-15; Tucson, AZ. Proceedings RMRS-P-36. Fort Collins, CO: U.S. Department of Agriculture, Forest Service, Rocky Mountain Research Station. pp. 407-411.

Hanselmann R, Rodríguez A, Lampo M, Fajardo-Ramos L, Aguirre AA, et al. (2004) Presence of an emerging pathogen of amphibians in introduced bullfrogs *Rana catesbeian*a in Venezuela. Biol Conserv 120: 115-119.

Hasken J, Newby JL, Grelle AM, Boling J, Estes J et al. (2009) Evaluation of chytrid infection level in a newly discovered population of *Anaxyrus boreas* in the Rio Grande National Forest, Colorado, USA. Herpetol Rev 40: 426-428.

Hayes MP, Rombough CJ, Padgett-Flohr GE, Hallock LA, Johnson JE et al. (2009) Amphibian chytridiomycosis in the Oregon Spotted Frog (*Rana pretiosa*) in Washington state, USA. Northwestern Naturalist 90: 148-151.

Hedges SB, Duellman WE, Heinicke MP (2008) New World direct-developing frogs (Anura: Terrarana): Molecular phylogeny, classification, biogeography, and conservation. Zootaxa 1737: 1-182.

Heinicke MP, Duellman WE, Hedges SB (2007) Major Caribbean and Central American frog faunas originated by ancient oceanic dispersal. Proc Nat Acad Sci U S A 104: 10092-10097.

Herrera RA, Seciow MM, Natale GS (2005) Chytrid fungus parasitizing the wild amphibian *Leptodactylus ocellatus* (Anura: Leptodactylidae) in Argentina. Dis Aquat Org 64: 247-252.

Hopkins S, Channing A (2003) Chytrid fungus in Northern and Western Cape frog populations, South Africa. Herpetol Rev 34: 334-336.

Hossack BR, Adams MJ, Campbell Grant EH, Pearl CA ,Bettaso JB et al. (2010) Low prevalence of chytrid fungus (*Batrachochytrium dendrobatidis*) in amphibians in U. S. headwater streams. J Herpetol 44: 253-260.

Imasuen AA, Aisien MSO, Weldon C, Dalton DL, Kotze A et al. (2011) Occurrence of *Batrachochytrium dendrobatidis* in amphibian populations of Okomu National Park, Nigeria. Herpetol Rev 42: 379-382.

IUCN (2010) IUCN Red List of Threatened Species. Version 2010.1. Available at: http//www.iucnredlist.org. Downloaded on 15 April 2010.

Joglar RL, Álvarez AO, Aide TM, Barber D, Burrowes PA et al. (2007) Conserving the Puerto Rican herpetofauna. Appl Herpetol 4: 327-345.

Kaiser K, Grafe TU (2011) Chytrid fungus not found in preliminary survey of lowland amphibian populations across northwestern Borneo. Herpetol Rev 42: 59-61.

Kielgast J, Rödder D, Veith M, Lötters S (2009) Widespread occurrence of the amphibian chytrid fungus in Kenya. Animal Conserv 13, suppl. 1: 1-8.

Kolby JE, Padgett-Flohr GE (2009) Reassessment of the historical timeline for *Batrachochytrium dendrobatidis* presence in Honduras and conservation implications for *Plectrohyla dasypus*. Herpetol Rev 40: 307-308.

Kolby JE, Padgett-Flohr GE, Field R (2010) Amphibian chytrid fungus *Batrachochytrium dendrobatidis* in Cusuco National Park, Honduras. Dis Aquat Org 92: 245-251. doi: 10.3354/dao02055

Kriger KM, Hero J-M (2006) Survivorship in wild frogs infected with chytridiomycosis. EcoHealth 3: 171-177.

Kriger KM, Hero J-M (2007a) The chytrid fungus *Batrachochytrium dendrobatidis* is non-randomly distributed across amphibian breeding habitats. Diversity Distrib 13: 781-788.

Kriger KM, Hero J-M (2007b) *Cophixalus ornatus* (Ornate Nursery Frog). Chytridiomycosis. Herpetol Rev 38: 321.

Kriger KM, Pereoglou F, Hero J-M (2007) Latitudinal variation in the prevalence and intensity of chytrid (*Batrachochytrium dendrobatidis*) infection in eastern Australia. Conserv Biol 21: 1280-1290.

Kusrini MD, Skerratt LF, Garland S, Berger L, Endarwin W (2008) Chytridiomycosis in frogs of Mount Gede Pangrango, Indonesia. Dis Aquat Org 82: 187-194.

Lampo M, Señaris JC (2006) Unexplained amphibian mortalities in the secluded mountains of the Venezuelan Guayana: is there evidence of chytridiomycosis? Herpetol Rev 37: 47-49.

Lampo M, Rodríguez A, La Marca E, Daszak PA (2006a) chytridiomycosis epidemic and a severe dry season precede the disappearance of *Atelopus* species from the Venezuelan Andes. Herpetol J 16: 395-402.

Lampo M, Barrio-Amorós CL, Han BA (2006b) *Batrachochytrium dendrobatidis* infection in the recently rediscovered *Atelopus mucubajiensis* (Anura, Bufonidae), a critically endangered frog from the Venezuelan Andes. EcoHealth 3: 299-302.

Lampo M, Sánchez D, Nicolás A, Márquez M, Nava-González F, et al. (2008) *Batrachochytrium dendrobatidis* in Venezuela. Herpetol Rev 39: 449-454.

Lane EP, Weldon C, Bingham J (2003) Histological evidence of chytridiomycete fungal infection in a free-ranging amphibian, *Afrana fuscigula* (Anura: Ranidae) in South Africa. J S Afr Vet Assoc 74: 20-21.

Lauer A, Simon MA, Banning JL, André E, Duncan K, et al. Common cutaneous bacteria from the eastern red-backed salamander can inhibit pathogenic fungi. Copeia 2007: 630-640.

Lehtinen RM, Kam Y-C, Richards CL (2008) Preliminary surveys for *Batrachochytrium dendrobatidis* in Taiwan. Herpetol Rev 39: 317-318.

Lips KR, Green DE, Papendick R (2003) Chytridiomycosis in wild frogs from southern Costa Rica. J Herpetol 37: 215-218.

Lips KR, Mendelson JR III, Muñoz-Alonso A, Canseco-Márquez L, Mulcahy DG (2004) Amphibian population declines in montane southern Mexico: resurveys of historical localities. Biol Conserv 119: 555-564. Doi: 10.1016/j.biocon.2004.01.017

Lips KR, Brem F, Brenes R, Reeve JD, Alford RA et al. (2006) Emerging infectious disease and the loss of biodiversity in a Neotropical amphibian community. Proc Natl Acad Sci U S A 103: 3165-3170.

Loda JL, Otis DL (2009) Low prevalence of amphibian chytrid fungus (*Batrachochytrium dendrobatidis*) in northern leopard frog (*Rana pipiens*) populations of north-central Iowa, USA. Herpetol Rev 40: 428-431.

Longcore JR, Longcore JE, Pessier AP, Halteman WA (2007) Chytridiomycosis widespread in anurans of northeastern United States. J Wildl Manage 71: 435-444.

Longo AV, Burrowes PA, Joglar RL (2010) Seasonality of *Batrachochytrium dendrobatidis* infection in direct-developing frogs suggests a mechanism for persistence. Dis Aquat Org 92: 253-260. doi: 10.3354/dao02054.

Lovich R, Ryan MJ, Pessier AJ, Claypool B (2008) Infection with the fungus *Batrachochytrium dendrobatidis* in a non-native *Lithobates berlandieri* below sea level in the Coachella Valley, California, USA. Herpetol Rev 39: 315-317.

Lowe J (2009) Amphibian chytrid (*Batrachochytrium dendrobatidis*) in post-metamorphic *Rana boylii* in Inner Coast Ranges of central California. Herpetol Rev 40: 180-182.

Malhotra A, Thorpe RS, Hypolite E, James A (2007) A report on the status of the herpetofauna of the Commonwealth of Dominica, West Indies. Appl Herpetol 4: 177-194.

Mazzoni R, Cunningham AA, Daszak P, Apolo A, Perdomo E, et al. (2003) Emerging pathogen of wild amphibians in frogs (*Rana catesbeiana*) farmed for international trade. Emerg Inf Dis 9: 995-998.

McCracken S, Gaertner JP, Forstner MRJ, Hahn D (2009) Detection of *Batrachochytrium dendrobatidis* in amphibians from the forest floor to the upper canopy of an Ecuadorian Amazon lowland rainforest. Herpetol Rev 40: 190-195.

McLeod DS, Sheridan JA, Jiraungkoorskul W, Khonsue W (2008) A survey for chytrid fungus in Thai amphibians. Raffles Bull Zool 56: 199-204.

Mendelson JR III, Brodie ED Jr, Malone JH, Acevedo ME, Baker MA et al. (2004) Factors associated with the catastrophic decline of a cloudforest frog fauna in Guatemala. Int J Trop Biol 47: 217-223.

Mitchell JC, Green DE (2002) Chytridiomycosis in two species of ranid frogs in the southeastern United States. Joint Meeting of the American Society of Ichthyologists and Herpetologists, Herpetologists' League, and Society for the Study of Amphibians and Reptiles, 4-8 July 2002, Kansas City, USA.

Monsen-Collar K, Hazard L, Dussa R (2010) Comparison of PCR and RT-PCR in the first report of *Batrachochytrium dendrobatidis* in amphibians in New Jersey, USA. Herpetol Rev 41: 460-462.

Montanucci RR (2009) The chytrid fungus in the Red Salamander, *Pseudotriton ruber*, in South Carolina, USA. Herpetol Rev 40: 188.

Morehouse EA, James TY, Ganley ARD, Vilgalys R, Berger L, et al. (2003) Multilocus sequence typing suggests the chytrid pathogen of amphibians is a recently emerged clone. Mol Ecol 12: 395-403.

Morell V (1999) Are pathogens felling frogs? Science 284: 728-731.

Morgan JAT, Vredenburg VT, Rachowicz LJ, Knapp RA, Stice MJ et al. (2007) Population genetics of the frog killing fungus *Batrachochytrium dendrobatidis*. Proc Natl Acad Sci U S A 104: 13845-13850.

Moyer DC, Weldon C (2006) Chytrid distribution and pathogenicity among frogs of the Udzungwa Mountains, Tanzania. Report for the Wildlife Conservation Society, Bronx, NY, USA.

Murphy PJ, St-Hilaire S, Bruer S, Corn PS, Peterson CR (2009) Distribution and pathogenicity of *Batrachochytrium dendrobatidis* in boreal toads from the Grand Teton area of western Wyoming. EcoHealth 6: 109-120. doi: 10.1007/s10393-009-0230-4s.

Murray KA, Skerratt LF, Speare R, McCallum H (2009) Impact and dynamics of disease in species threatened by the amphibian chytrid fungus, *Batrachochytrium dendrobatidis*. Conserv Biol 23:1242-1252.

Muths E, Corn PS, Pessier AP, Green DE (2003) Evidence for disease-related amphibian decline in Colorado. Biol Conserv 110: 357-365.

Muths E, Pilliod DS, Livo LJ (2008) Distribution and environmental limitations of an amphibian pathogen in the Rocky Mountains, USA. Biol Conserv 141: 1484-1492.

Muths E, Spurre Pedersen B, Spurre Pedersen F (2009) How relevant is opportunistic *Bd* sampling: Are we ready for the big picture? Herpetol Rev 40: 183-184.

Mutschmann F, Berger L, Zwart P, Gaedicke C (2000) [Chytridiomycosis in amphibians—first report in Europe] Berl Munch Tierarztl Wochenschr 113: 380-383. [In German].

Nicolás A (2007) Tendencia altitudinal de infección en un hongo patógeno en ranas *Mannophryne herminae* del Parque Nacional Henri Pittier. Undergraduate thesis. Universidad Simón Bolívar, Caracas, Venezuela.

Obendorf DL (2005) Application of field and diagnostic methods for chytridiomycosis in Tasmanian frogs. Central North Field Naturalists, Inc., Tasmania, Australia.

Obendorf DL, Dalton A (2006) A survey for the presence of the amphibian chytrid fungus (*Batrachochytrium dendrobatidis*) in Tasmania. Pap Proc Royal Soc Tasmania 140: 25-29.

Ouellet M, Mikaelian I, Pauli BD, Rodrigue J, Green DE (2005) Historical evidence of wide-spread chytrid infection of North American amphibian populations. Conserv Biol 19: 1431-1440.

Padgett-Flohr GE, Longcore JE (2005) *Ambystoma californiense* (California Tiger Salamander). Fungal infection. Herpetol Rev 36: 50-51.

Padgett-Flohr GE, Longcore JE (2007) *Taricha torosa* (California Newt). Fungal infection. Herpetol Rev 38: 176-177.

Parker JM, Mikaelian I, Hahn N, Diggs HE (2002) Clinical diagnosis and treatment of epidermal chytridiomycosis in African clawed frogs (*Xenopus tropicalis*). Comp Med 52: 265–268.

Pauza M, Driessen M (2008) Distribution and potential spread of amphibian chytrid fungus *Batrachochytrium dendrobatidis* in the Tasmanian Wilderness World Heritage Area. Report to the Biodiversity Conservation Branch, Department of Primary Industries and Water, Tasmania. February 2008.

Pauza MD, Driessen MM, Skerratt LF (2010) Distribution and risk factors for spread of amphibian chytrid fungus *Batrachochytrium dendrobatidis* in the Tasmanian Wildnerness World Heritage Area, Australia. Dis Aquat Org 92: 193-199. doi: 10.3354/dao02212.

Pearl CA, Green DE (2005) *Rana catesbeiana* (American Bullfrog) Chytridiomycosis. Herpetol Rev 36: 305.

Pearl CA, Bull EL, Green DE, Bowerman J, Adams MJ, et al. (2007) Occurrence of the amphibian pathogen *Batrachochytrium dendrobatidis* in the Pacific Northwest. J Herpetol 41: 145-149.

Pearl CA, Bowerman J, Adams MJ, Chelgren ND (2009) Widespread occurrence of the chytrid fungus *Batrachochytrium dendrobatidis* on Oregon Spotted Frogs (*Rana pretiosa*). EcoHealth 6: 209-218 doi: 10.1007/s10393-009-0237-x

Pessier AP, Nichols DK, Longcore JE, Fuller MS (1999) Cutaneous chytridiomycosis in poison dart frogs (*Dendrobates* spp.) and White's tree frogs (*Litoria caerulea*). J Vet Diag Inv 11: 194-199.

Picco AM, Collins JP (2007) Fungal and viral pathogen occurrence in Costa Rican amphibians. J Herpetol 41: 746-749.

Pilliod DS, Muths E, Scherer RD, Bartelt PE, Corn PS, et al. (2010) Effects of amphibian chytrid fungus on individual survival probability in wild boreal toads. Conserv Biol 24: 1259-1267.

Puschendorf, R (2003) *Atelopus varius* (Harlequin Frog). Fungal infection. Herpetol Rev 34: 355.

Puschendorf R, Bolanos F, Chaves G. (2006a) The amphibian chytrid fungus along an altitudinal transect bore the first reported declines in Costa Rica. Biol Conserv 132: 136-142.

Puschendorf R, Castañeda F, McCranie J (2006b) Chytridiomycosis in wild frogs from Pico Bonito National Park, Honduras. EcoHealth 3: 178-181.

Puschendorf R, Carnaval AC, VanDerWal J, Zumbado-Ulate H, Chaves G et al. (2009) Distribution models for the amphibian chytrid *Batrachochytrium dendrobatidis* in Costa Rica: proposing climatic refuges as a conservation tool. Diversity Distrib 15: 401-408.

R Devlopment Core Team. (2009) R: A language and environment for statistical computing. Available from: http://www.R-project.org.

Raverty S, Reynolds T (2001) Cutaneous chytridiomycosis in dwarf aquatic frogs (*Hymenochirus boettgeri*) originating from southeast Asia and in a western toad (*Bufo boreas*) from northeastern British Columbia. Can Vet J 42: 385-386.

Reeves MK (2008) *Batrachochytrium dendrobatidis* in wood frogs (*Rana sylvatica*) from three National Wildlife Refuges in Alaska, USA. Herpetol Rev 39: 68-70.

Reeves MK, Green DE (2006) *Rana sylvatica* (Wood Frog). Chytridiomycosis. Herpetol Rev 37: 450.

Retallick RWR, McCallum H, Speare R (2004) Endemic infection of the amphibian chytrid fungus in a frog community post-decline. PloS Biology 2: 1965-1971.

Richards CL, Zellmer AJ, Martens LM (2008) *Batrachochytrium dendrobatidis* not detected in *Oophaga pumilio* on Bastimentos Island, Panama. Herpetol Rev 39: 200-202.

Rimer RL, Briggler JT (2010) Occurrence of the amphibian chytrid fungus (*Batrachochytrium dendrobatidis*) in Ozark caves, Missouri, USA. Herpetol Rev 41: 175-177.

Rittmann SE, Muths E, Green DE (2003) *Pseudacris triseriata* (Western Chorus Frog) and *Rana sylvatica* (Wood Frog). Chytridiomycosis. Herpetol Rev 34: 53.

Rizkalla CE (2009) First reported detection of *Batrachochytrium dendrobatidis* in Florida, USA. Herpetol Rev 40: 189-190.

Rizkalla CE (2010) Increasing detections of *Batrachochytrium dendrobatidis* in central Florida, USA. Herpetol Rev 41: 180-181.

Rodriguez EM, Gamble T, Hirt MV, Cotner S. (2009) Presence of *Batrachochytrium dendrobatidis* at the headwaters of the Mississippi River, Itasca State Park, Minnesota, USA. Herpetol Rev 40: 48-50.

Rogers KB, Banulis T (2004) Repatriation of boreal toads *Bufo boreas* on the Grand Mesa, Colorado, In Rogers KB (editor) Boreal Toad Research Report: 2003, pp. 2-12. Colorado Division of Wildlife, Fort Collins, CO, USA. Available from: http://wildlife.state.co.us/Research/Aquatic/BorealToad/, or http://wildlife.state.co.us/NR/rdonlyres/1013C627-855D-4AEC-9D74-278847451617/0/2003BUBOreport.pdf, accessed 24 September 2009.

Ron SR, Merino-Viteri A (2000) Amphibian declines in Ecuador: overview and first report of chytridiomycosis from South America. Froglog 42: 2-3.

Rosen PC, Schwalbe CR (2002) Final report to AGFD [Arizona Game and Fish Department] Heritage Program (IIPAM I99016), and USFWS [U.S. Fish and Wildlife Service].

Rothermel BB, Walls SC, Mitchell JC, Dodd CK, Irwin LK, et al. (2008) Widespread occurrence of the amphibian chytrid fungus (*Batrachochytrium dendrobatidis*) in the southeastern United States. Dis Aquat Org 82: 3-18.

Rovito SM, Parra-Olea G, Vásquez-Almazán CR, Papenfuss TJ, Wake DB (2009) Dramatic declines in neotropical salamander populations are an important part of the global crisis. Proc Natl Acad Sci U S A 106: 3231-3236.

Rowley JJL, Chan SKF, Tang WS, Speare R, Skerratt LF, et al. (2007a) Survey for the amphibian chytrid *Batrachochytrium dendrobatidis* in Hong Kong in native amphibians and in the international amphibian trade. Dis Aquat Org 78: 87-95. doi:10.3354/dao01861

Rowley JJL, Skerratt LF, Alford RA, Campbell R (2007b) Retreat sites of rain forest stream frogs are not a reservoir for *Batrachochytrium dendrobatidis* in northern Queensland, Australia. Dis Aquat Org 74: 7-12.

Ruiz A, Rueda-Almonacid JVR (2008) *Batrachochytrium dendrobatidis* and chytridiomycosis in anural amphibians of Colombia. EcoHealth 5: 27-33.

Sadinski W, Roth M, Treleven S, Theyerl J, Dummer P. (2010) Detection of the chytrid fungus, *Batrachochytrium dendrobatidis*, on recently metamorphosed amphibians in the north-central United States. Herpetol Rev 41: 170-175.

Saenz D, Adams CK, Pierce JB, Laurencio D (2009) Occurrence of *Batrachochytrium dendrobatidis* in an anuran community in the southeastern Talamanca region of Costa Rica. Herpetol Rev 40: 311-313.

Saenz D, Kavanagh BT, Kwiatkowski MA (2010) *Batrachochytrium dendrobatidis* detected in amphibians from national forests in eastern Texas, USA. Herpetol Rev 41: 47-79.

St-Amour V, Wong WM, Garner TWJ, Lesbarrères D (2008) Anthropogenic influence on the

prevalence of two amphibian pathogens. Emerg Inf Dis 14: 1175-1176.

Sánchez DA, Chacón-Ortiz A, León F, Han BA, Lampo M (2008) Rediscovery of *Atelopus cruciger* (Anura: Bufonidae) with notes on its current status in the Cordillera de La Costa, Venezuela. Biol Conserv 78: 87-95.

Scalera R, Adams MJ, Galvan SK (2008) The occurrence of *Batrachochytrium dendrobatidis* in amphibian populations in Denmark. Herpetol Rev 39: 199-200.

Schlaepfer MA, Sredl MJ, Rosen PC, Ryan MJ (2007) High prevalence of *Batrachochytrium dendrobatidis* in wild populations of lowland leopard frogs *Rana yavapaiensis* in Arizona. EcoHealth 4: 421-427.

Schock DM, Ruthig GR, Collins JP, Kutz SJ, Carrière S, et al. (2009) Amphibian chytrid fungus and ranaviruses in the Northwest Territories, Canada. Dis Aquat Org 92: 231-240. preprint doi: 10.3354/dao02134.

Seimon TA, Seimon A, Daszak P, Halloy SRP, Schloegel LM, et al. (2007) Upward range extension of Andean anurans and chytridiomycosis to extreme elevations in response to tropical deglaciation. Global Change Biol 13: 288-299. doi: 10.1111/j.1365-2486.2006.01278.x

Simoncelli F, Fagotti A, Dall’Olio R, Vagnetti D, Pascolini R, et al. (2005) Evidence of *Batrachochytrium dendrobatidis* infection in water frogs of the *Rana esculenta* complex in central Italy. EcoHealth 2: 307-312.

Simpkins C, Hero J-M, Van Sluys M (2010) Detecting the western limits for *Batrachochytrium dendrobatidis* in southeastern Queensland, Australia. Herpetol Rev 41: 454-456.

Skerratt LF, Berger L, Hines HB, McDonald KR, et al. (2008) Survey protocol for detecting chytridiomycosis in all Australian frog populations. Dis Aquat Org 80: 85-94.

Slough BG (2009) Amphibian chytrid fungus in Western Toads (*Anaxyrus boreas*) in British Columbia and Yukon, Canada. Herpetol Rev 40:319-321.

Smith KG, Weldon C, Conradie W, du Preez LH (2007) Relationships among size, development, and *Batrachochytrium dendrobatidis* infection in African tadpoles. Dis Aquat Org 74: 159-164.

Solís R, Lobos G, Walker SF, Fisher M, Bosch J (2010) Presence of *Batrachochytrium dendrobatidis* in feral populations of *Xenopus laevis* in Chile. Biol Invasions 12: 1641-1646.

Doi: 10.1007/s10530-009-9577-2

Soto-Azat C, Cunningham AA (2010) An invasive frog and endemic threatened amphibians: Impacts of chytridiomycosis in Chile. Conference abstract. EcoHealth 2010, Global Ecohealth Challenges; Multiple Perspectives, 18-20 August 2010, London, UK. pp. 75-76. Abstracts available online at: https://profileproductions.eventtrac.co.uk/system/attachments/168/original/EcoHealth_Final_online_programme_abstractbook.pdf

Soto-Azat C, Clarke BT, Poynton JC, Cunningham AA (2009) Widespread historical presence of *Batrachochytrium dendrobatidis* in African pipid frogs. Diversity Distrib 2009: 1-6. doi: 10.1111/j.1472-4642.2009.00618.x

Speare R, Berger L (2005) Chytridiomycosis in amphibians in Australia. Available at: http://www.jcu.edu.au/school/phtm/PHTM/frogs/chyspec.htm 26 January 2005. (2005).

Sredl M, Caldwell D (2000) Wintertime population surveys - Call for volunteers. Sonoran Herpetologist (Tucson Herpetological Newsletter) 13: 1.

Sredl MJ et al. (2002) Nongame and Endangered Wildlife Program Technical Report 208. Arizona Game and Fish Department, Phoenix, Arizona.

Stagni G, Scoccianti C, Fusini R (2002) Segnalazione di chytridiomicosi in popolazioni di *Bombina pachypus* (Anura, Bombinatoridae) dell'Appennino tosco-emiliano. IV Congresso della Societas Herpetologica Italica, a Ercolano.

Steiner SL, Lehtinen RM (2008) Occurrence of the amphibian pathogen *Batrachochytrium dendrobatidis* in Blanchard’s Cricket Frog (*Acris crepitans blanchardi*) in the U.S. Midwest. Herpetol Rev 39: 193-196.

Symonds EP, Hines HB, Bird PS, Morton JM, Mills PC (2007) Surveillance for *Batrachochytrium dendrobatidis* using *Mixophyes* (Anura: Myobatrachidae) larvae. J Wildl Dis 43: 48-60.

Talley BL, Lips KR, Ballard SR (2011) *Batrachochytrium dendrobatidis* in *Siren intermedia* in Illinois, USA. Herpetol Rev 42: 216-217.

Tatarian P, Tatarian G (2010) Chytrid infection of *Rana draytonii* in the Sierra Nevada, California. Herpetol Rev 41: 325-327.

Thompson PD, Fridell RA, Wheeler KK, Bailey CL (2004) Distribution of *Bufo boreas* in Utah. Herpetol Rev 35: 255-257.

Timpe EK, Graham SP, Gagliardo RW, Hill RL, Levy MG (2008) Occurrence of the fungal pathogen *Batrachochytrium dendrobatidis* in Georgia’s amphibian populations. Herpetol Rev 39: 447-449.

Todd-Thompson M, Miller DL, Super PE, Gray MJ (2009) Chytridiomycosis-associated mortality in a *Rana palustris* collected in Great Smoky Mountains National Park, Tennessee, USA. Herpetol Rev 40: 321-323.

Toledo LF, Britto FB, Araujo OGS, Giasson LMO, Haddad CFG (2006a) The occurrence of *Batrachochytrium dendrobatidis* in Brazil and the inclusion of 17 new cases of infection. S Amer J Herpetol 1: 185-191.

Toledo LF, Haddad CFB, Carnaval ACOQ, Britto FB (2006b) A Brazilian anuran (*Hylodes magalhaesi*: Leptodactylidae) infected by *Batrachocytrium dendrobatidis*: a conservation concern. Amph Rept Conserv 4: 17-21.

Tupper TA, Streicher JW, Greenspan SE, Timm BC, Cook RP (2011) Detection of *Batrachochytrium dendrobatidis* in anurans of Cape Cod National Seashore, Barnstable County, Massachusetts, USA. Herpetol Rev 42: 62-65.

Une Y, Kadekaru S, Tamukai K, Goka K, Kuroki T (2008) First reports of spontaneous chytridiomycosis in frogs in Asia. Dis Aquat Org 82: 157-160.

Velásquez-E BE, Castro F, Bolívar-G W, Herrera MI (2008) Infección por el hongo quitrido *Batrachochytrium dendrobatidis* en anuros de la Cordillera Occidental de Colombia [Infection by the chytrid fungus *Batrachochytrium dendrobatidis* in anurans of the Cordillera Occidental of Colombia] Herpetotropicos 4: 65-70 [in Spanish].

Venegas PJ, Catenazzi A, Siu-Ting K, Carillo J. (2008) Two new harlequin frogs (Anura: *Atelopus*) from the Andes of northern Peru. Salamandra 44: 163-176.

Venesky MD, Brem FM (2008) Occurrence of *Batrachochytrium dendrobatidis* in southwestern Tennesee, USA. Herpetol Rev 39: 319-320.

Vörös J, Price L, Donnellan SC (2011) *Batrachochytrium dendrobatidis* on the endemic frog *Litoria raniformis* in South Australia. Herpetol Rev 42: 220-223.

Waldman B, van de Wolfshaar K, Andjic V, Klena J, Bishop P et al. (2001) Chytridiomycosis and frog mortality in New Zealand. N Z J Zool 28: 372.

Walker S, Bosch J, James TY, Litvintseva AP, Valls JAO et al. (2008) Invasive pathogens threaten species recovery programs. Curr Biol 18: R853 - R854.

Weinstein SB (2009) An aquatic disease on a terrestrial salamander: individual and population level effects of the amphibian chytrid fungus, *Batrachochytrium dendrobatidis*, on *Batrachoseps attenuatus* (Plethodontidae). Copeia 4: 653-660.

Weldon C (2005) Chytridiomycosis an emerging infectious disease of amphibians in South Africa. PhD Dissertation. North-West University, Potchefstroom. Available: http://www.puk.ac.za/opencms/export/PUK/html/fakulteite/natuur/soo/drk/aacrg/chytrid/Chytrid-host_species_of_chytridiomycosis_in_Africa.doc. Accessed on August 6, 2007.

Weldon C, Du Preez LH (2004) Decline of the Kihansi Spray Toad, *Nectophrynoides asperginus*, from the Udzungwa Mountains, Tanzania. Froglog 62: 2-3.

Weldon C, Du Preez L, Vences M. (2008) Lack of detection of the amphibian chytrid fungus (*Batrachochytrium dendrobatidis*) in Madagascar. Mon Museo Regionale Scienze Naturali di Torino XLV: 95-106.

Woodhams DC, Vredenburg VT, Simon M-A, Billheimer D, Shakhtour B et al. (2007) Symbiotic bacteria contribute to innate immune defenses of the threatened mountain yellow-legged frog, *Rana muscosa*. Biol Conserv 138: 390-398.

Woodhams DC, Kilburn VL, Reinert LK, Voyles J, Medina D et al. (2008) Chytridiomycosis and amphibian population declines continue to spread eastward in Panama. EcoHealth 5: 268-274 doi: 10.1007/s10393-008-0190-0.

Yang H, Baek H, Speare R, Webb R, Park S et al. (2009) First detection of the amphibian chytrid fungus *Batrachochytrium dendrobatidis* in free-ranging populations of amphibians on mainland Asia: survey in South Korea. Dis Aquat Org 86: 9-13.

Young MK, Allison GT, Foster K (2007) Observations of boreal toads (*Bufo boreas boreas*) and *Batrachochytrium dendrobatidis* in south-central Wyoming and north-central Colorado. Herpetol Rev 38: 146-150.

Zellmer AJ, Richards CL, Martens LM (2008) Low prevalence of *Batrachochytrium dendrobatidis* across *Rana sylvatica* populations in southeastern Michigan, USA. Herpetol Rev 39: 196-199.

Zippel KC, Tabaka C (2008) Amphibian chytridiomycosis in captive *Acris crepitans blanchardi* (Blanchard’s Cricket Frog) collected from Ohio, Missouri, and Michigan, USA. Herpetol Rev 39: 192-193.
